# Hierarchy and ranking in fencing and tennis

**DOI:** 10.1038/s41598-026-53459-7

**Published:** 2026-05-31

**Authors:** Bogdán Asztalos, Boldizsár Balázs, Gergely Palla, Tamás Vicsek

**Affiliations:** 1 Department of Biological Physics, Eötvös Loránd University, Pázmány P. stny. 1/A, Budapest, 1117 Hungary; 2 Health Services Management Training Centre, Semmelweis University, Kutvölgyi út 2, Budapest, 1125 Hungary

**Keywords:** Hierarchy, Ranking, Sports networks, Sports prediction, Mathematics and computing, Physics, Psychology, Psychology

## Abstract

Ranking athletes by their performance in competitions and tournaments is common in every popular sport and has significant benefits that contribute to both the organization and strategic aspects of competitions. Although rankings are perhaps the most concise and most straightforward representation of the relative strength among the competitors, beyond this one-dimensional characterization, it is also possible to capture the relationships between athletes in greater detail. Following this approach, our study examines the networks between athletes in individual sports such as fencing and tennis, where the nodes are associated with the contestants and the edges are directed from the winner to the loser. We demonstrate that the connections formed through matches arrange themselves into a time-evolving hierarchy, with the top players positioned at its apex. The structure of the resulting networks exhibits detectable differences depending on whether they are constructed purely from round-robin data or from purely elimination-style tournaments. We find that although elimination tournaments lead to networks with strong hierarchical networks due to their organisational concept (structural hierarchy), the dominance differences between players (competing hierarchy) are less effective in this format, which is manifested in the increased probability of circular win-loss situations (cycles). The position within the hierarchy, along with other network metrics, can be used to predict match outcomes. In the systems studied, these methods provide predictions with an accuracy comparable to that of forecasts based on official sports ranking points or the Elo rating system. A deeper understanding of the delicate aspects of pairwise contest networks enhances our ability to model, predict, and optimise the behaviour of many complex systems, whether in sports tournaments, social interactions, or other competitive environments.

## Introduction

Quantifying the relative strength of competitors is a fundamental aspect of any sport, traditionally accomplished through linear ranking systems. However, these one-dimensional lists often oversimplify the complex web of relationships and performance dynamics that arise from direct, pairwise competition. Network science offers a robust framework for modelling complex systems by representing entities as nodes and their interactions as edges^1–5^. Applying this paradigm to the world of professional sports, where athletes are nodes and match outcomes are directed edges, provides a novel lens to analyse competitive hierarchies and predict future performance. By employing advanced analytical tools and methodologies such as centrality measures, motif analysis, and temporal network analysis, one can uncover the hidden features of complex networks such as the networks of matches. Understanding these delicate aspects of networks not only enriches our comprehension of the network’s structure and function but also enhances our ability to model, predict, and optimize the behaviour of complex systems, whether in sports tournaments, social interactions, or other competitive environments.

The present research examines key features of directed networks related to pairwise sports contests within tournament frameworks. We will use the terms“contest”and“match”, as well as“players”and “contestants”, interchangeably. The topic related to rankings and networks of competitors have recently become popular for interpreting results in the realm of sports tournaments^6–10^. As for rankings in general, the recent book by P. Érdi^11^ has an extensive overview of the many facets and applications of the field. While rankings are usually created by comparing the relevance of the entities considered, distilling the information gained into an order that faithfully reflects the results of these comparisons is a complex theoretical challenge. A common situation is when the more“important”of the two entities is given a higher score or rank, where importance can correspond to various features. For example, in sports, a contestant’s winning potential is considered a key feature. The task of ranking from pairwise comparisons has received significant attention in social choice theory^12,13^. This body of literature highlights that the transition from individual matches or comparisons to a global hierarchy is rarely straightforward, as evidenced by several impossibility theorems that illustrate the fundamental challenges and logical constraints of such systems^14–16^. For a broader perspective on these mechanics, Langville and Meyer^17^ provide a comprehensive overview of the mathematical algorithms used to rate and rank diverse subjects-ranging from sports teams and political candidates to products and Web pages-further emphasising that the methodology behind a ranking system is just as critical as the competitive data it seeks to organise.

In addition to linear (one dimensional) rankings, in the present work we also analyse the competitive relationships as a hierarchy, which provides a more nuanced and more complex ordering of players, with elite performers at the top of the hierarchy. In general, hierarchical organisation is a ubiquitous feature of complex networks^18–20^, observed in a remarkable range of systems, from intricate regulatory networks within cells^21,22^ and social structures of animal groups^23–25^, to the organisation of online news content^26^, scientific journals and scientific fields^27,28^, and even the grand scale of ecological systems^29,30^ and evolution^31–33^. In such networks, nodes positioned higher in the hierarchy typically have a greater influence than those at lower levels. Identifying and quantifying this hierarchical structure is a non-trivial challenge, with various approaches ranging from statistical inference based on network topology^18^ to the development of specific hierarchy measures^34–39^. Hierarchies are often represented as directed acyclic graphs that capture asymmetric relationships, such as parent-child or leader-follower, that define the hierarchical ordering of nodes.

This study investigates the comparative performance dynamics of tournament participants by analysing their historical trajectories against both direct opponents and common competitors. To ensure methodological robustness and assess the generalisability of the findings, the research focuses on two distinct athletic disciplines: fencing and tennis. While tennis provides a well-documented foundation for ranking and prediction analysis due to its global prominence^40–44^, fencing offers data granularity due to the round-robin stages of its tournaments. In these group phases, every contestant engages in a bout with every other member of the pool, providing a dense field of interaction for analysis. For the sake of terminological consistency, the term“matches”is utilised throughout this work to denote all competitive encounters, encompassing those specifically referred to as“bouts”within the fencing context.

Building upon established literature on the network properties of athletic contests^6,45–48^, this work constructs time-evolving networks to map the shifting relationships among players. These structures are examined from the perspective of hierarchical organisation and cycle structure, based on comparisons with random null models. A further goal is to discern potential structural disparities between networks derived from round-robin data versus those based strictly on elimination-style formats. In addition, the predictive accuracy of this network-centric framework is evaluated by benchmarking its results against traditional, score-based forecasting methods.

## Results

### Data

We collected a series of match results from various sports disciplines spanning specific periods. During our study, we worked with two sports—fencing and tennis—in which matches consist of a duel of two opposing individuals and end with the clear victory of one of them. Men and women compete separately in these two sports, and in fencing, there are also three disciplines (foil, épée, and sabre), each with its own independent competition. Matches where more athletes play together (e.g., tennis doubles or fencing team matches) were not included in the data, only the ones with an encounter of two individuals. Hence, we have studied 8 different competition categories, which are summarised in Table 1.

Table 1 also lists the type of events taken into account. In each category, we have limited the analysis to the main matches of the most elite events (i.e., qualifying rounds are not included) for two reasons: (i) The number of events increases drastically on lower levels of competitions, but due to their decreasing relevance, they are not recorded as rigorously in freely available databases as the more prestigious ones. (ii) On lower levels of competition, the differences between player abilities reduce, thus the reliability of ranking scores loses its significance.

**Table 1:** Sport result datasets we used for our analysis.

Sport	Considered events	Discipline	Sex	Number of matches	Source
Tennis	Grand Slams Tour-level events Davis Cup *from years 1968–2024*		Men	194,996	Jeff Sackmann^49^
			Women	158,092	Jeff Sackmann^50^
Fencing	Olympics World Championships Grand Prix World Cups Zone Championships *from years 2015–2024*	Foil	Men	54,629	Anya Post-Michaelsen^51^
			Women	43,798	
		Épée	Men	73,068	
			Women	55,290	
		Sabre	Men	49,458	
			Women	43,571	

### Time evolving network representation of sport matches

Based on past match results, we constructed networks where nodes represent players and directed links represent matches, pointing from the winner to the loser. If two players played against each other *n*-times with the same result, we assigned *n*-times more weight to the corresponding link. If the matches of the two players ended with different results, we represented them with two opposite links between the two nodes, each with the appropriate weight. The goal of these networks is to store the complex statistical information from past matches and use it to make inferences about relationships between players. Although all matches in the past carry information, it is not worth keeping all matches from the sport’s entire history because their relevance decreases over time: player abilities change, and an overwhelming victory over a much weaker player from several years ago does not indicate that the winner is still that much superior today. Hence, we define a network by taking matches into account only from the previous $t_\textrm{nb}$-long *network-building period*, in which match results presumably reveal something about the players’ current abilities. Sliding this network-building time window over time allows us to view the network as a time-dependent network that changes constantly, with its links at time *t* corresponding to matches from the time interval $[t - t_\textrm{nb}, t]$. In the following, we use the value of $t_\textrm{nb}= 1\,\textrm{year}$, in line with the fact that in both fencing and tennis, the official ranking is calculated based on performance over the last year.

**Fig. 1:**
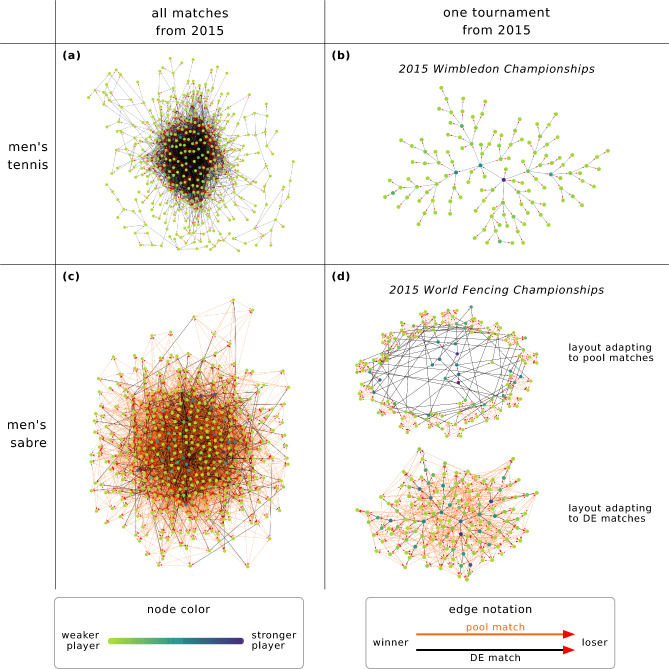
**Illustration of sport match networks.** In panels (**a**) and (**c**), we plotted the networks constructed from men’s tennis and men’s sabre matches in the year 2015, as described in the text. Although differences in their structural feature can be observed (the system of relations between nodes of the tennis network in panel (**a**) is apparent, while the relatively large number of pool matches makes the fencing network in panel (**c**) more complex), the core of both networks is too tangled and crowded for the human eye. Hence, in panels (**b**) and (**d**), we plotted the networks obtained from a single tournament. The organisational difference between the two sports is apparent: the tennis tournament is organised in a single elimination format, while the fencing tournament has two phases: a pool (group) phase and a direct elimination (DE) phase. In panel (**d**), there are two instances of the same network, differing only in their layout; one highlights the structure of the pool phase, and the other highlights the structure of the DE phase of the tournament.

For an illustration, we plotted a network of tennis matches and another network of fencing matches from one single year in Fig. 1. Besides, we plotted two networks in Fig. 1, one based on a tennis tournament and one on a fencing tournament, visualising the difference in their organisational methods.

### Elements of hierarchy in the network of matches

It is natural to assume a hierarchy among players in any individual sport, similar to a dominance hierarchy, where the best players are at the top and weaker players are lower. Our network construction is well-suited to capture this organization, as in the idealized scenario of stronger players consistently winning, the resulting networks become directed acyclic graphs, perfectly reflecting a hierarchical structure. On the contrary, in reality, stronger players cannot always win: surprising results and upset wins are not uncommon in sports, so the resulting graph will not be perfectly acyclic. In addition, there are non-transitive patterns in long-term rivalries^52^, and bogey team relationships when a team consistently underperforms against certain opponents^53^. However, the expectation regarding sports is still that match results reflect a kind of dominance, and the network defined above should exhibit some hierarchical structure.

#### Quantifying hierarchy in the network of matches

**Fig. 2:**
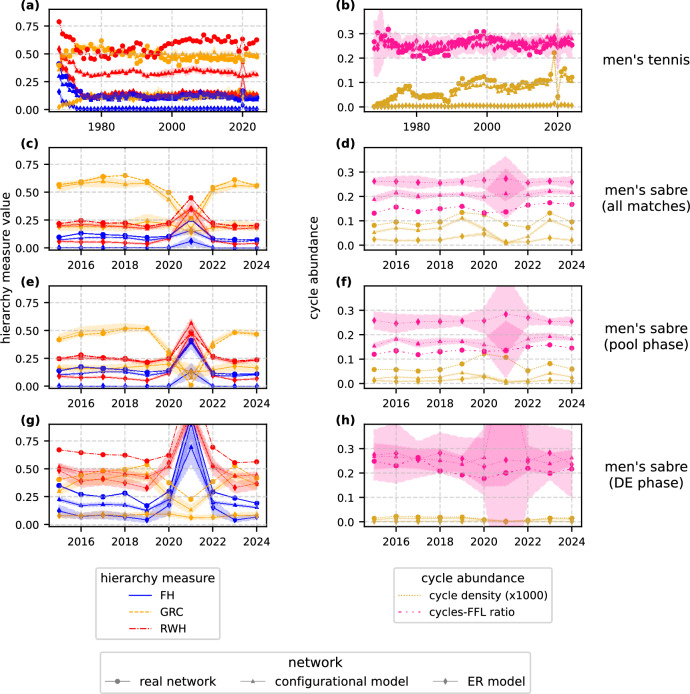
**Hierarchy measures and 3-cycle abundance in the network of matches.** (**a**) The flow hierarchy (blue), the global reaching centrality based on 2-reach (orange), and the random walk hierarchy (red) for the network of men’s tennis matches over time, displaying both the measured value in the original networks (circles) and also the average value in randomised networks according to the configuration model (triangles) and the Erdős–Rényi model (diamonds). The shaded regions around the averages indicate the 95% confidence interval. (**b**) The number of 3-cycles in the graph normed by the number all node triads (yellow), and its ratio to the number of feedforward 3-loops (FFLs) (pink) for the network of men’s tennis matches over time, displaying both the measured value in the original networks (circles), and also the average value in randomised networks according to the configuration model (triangles) and the Erdős–Rényi model (diamonds). The shaded regions around the averages indicate the 95% confidence interval. (**c**), (**d**) Same as (**a**)-(**b**) but for the network of men’s sabre matches. (**e**, (**f**) Same as (**a**–**b**) but for the network of matches played in the pool phase in men’s sabre tournaments. (**g**, **h)** Same as (**a**–**b**) but for the network of matches played in the direct elimination phase in men’s sabre tournaments.

To examine the extent to which the constructed networks exhibit signs of hierarchy, we employed three different hierarchy measures that capture distinct intuitions of hierarchy. In the case of flow hierarchy (FH)^54^ the fraction of links not taking part in any cycles is calculated, the global reaching centrality (GRC)^35^ is based on the inhomogeneity of the reaching centrality distribution, and the random walk hierarchy measure (RWH)^37^ uses a random walk process for discovering potential leaders. (A detailed description of the hierarchy measures used is given in the Methods.) In Fig. 2a and c, we plot these hierarchy measures over time for men’s tennis and men’s sabre. Here, we also compare the observed values with the results obtained in networks randomised according to the configuration model and the Erdős-Rényi model. (The analogous plots for the other categories are provided in the Supplementary Information.)

Apparently, in the case of men’s tennis (Fig. 2a), there is a large gap between the hierarchy measure of the original network and that of an Erdős–Rényi network (having the same number of nodes and links) for all years and all three hierarchy measures. In the meantime, comparisons with the configuration network ensemble yield mixed outcomes. The original networks exhibit higher FH and RWH values across all years, with a substantial difference in RWH. Conversely, the original network’s GRC values were either comparable to or, in some years, lower than the ensemble average. In the case of men’s sabre (Fig. 2c), we can observe a large gap between the hierarchy measures of the original networks and the average hierarchy in the corresponding Erdős–Rényi random networks, similarly to what we found for the men’s tennis networks. For the configuration model-based randomisation, the average hierarchy is again lower than the original value across all years and all hierarchy measures; however, this time, the difference compared to the original value is smaller in magnitude.

For both of these networks, we can observe a significant impact of the Covid pandemic in 2020–2021. During this period, sports federations imposed strict regulations on how tournaments should be organised, which had a drastic effect on the number of matches. For example, in our dataset, there were about 2,800–3,000 men’s tennis matches each year in the decade before the pandemic. In 2020, there were only 1,462 matches, while in 2021 there were 2,733, so things almost returned to normal after one year. In fencing, however, being an indoor sport, more severe policies had to be implemented, which had a larger impact: there are about 6,000-6,500 men’s sabre matches each year before Covid, but in 2020, there were 2,423 matches, then 739 matches in 2021, and still only 4,676 matches in 2022. Naturally, these impacts affect not just the size, but the structure of the sports networks, and this can be seen in the panels of Fig. 2 as anomalous peaks in our analysis. Since sports events did not function normally during these years, we do not intend to draw general conclusions from the results in this period.

The comparison between the results for men’s tennis networks and the men’s sabre networks is inconclusive: the RWH values are higher for tennis, the GRC values are higher for sabre, and the FH values are roughly equal. This incomparability can potentially be explained by the differences in the implementation of tournaments between the two sports. Conventionally, tennis tournaments are organised in a single-elimination format, where the loser of each match is eliminated from the tournament, thereby creating a tree-shaped subgraph of the participants. This means that the winner of each tournament is the dominant node of the tournament’s sub-graph, and the overall structure of the whole network will consist of these sub-trees, dominated by the few best players who have won (or almost won) all the tournaments they started in. On the contrary, fencing tournaments begin with a *pool phase*, where participants are divided into pools of 5–7 players, and they play against all the other players in the pool, aiming to earn the right to proceed to the *direct elimination phase*, for which only the 75–85% of competitors qualify.

Both tennis tournaments and the direct-elimination phases of fencing tournaments use seeding to prevent too strong contenders from clashing too early, but the two seeding methods differ in some details. In fencing, the direct elimination table is deterministic: the number *i* seed is to fence with the $2^k+1-i$ seed amongst the best $2^k$ round (i.e., in the quarter-finals, the rank-1 fences with rank-16, rank-2 with rank-15, etc., unless an upset happens earlier)^55^. In tennis, only the best 16 or 32 competitors are seeded, and their position is randomised on the above, fencing-like, deterministic direct elimination table scheme. For example, rank-1 and rank-2 are fixed in their places in the final, but rank-3 has a 50-50% chance of playing against rank-1 or rank-2 in the semifinals. Similarly, rank-5, 6, 7, and 8 have 25% chance of playing any of the top 4 in the quarter-finals, and so on. Unseeded players are randomised throughout the remaining empty spots of the elimination table^56^. The ranking order that determines the seeding is also an important difference between the two sports: in tennis, the seeding is based on the official sports ranking, while in fencing tournaments, it uses the pool phase’s winning percentage performances (with tie-breaking aspects in order: touché-difference, more touchés given, coin toss). Consequently, every pool match and every touché is relevant for the competitors’ future path on the direct elimination table, preventing competitors from losing motivation towards the end of the pool phase, which would otherwise cause a possibility of match-fixing, and corrupt statistical analysis^57–59^.

The round-robin phase of fencing tournaments facilitates the formation of cycles in the graph, as players in the same pool will inevitably create a weakly linked clique, thereby undermining the network’s hierarchical structure. Also, although there is more possibility to form cycles during the pool phase, there is also a wider ability range between players within a pool, resulting a lower likelihood of upset wins with respect to the DE phase, which involves a more homogeneous set of stronger players. Consequently, to get a clearer picture of hierarchy within these sports, we need to separate the following two factors of the formation of network hierarchy: (1) the organisational structure of the tournament format (structural hierarchy), and (2) the differences in ability and attitude towards competition between the players (competing hierarchy). This includes the dominance hierarchy of players, but can also incorporate any form of hierarchy which is independent of the organisational structure.

To examine the difference between the round-robin format and the direct elimination format, we directly compared the pool phase and the direct elimination phase of sabre tournaments directly to each other. In Fig. 2e and g, we recreate the plots of panels Fig. 2c for networks built of matches only from the pool phase and the direct elimination phase, respectively. FH and RWC values are firmly larger for the direct elimination network, but so are they for the networks generated with the configuration model, suggesting that this is the trace of the structural hierarchy due to the tree-shaped skeleton of the graph. Let us compare the hierarchy measures of real networks to those of homogeneous Erdős-Rényi networks. The deviation is larger for all three measures in the pool phase networks, which can be interpreted as indicating the presence of the competing hierarchy, i.e., the superior-subordinate relationships among pool matches were more decisive. The quantitative comparison of the real and the randomised data is provided in Figs. S1–S8 in the Supplementary Information, where we plotted the ratio and the z-score of each hierarchy measures for each sports category.

To test the conjecture that the level of hierarchy of the networks of games depends on the way the tournaments are organized, we examined directly the number of cycles in the networks. Within an ideal hierarchical structure of edges, the directions of links determine a one-way route from dominant nodes to subordinate ones, and the possibility of returning from a subordinate node to a dominant one would imply the breaking of the hierarchy. Hence, the lack of circular triads i.e., paper-rock-scissors-like relationships between players, is a clear trace of player dominance. In Fig. 2b, d, f, and h, we measure how abundantly 3-cycles are present in the studied graphs. The cycle abundance is calculated by comparing the number of 3-cycles in the graphs to all possible node-triads in the graph (cycle density), or to all weakly linked node-triads (cycle-FFL ratio), including 3-cycles and feedforward 3-loops (that correspond to a specific arrangement of 3 directed links between 3 nodes obtained by reversing a single link in a cycle). (The detailed description of the cycle abundance measures is given in the Methods.) Apparently, for networks of tennis matches (Fig. 2b) and for networks of the DE phase of sabre tournaments (Fig. 2h) the real cycle-FFL ratio does not differ significantly from the randomised networks (neither in the case of the configuration model, nor in the case of Erdős–Rényi model), but for networks where pool matches contribute, the cycle abundance is lower. This suggests that competing hierarchy does not stand out in the single-elimination format, and the degree of hierarchy we observe in this case is the structural hierarchy stemming from the brackets of the tournaments. The quantitative comparison of the real abundance values and the randomised ones is provided in Figs. S9–S16 in the Supplementary Information, where we plotted the ratio and the z-score of each abundance measure for each sports category.

**Fig. 3:**
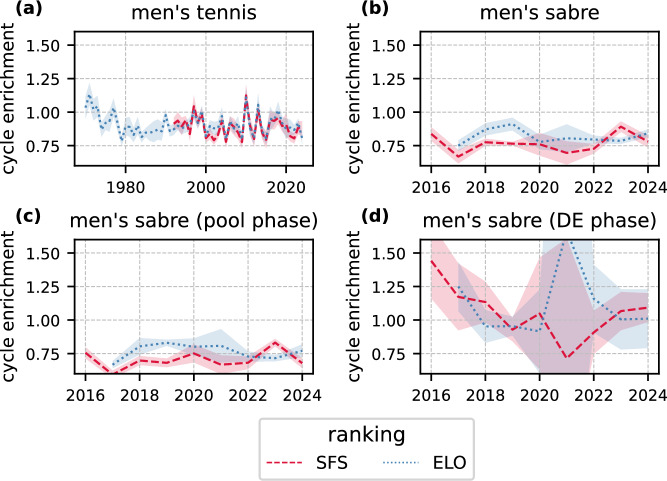
**Cycle enrichment calculated with SFS and ELO rankings in the network of matches.** The fraction of the number of 3-cycles and the expected number of 3-cycles from a given player strength distribution. The shaded regions around the averages indicate the 95% confidence interval. (**a**) The cycle enrichment values for the network of men’s tennis matches over the years. (**b**) The cycle enrichment values for the network of men’s sabre matches over the years. (**c**) The cycle enrichment values for the network of matches played in the pool phase in men’s sabre tournaments. (**d**) The cycle enrichment values for the network of matches played in the direct elimination phase in men’s sabre tournaments.

We also compared the number of 3-cycles in networks to their expected number with a given strength distribution. In the following subsections of the Results, we explain how player strengths can be measured using traditional methods or the concept of hierarchy, and how outcome probabilities can be estimated accordingly. If we resample the result of each match based solely on the probabilities coming from the strength difference of players, the number of cycles can be considered as an expected value following the specific dominance and organization situation. Since all the information used for this resampling process is gained from past data, this expectation can be evaluated in real-time, without any external knowledge. If we observe fewer cycles in the networks of played matches, the competing hierarchy has a more substantial effect on the real system. However, if the number of cycles is higher, it means that more upset wins occurred, and the assumed dominance between players could not prevail. We measured the enrichment of the network with 3-cycles by calculating the fraction of cycles and the expected value using a given ranking score. (The detailed description of the cycle enrichment measure is given in the Methods.) In Fig. 3, we plot this enrichment value over time with two different ranking scores for both men’s tennis and men’s sabre. (The analogous plots for the other categories and ranking scores are provided in the Supplementary Information.)

They give roughly the same results: for both tennis and sabre data, the enrichment value is smaller than 1 in Fig. 3a and b, with sabre values being slightly smaller, suggesting that competing hierarchy in sabre has larger relevance. In Fig. 3c and d, we can see that the cycle enrichment values are significantly larger for DE matches, which means that in the elimination format, dominance is less effectively present, and more upset wins happen between players.

#### Structure of hierarchy

One of the most essential features of a hierarchical system is that a few superior agents dominate over many subordinate ones. This means the system must have a widening shape from top to bottom, as there are more units on the lower levels of the hierarchy than on the upper levels. However, the exact structure of this widening can depend on several factors. The simplest case of hierarchy is when each superior dominates over the same number of subordinates, which results in an exponential multiplication of the number of nodes from level to level, but real hierarchical organisations can show different structures depending on the specific relational structure behind it. In the following, we examine if the networks of sports matches have such a widening shape.

**Fig. 4:**
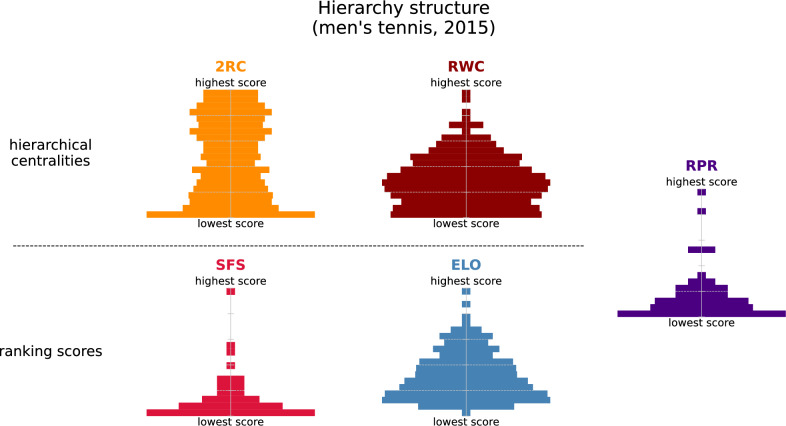
**The structure of the hierarchy for men’s tennis.** The distribution of nodes along the 2-reach centrality (orange), the random walk centrality (red), the official sport ranking (pink), the Elo score (cyan), and the reversed PageRank (purple) for the network of men’s tennis matches played in 2015. The width of the bars represents the relative number of nodes having a specific centrality value on log-scale.

**Fig. 5:**
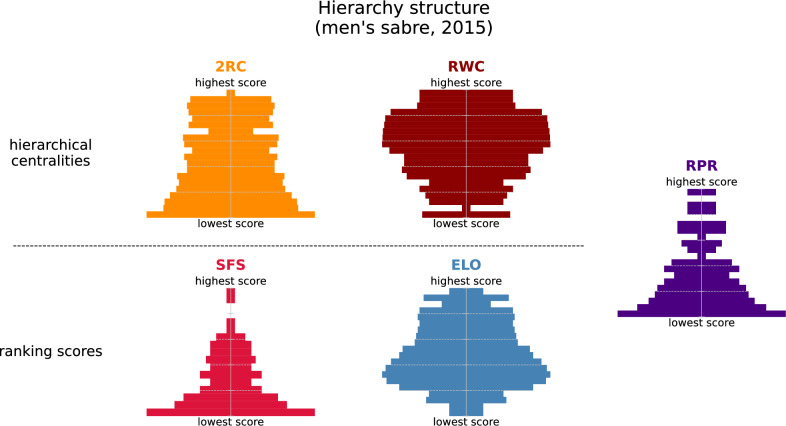
**The structure of the hierarchy for men’s sabre.** The distribution of nodes along the 2-reach centrality (orange), the random walk centrality (red), the official sport ranking (pink), the Elo score (cyan), and the reversed PageRank (purple) for the network of men’s sabre matches played in 2015. The width of the bars represents the relative number of nodes having a specific centrality value on log-scale.

To measure the widening of the hierarchy across levels, we first need to define a method to measure the level of each separate node and determine the distribution of nodes according to this quantity. Due to the intuitive character of hierarchy, defining the position of nodes within it can also not be done universally, and different measures can be found along different approaches. We used 2 different node centralities (or local measures) to capture how dominant their role is within the hierarchical structure of the network, the 2-reach centrality (2RC)^60^, counting what portion of all nodes of the network can be reached within two steps from the given node, and the random walk centrality (RWC)^37^, taking the probability that a random walker discovering potential leaders stays on the given node. (A detailed description of the hierarchy measures used is given in the Methods). In the upper row of Figs. 4 and 5, the distribution along these two quantities is shown for men’s tennis and men’s sabre in a vertical view visualising the widening of the hierarchical structure (analogous plots for other sport disciplines are provided in the Supplementary Information). Although a certain extent of widening can be observed in some cases, there is no universal pattern for the structures of the network structure along the hierarchical centralities.

### Ranking

Associating points or scores with the winning potential of a sports person or a competing individual has a history dating back to the middle of the last century^61–65^. In close relation, ranking competitors in sports and other competitive fields has several benefits that contribute to both the organization and strategic aspects of competitions. Ranking gives a quantifiable measure of a competitor’s performance relative to others. This enables competitors, coaches, teams, investors, and sponsors to evaluate their strengths and weaknesses in comparison to their peers. Rankings are also crucial for creating fair matchups in tournaments. By seeding competitors based on their rankings, organizers can ensure that the strongest competitors do not meet until later in the competition, which maintains competitive interest and fairness throughout the event. In addition, rankings can serve as a motivational tool for competitors by setting benchmarks and goals. Achieving a higher ranking can be a significant accomplishment, providing recognition and validating the effort and skill of a competitor. From a research perspective, rankings provide valuable data for analysing trends, predicting outcomes, and studying the dynamics of sport at various levels. This can enhance the understanding of how different factors influence competitive success.

A well-known example for ranking players is the Elo ranking^62,63^, a method widely used both in practical applications and research^64–66^. It is based on the formulas of the Bradley–Terry model^67^ with specific parameters, assuming that the probability of victory of player *i* over player *j* is

1 $$\begin{aligned} \textrm{Pr} (i\text { wins over }j) = \frac{1}{1 + 10^{(s_j - s_i) / S}}, \end{aligned}$$

where $s_i$ and $s_j$ are the Elo scores of players *i* and *j*, and *S* is an arbitrary parameter. The value of *S* is usually chosen to ensure the score differences can be measured in whole numbers. In chess, for example, *S = 400* is preferred^68^, while in football, FIFA World Ranking uses *S = 600*^69^. As Jerdee and Newman suggested in their work^70^, this formula can be extended with two parameters:

2 $$\begin{aligned} \textrm{Pr} (i\text { wins over }j) = \frac{1}{2} \alpha + (1 - \alpha ) \frac{1}{1 + \textrm{e}^{\beta (s_j - s_i)}} \end{aligned}$$

where *α* quantifies probability of an extreme upset win, and *β* represents the depth of the competition.

#### Ranking scores and hierarchical centralities

The most important and most interesting question in any sport is who the absolute best player is, or in a more general way, what is the order of players based on their abilities. Similarly to hierarchy, playing ability is also an intuitive notion, and finding a formal definition or method of measurement for it can be done in several different ways depending on what kinds of performance we consider as the signs of the absolute greatness. Nevertheless, due to the cultural needs of fans, most sports have an official ranking system, scoring player’s performance according to certain criteria which reflect the sport’s legacy in a more or less objective way, so in this paper, we use the official sports federation’s ranking score (SFS) for each sport as one of the studied ranking scores. Besides, we also calculated the Elo score (ELO) for each player based on the matches in the database, since it is widely viewed as one of the most reliable ranking scores. (The details of its calculation are given in the Methods.) It is essential to note that datasets used do not contain all matches of the sport. Hence, the actual Elo scores of the players, which would need to take all previous matches into account by definition, cannot be calculated; the scores we gained from these elite matches can be used only for comparison within this elite group of players. The magnitude of the difference caused by a limited set of matches is discussed in the Supplementary Information.

Let us notice that besides these two classical direct performance-awarding score systems, any quantity can be interpreted as ranking score that generally assigns higher values to players who win more matches. Hierarchical centralities, for example, measure the role of nodes (players) within the hierarchical structure of the network of matches, which can be developed by gaining victories, hence hierarchical centralities should also be able to serve as ranking scores. This can be justified by the intuition of sport dominance having similar attributes to the dominance in a social relation system. Conversely, classical ranking scores are also suitable to examine the structure of hierarchy since the distribution of players along a given score can counted independently on the network itself as shown in Figs. 4 and 5.

As an illustration of the similar but not identical intuition behind different ranking scores, Tables 2 and 3 present the top 5 players according to the various ranking scores for men’s tennis and men’s sabre in 2015. It can be observed that the specific order varies through the separate lists, some particularity appears almost everywhere, for example, Novak Djokovic was clearly the best tennis player in that year according to four out of the five rankings, or Áron Szilágyi and Junghwan Kim were among the best 5 sabre player according to three out of the five rankings.

**Table 2:** The first 5 players of men’s tennis according to different local measures as ranking score in 2015.

2RC			RWC		
Rank	Player	Score [$\cdot 10^4$]	Rank	Player	Score [$\cdot 10^6$]
1	Novak Djokovic	5561	1	Marcelo Arevalo	9469
2	Andy Murray	5444	2	Marton Fucsovics	8985
3	Rafael Nadal	5421	3	Mohammad Ghareeb	7636
4	Stan Wawrinka	5374	4	Chieh Fu Wang	7545
5	John Isner	5304	5	Patrick Tierro	6917

SFS			ELO		
Rank	Player	Score	Rank	Player	Score
1	Novak Djokovic	16585	1	Novak Djokovic	2416
2	Andy Murray	8945	2	Roger Federer	2267
3	Roger Federer	8265	3	Andy Murray	2189
4	Stan Wawrinka	6865	4	Rafael Nadal	2134
5	Rafael Nadal	5230	5	Stan Wawrinka	2071

RPR		
Rank	Player	Score [$\cdot 10^5$]
1	Novak Djokovic	6125
2	Roger Federer	5197
3	Andy Murray	3360
4	Stan Wawrinka	3114
5	Rafael Nadal	2133

**Table 3:** The first 5 players of men’s sabre according to different local measures as ranking score in 2015.

2RC			RWC		
Rank	Player	Score [$\cdot 10^4$]	Rank	Player	Score [$\cdot 10^6$]
1	Luca Curatoli	7635	1	Dmytro Raskosov	3375
2	Bongil Gu	7190	2	Francesco d’Armiento	3361
3	Matyas Szabo	7166	3	Kamil Ibragimov	3351
4	Renzo Agresta	7119	4	Luigi Samele	3334
5	Bolade Apithy	7119	5	Áron Szilágyi	3320

SFS			ELO		
Rank	Player	Score	Rank	Player	Score
1	Junghwan Kim	243	1	Nicolas Limbach	1813
2	Áron Szilágyi	219	2	Junghwan Kim	1790
3	Vincent Anstett	181	3	Bongil Gu	1782
4	Alexey Yakimenko	169	4	Tiberiu Dolniceanu	1771
5	Nikolay Kovalev	151	5	Max Hartung	1769

RPR		
Rank	Player	Score [$\cdot 10^5$]
1	Kamil Ibragimov	1969
2	Bongil Gu	1883
3	Max Hartung	1751
4	Áron Szilágyi	1749
5	Junghwan Kim	1662

Although the above hierarchical centralities can be considered as ranking scores, their definition was adapted to a network-based point of view. Hierarchical centralities are typically designed for systems, where knowing the domination status of different agents has a global advantage (e.g. animal groups should know who the leader is, companies should have a clear subordination organisation). In sports, however, the apparent dominance of a single person does not have a social profit, in fact, the opposite: if one player always wins, the sport becomes boring. Hence, it would be worth using a network quantity that measures social dominance directly, being relevant in sports, instead of mathematical intuitions of network hierarchy.

For this, let us consider the *prestige* gained by winning a match as something illustrious and triumphant, that is worth chasing. If one catches this *prestige*, their goal is not to lose it. Suppose that if a player loses a match, the *prestige* in the possession of this player goes to the winner. If a defeated player wants to regain some *prestige*, they must win matches again. Also, *prestige* is fleeting, so it decreases in time and restarts its journey among players from time to time, so players with high reputations need to continue winning to keep their status. In this picture, *prestige* can be described as a walker on the network of players which steps in the direction of victories. Arcagni et al.^45^ interpreted this as the match being a skill transfer from the loser to the winner, and the player’s ability should be measured by an eigenvalue centrality in a directed network, where the direction of links must be reversed compared to our definition. In this paper, we used the simplest eigenvalue centrality, the PageRank algorithm, designed by the founders of Google, Brin and Page^71^. Figures 4 and 5 also show the distribution of nodes along reversed PageRank (RPR). In the following, we use this as a fifth local measure, which is both a hierarchical network centrality and a ranking score.

#### Decay of ranking score

**Fig. 6:**
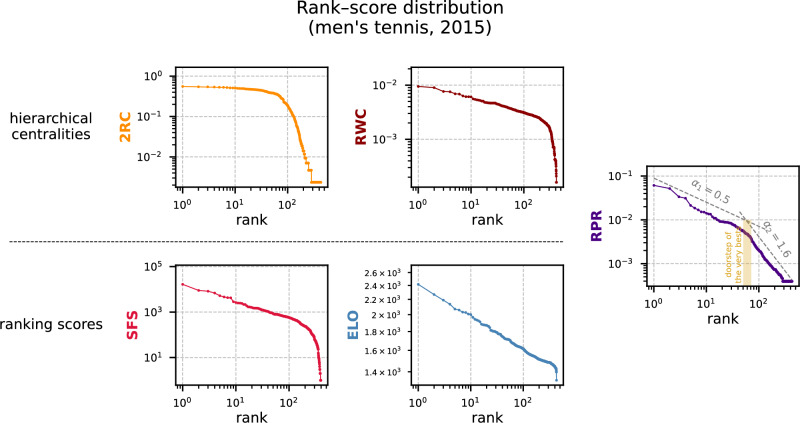
**The rank–score distribution of local measures for men’s tennis.** The rank–score distribution of nodes along the 2-reach centrality (orange), the random walk centrality (red), the official sport ranking (pink), the Elo score (cyan), and the reversed PageRank (purple) for the network of men’s tennis matches played in 2015. In the case of reversed PageRank two segments can be found following power-law scaling.

**Fig. 7:**
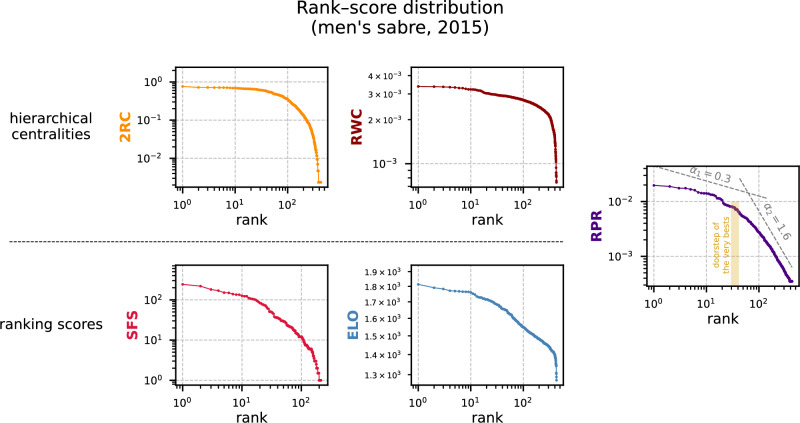
**The rank-score distribution of local measures for men’s sabre.** The rank–score distribution of nodes along the 2-reach centrality (orange), the random walk centrality (red), the official sport ranking (pink), the Elo score (cyan), and the reversed PageRank (purple) for the network of men’s sabre matches played in 2015. In the case of reversed PageRank two segments can be found following power-law scaling.

Since each player has several local measures, it can be asked how they relate to each other. Is the best player an outstanding talent, or is there a close competition for the first place? Are there more players with lower score values, or does the density of players remain the same at each level of competition? To answer these questions, we plotted the rank–score distribution of the different ranking scores in Figs. 6 and 7 for men’s tennis and men’s sabre, respectively (analogous plots for other sport disciplines are provided in the Supplementary Information). It can be generally observed that on a log-log scale, hierarchical centralities (2RC and RWC) start with a long plateau and fall sharply, Elo score values all are in the same order of magnitude, and the decay of SFS shows some concave curve. The distribution of RPR values, on the other hand, shows power-law decay through more than one order of magnitude for all studied sports disciplines, and in most cases, more segments can be observed realising power-law decay with different power exponents. Similar phenomena occur in other fields of complex systems where power-law distributions are the consequence of the inner interaction of the system, and the breaking point of the curve marks a transition point characteristic to the effect.

In Figs. 6 and 7, we fitted the power exponents on the decay of the RPR score in each regime where the power-law decay is a good model. Where these trends change, the existence of some remarkable cognitive reason relating to sports ranking is suggested in the background. Therefore, our findings show that RPR is suitable for measuring hierarchy from the perspective of such a complex interactive effect and for identifying the point that separates the very best players from other elite athletes according to this effect.

### Prediction using score differences

As mentioned above, a ranking score records the order of dominance between players and suggests that a player with a higher rank in a pairing should win the match. However, in sports, upset wins often occur, sometimes due to pure luck but sometimes because better rank does not indicate being in better shape under the given circumstances. Thus, a ranking score does not tell us everything about a future sports event just provides us with a hint about who is more likely to win. Hence, the quality of a ranking system can be characterised by how accurately estimates the probability of the different outcomes of a match. A good example of this is the Bradley–Terry model^67^ which is explicitly designed in such a way that the exponential ranking scores must be proportional to the chance of victory, and the difference between opponents gives a tangible estimate for the probability of the outcomes through formula (1). Since the rank order of players can be composed based on any quantity defined for players, a natural question is how well certain quantities predict match outcomes. In this section, we examine all the 5 local measures defined above in the light of prediction and compare their accuracy to each other.

We used formula (2) for calculating winning probabilities, and fitted the optimal parameters $\hat{\alpha }_s (t)$ and $\hat{\beta }_s (t)$ in each year *t* for each score *s* then, we used again formula (2) with the parameter values $\alpha = \hat{\alpha }_s (t)$ and $\beta = \hat{\beta }_s (t)$ to predict result of next year (*t + 1*) matches. The details of the fitting and prediction procedure are explained in the Methods section. We calculated the outcome probabilities of all matches which had sufficient prior information in all 8 competition categories with all 5 ranking scores and compared the probability estimates to the real results of the matches, which we know so in retrospect. We measured the accuracy of different prediction methods through the fraction of mistakes (FM), the average squared error (SE), and the average linear error (LE) of the calculated probabilities, and even the area under the ROC curve of the prediction procedure (AUC) (see the formulas in Methods). In Table 4, the accuracies of different prediction scores are shown among the other competition categories using the different ranking scores. For tennis, Elo scores performs the best, but for fencing disciplines, SFS is the most accurate measure. This means that the official ranking method in fencing is more efficient than in tennis, whose official ranking method underachieves even some of the hierarchy measures.

**Table 4:** Prediction accuracy in different sports disciplines using different centralities as the “prediction score”.

Centrality	FM	SE	LE	AUC	Centrality	FM	SE	LE	AUC
Men’s tennis					Women’s tennis				
2RC	0.345	0.219	0.437	0.657	2RC	0.347	0.219	0.436	0.659
RWC	0.450	0.245	0.489	0.550	RWC	0.437	0.243	0.485	0.565
SFS	0.356	0.224	0.447	0.644	SFS	0.350	0.223	0.442	0.655
ELO	**0.344**	**0.213**	**0.426**	0.657	ELO	**0.339**	**0.209**	**0.417**	**0.663**
RPR	0.345	0.217	0.433	0.658*	RPR	0.344	0.215	0.429	0.661
Men’s sabre					Women’s sabre				
2RC	0.320	0.209	0.408	0.689	2RC	0.347	0.221	0.435	0.661
RWC	0.374	0.229	0.457	0.632	RWC	0.400	0.235	0.468	0.604
SFS	**0.272**	0.194	0.386	**0.734**	SFS	**0.296**	0.205	0.411	**0.708**
ELO	0.297	**0.194**	**0.384**	0.706	ELO	0.320	0.205*	**0.407**	0.682
RPR	0.317	0.207	0.414	0.693	RPR	0.340	0.217	0.435	0.667
Men’s épée					Women’s épée				
2RC	0.360	0.224	0.445	0.649	2RC	0.351	0.222	0.436	0.659
RWC	0.412	0.239	0.475	0.593	RWC	0.400	0.237	0.472	0.606
SFS	**0.314**	**0.213**	**0.428**	**0.692**	SFS	**0.294**	**0.204**	**0.410**	**0.711**
ELO	0.343	0.215	0.432	0.660	ELO	0.324	0.206	0.411	0.678
RPR	0.359	0.225	0.449	0.650	RPR	0.350	0.220	0.441	0.659
Men’s foil					Women’s foil				
2RC	0.327	0.210	0.416	0.683	2RC	0.329	0.213	0.417	0.682
RWC	0.377	0.230	0.461	0.630	RWC	0.371	0.231	0.455	0.636
SFS	**0.278**	**0.197**	**0.395**	**0.727**	SFS	**0.278**	0.198	0.394	**0.726**
ELO	0.306	0.197	0.396	0.697	ELO	0.304	0.197*	**0.390**	0.699
RPR	0.325	0.209	0.423	0.685	RPR	0.326	0.210	0.421	0.683

The best accuracy value is highlighted in bold for each category and accuracy measure. If the best value does not differ significantly from the second best, it is marked by an asterisk (*). The best score to use for prediction is usually ELO or SFS, but this depends on the sport and the method used to measure error. (In the Supplementary Information we discuss the statistical reliability of the values).

### Differences between men’s and women’s categories

A natural question is whether there is any difference between men’s and women’s sports. In order to keep different disciplines and categories comparable, we performed the exact same analysis on all 8 datasets from Table 1. Although Figs. 2, 3, 4, 5, 6 and 7 show only the results for men’s tennis and men’s sabre due to space efficiency, the results for the other 6 datasets are included in the Supplementary Information, and the analogous plots can be found in Figs. S1–S55. With the help of this additional material, the findings for different sexes can be compared directly to each other, as well as for different disciplines. We found, however, that the difference between sports disciplines is more decisive than the difference between sexes, and men’s and women’s categories of the same discipline always show roughly the same qualitative behaviour within the significance level. Hence, from the perspective of our analytical procedure, there is no observable difference between the competition among men and the competition among women.

## Discussion

In the highly competitive world of popular sports, like professional tennis, public perception often gravitates towards a well-defined hierarchy of elite players. Dominant figures in the sport, frequently spotlighted in the media, are perceived as nearly invincible against opponents with lower ATP ranks. This traditional view suggests a predictable landscape in which top-ranked players constantly outperform their lower-ranked opponents. However, our analysis of the network of matches tells a strikingly different story.

Utilizing a network-based approach, where nodes represent players and directed edges indicate match outcomes from winner to loser, we uncover a more nuanced pattern in the data. Despite the clear hierarchy suggested by ATP rankings and public perception, the frequency of lower-ranked players defeating their higher-ranked peers is not negligible at all. This observation is exemplified by the significant number of cycles within our network, constructed from matches among the top 1000 ranked players in a given year. We see a similar phenomenon in the different disciplines of fencing. This paradox challenges the conventional wisdom of player dominance in sports where strong players are inaccessible, and illuminates the intricate dynamics of competition that the traditional interpretation of standard rankings as transitive orders fails to capture. The distinction between structural hierarchy and competing hierarchy is necessary to get a clear picture, and measure the real strength of players. Such findings not only enhance our understanding of sports competitions but also provide valuable insights into the nature of hierarchy and performance in any competitive organization. By revealing the underlying complexity and unpredictability of outcomes, our study encourages a reevaluation of how success and potential are assessed in competitive fields.

The implications extend beyond sports, suggesting that in any hierarchical organization, apparent stability and predictability might mask a dynamic interplay of factors that could lead to unexpected outcomes. Thus, our work contributes to a deeper understanding of the elements that govern success and the potential for disruption in established hierarchies.

When analysing networks of matches in competitive sports and other similar systems where pairwise elimination is used to determine a winner, it is important to consider also the details of the tournament format that may affect the network structure in both direct and implicit forms. Elimination tournaments, where each round halves the number of participants, inherently create a pyramid-like hierarchy where the fewest compete the most. This method of elimination ensures that the final rounds are contested by those who have survived multiple rounds of competition, which might suggest they are the strongest or most skilled participants. In parallel, players who advance further in the tournament naturally have more opportunities to score points, which could skew rankings toward those who play more matches, rather than those who might be more skilled but were eliminated early due to the tournament draw or early tough matchups. However, the official ranking system may also aim to encourage players to play more matches, because the revenue of the organiser depends on this. According to that, besides finding the best player, as a side effect, tournaments may also amplify the perceived difference in skill or strength between tiers of players based on the cumulative scoring system. This can be seen as both a feature and a bug of tournament design-effective for determining a winner but potentially misleading in evaluating a player’s true strength across the board.

The tendency of knockout structures to obscure true merit was pointed out already in earlier works in tournament design literature^72–74^. Our research reinforces these concerns, demonstrating that the“last standing”survivor of a bracket is not always synonymous with the highest-performing participant. This systemic inaccuracy is relevant not only to sports but also to any competitive field that employs a similar elimination-based structure to rank participants, such as gaming, business competitions, or academic contests. Our results, together with the established literature encourage a deeper examination of how we design competitive systems and what modifications might be needed to ensure they are fair and truly reflective of individual abilities and strengths. This could lead to exploring alternative tournament formats that might offer a more balanced approach to competition and ranking.

## Methods

### Hierarchical centralities and hierarchy measures

The intuition of a hierarchical network is that there are a few dominant nodes which have superior relationships with the others from some point of view. Since this intuition can be formalised in many different ways, different approaches have been used to describe the hierarchy in networks. Some of them are applicable both on a global level, characterising the hierarchy of the entire network, and on a local level, characterising the role of individual nodes within a hierarchical structure. In this paper, we discuss both levels.

On local level, we used the following hierarchical centralities:

*m-reach centrality (mRC)* is the number of nodes which can be reached in *m* steps starting from a certain node. It can be viewed as an extension of node degree since degree is the number of direct neighbours of the certain node, so it corresponds to *m = 1*^60^. In this paper, we used *m = 2* (noted by 2RC), and normed the value with the number of all reachable nodes of an unweighted network would be

$$\begin{aligned} \textrm{2RC} (i) = \frac{1}{N - 1} \cdot \# \{\text {reachable nodes from }i\text { in }2\text { steps}\} \end{aligned}$$

where *N* is the size of weakly connected component in which node *i* is present. Since sports networks are weighted in our case, we used the weighted extension of *m*-reach centrality suggested by Mones et al.^35^. This also incorporates the total weight of the possible directed paths into the calculation.

*Random walk centrality (RWC)* is based on a random walk model on the network with an assumed information flow over the links. By seeking the source of the information, the random walkers travel backwards on the links, and also take into account that a piece of information has a lower likelihood to originate from an in-neighbour with large out-degree compared to a in-neighbour with low out-degree. Accordingly, the transition matrix $T_{ij}$ describing the probability of the walker going to node *i* from *j*, depends on the degrees of both nodes *i* and *j*, and also on an arbitrary parameter *f*, which describes the characteristic distance made by the walker. In the present paper, we used the weighted version of the transition matrix, where the degree of the nodes is replaced by their strength. According to the considerations of Czégel and Palla^37^, the stationary probability distribution $\boldsymbol{p}^{\textrm{stat}}$ of the walker is given by the eigenvalue problem

$$\begin{aligned} \boldsymbol{p}^{\textrm{stat}} = \frac{1}{1 + f} \left[ \boldsymbol{T} \left( \boldsymbol{p}^{\textrm{stat}} + \frac{f}{N} \boldsymbol{1} \right) \right] \end{aligned}$$

where *N* is the number of nodes. The specific probability values can be viewed as a centrality measure of nodes:

$$\begin{aligned} \textrm{RWC} (i) = (\boldsymbol{p}^{\textrm{stat}})_i. \end{aligned}$$

On global level, we used the following hierarchy measures:

*Flow hierarchy (FH)* is defined as the fraction of links which do not participate in any cycles, i.e.,

$$\begin{aligned} \textrm{FH} = \frac{1}{L} \cdot \sum _{i \in E} e_i \end{aligned}$$

where *E* denotes the set of links, *L* is the number of links (i.e., *L = |E|*), and $e_i = 0$ if link *i* is in a cycle while $e_i = 1$ if it is not, in the case of an unweighted network. In the case of a weighted network, $e_i$ is equal to the weight of link *i* if it is not in a cycle, and $L = \sum _{i \in E} e_i$. This quantity measures the extent to which a network’s structure resembles a directed acyclic graph, where information or control flows unidirectionally without cycles^54^.

*Global reaching centrality (GRC)* can be defined as the heterogeneous distribution of an m-reach centrality. Using *m = 2*, if $\textrm{2RC} (i)$ is the 2-reach centrality of node *i* and $\textrm{2RC}^{\max }$ is the highest centrality value, then following the formalism of Mones et al.^35^, GRC is defined as

$$\begin{aligned} \textrm{GRC} = \frac{1}{N - 1} \cdot \sum _{i \in V} \left[ \textrm{2RC}^{\max } - \textrm{2RC} (i) \right] \end{aligned}$$

where *V* is the set of nodes and *N* is the number of nodes (i.e., *N = |V|*).

*Random walk hierarchy (RWH)* is based on a similar idea as GRC, but using the random walk centrality. Since the distribution $\textrm{RWC}$ values characterises how important the specific nodes in terms of information flow, its relative width

$$\begin{aligned} \textrm{RWH} = \sigma (\boldsymbol{p}^{\textrm{stat}})\,/\,\mu (\boldsymbol{p}^{\textrm{stat}}) \end{aligned}$$

measures the degree of hierarchy of the network itself^37^.

### Ranking methods

Player ability is also an abstract concept, and quantifying it can be done in many different ways. The situation is also complicated by the fact that an official ranking method of a sport is often not applicable to any other sport, and hence is incomparable. In this paper, we used three universal ranking methods to measure player strengths, one of which was the official one, so the comparability was handled through its officialness.

*Sports federation score (SFS)* is the score calculated by the governing body of the sport, which is used for determining the“official ranking”of players. Both fencing and tennis have their point system to reward player performance, and a real-time updating list where the current ranking can be followed^75^–^77^. Due to the officialness of this score, players take this one the most seriously and prioritise to maximise it.

*Elo rating system (ELO)* is calculated by the method suggested by Arpad Elo originally for measuring a player’s performance in chess^62,63^. Since Elo scores are not tracked officially in most sports, we needed to calculate it for the data according to Elo’s method: all players started with a score of $s_0$, and we went through the matches chronologically; when a player with score $s_1$ won against an opponent with score $s_2$, we updated their scores as $s_1 \rightarrow s_1 + K \cdot (1 - E_1)$ and $s_2 \rightarrow s_2 - K \cdot (1 - E_1)$, where $E_1$ is the prior probability of this outcome: $E_1 = (1 + 10^{(s_2 - s_1) / S})^{-1}$. The parameters $s_0$, *K* and *S* were chosen to be $s_0 = 1500$, *K = 32* and *S = 400* in accordance with the standard rating formula in the official USCF (United States Chess Federation) rating system^68^.

*Reversed PageRank (RPR)* is calculated from the networks of matches, based on the concept introduced in the Results: We calculated the PageRank centrality of the nodes as if we reversed the direction of links (i.e., as if they pointed from the loser towards the winner). PageRank ranks nodes (originally web pages on the internet) based on the importance of the nodes linking to them^71^. Nodes with more incoming links from important nodes receive higher PageRank scores, indicating their greater significance in the network. Since in the reversed case the role of the link directions is the opposite, in our case nodes with links towards important nodes received high scores i.e., players who defeated other players with high scores. The exact scores are calculated by solving the following eigenvalue problem:

$$\begin{aligned} \textrm{RPR} (i) = \frac{1 - d}{N} + d \cdot \sum _{j \in M (i)} \frac{\textrm{RPR} (j)}{L (j)} \end{aligned}$$

where *N* is the number of all nodes in the network, *M*(*i*) is the set of nodes to which *i* is linked, *L*(*j*) is the number of inbound links on *j*, and *d* is and arbitrary parameter which we chosen to be *d = 0.85*. The meaning of damping factor *d* in the context of sports networks can be interpreted as a parameter of the fleeting of *prestige*: *1 - d* is the rate of decrease with which a player loses reputation over time without gaining a new victory.

One can see that all of the above scores change over time, thus making it possible for them to reflect the changes in the relationship of player strengths. SFS is rolling both in the fencing and tennis categories with a one-year time window. This means the score of a player is counted from the results achieved in the last 12 months, and when a match becomes older than one year, the points gained by participating in it cease (and possibly replaced by the event of the actual year). The dynamic behaviour of Elo scores is encoded into its calculating method: each match updates the current score of the players, so the newest match always has the largest effect on the scores. Since RPR is calculated from a time-dependent network, its value changes in the same way as the network itself, similar to the cases of local hierarchy centralities.

### Network randomisation methods

To compare the values of hierarchy measures in real networks, we randomised the original networks and used them as the basis for comparison. We used two randomisation models, which are intended to remove a significant amount of the statistical correlations within the network while preserving its most basic features, allowing the new network to be comparable to the original one. Thus, since the new networks are not completely random but generated from the original one to some extent, their hierarchical structure might also depend on the original structure. This must be taken into account during the comparison, and the interpretation of the results must be assessed with due caution, as we explained in the Results. The two randomisation methods are the following:

*Erdős–Rényi model* keeps the number of nodes and links of the original network while rewriting the links, i.e. both source and target nodes are chosen randomly for each link. This means all nodes are equivalent, their degree distribution follows a binomial distribution, and most of the node features have a typical value around which the individual values fluctuate. Thus, an Erdős–Rényi graph is typically not hierarchical and can serve as a standard of the comparison for the first try.^78^

*Configuration model* also keeps the number of nodes and links but also reserves the degree distribution of nodes. This is achieved instead of randomly assigning source and target nodes to links, relinking them by swapping the targets of two randomly chosen links at once and repeating this swapping step many times. (In our case, for a network with *L* links, we applied 5*L* iterations, so each link was relinked 5 times on average.) During the swaps, the link weights move together with their links. Since the degree distribution is guaranteed to be the same as the one of the (supposedly) hierarchical network, there will certainly be some structural differences between nodes, which can easily appear through the value of the hierarchical measures. But this is not the result of the relationship of the nodes but their different state in the network.^79^

During our work, we generated $N_{\textrm{rand}} = 10$ randomised networks of both kinds of randomisation for each year in each sports category, which can estimate the typical sizes of the distribution within roughly 24% of relative uncertainty. In Fig. 2, the distribution of randomised values was estimated based on this sample.

### Making predictions using score differences

In the Results we calculated the probability of outcomes for each match having sufficient prior information using formula (2) which contains two parameters *α* and *β*. For optimal prediction, we need to determine their optimal value. Since the temporal character of the data is essential in our problem, we decided to handle the optimal parameter values as temporal variables as well. This is also in accordance with the real-world situation, where bookmakers and gamblers try to make predictions knowing the latest results and events.

**Fig. 8:**
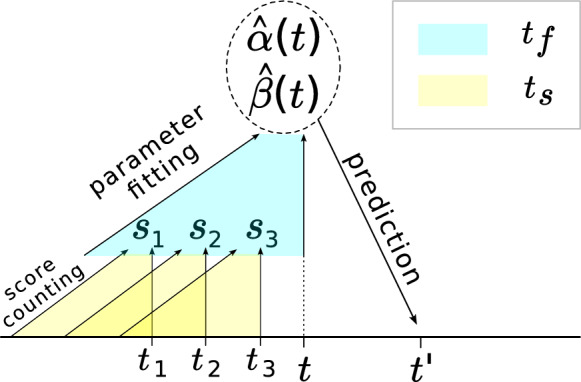
**Illustration of the fitting process.** We use matches from the score-counting period $t_\textrm{s}$ (yellow) before certain times ($t_1$, $t_2$, $t_3$) to calculate prediction scores ($s_1$, $s_2$, $s_3$) then, we use such scores from the fitting period $t_\textrm{f}$ (blue) before time *t* to find the optimal parameter values $\hat{\alpha } (t)$ and $\hat{\beta } (t)$ which gives us the best trial prediction at *t*. Finally, in the possession of the fitted parameters, we can predict future matches at *t' > t.*.

For fitting on formula (2) and finding the optimal values $\hat{\alpha } (t)$ and $\hat{\beta } (t)$ at time *t*, we need a certain number of finished matches from before *t* based on which we calculate the scores, and the trial probabilities for matches at *t*. If we already know the results of matches at *t*, we can determine the best parameter values which result the best trial probabilities by using the method of least squares. To make predictions for times *t' > t*, we can use these parameter values. Hence, for making a real prediction for a specific sports event, we need to know the results at least from a previous fitting period $t_{\textrm{f}}$ on which we fit the parameters. Also, to make trial predictions on matches in the fitting period, we need to know the results from a score-counting period $t_{\textrm{s}}$ before the fitting period, which we use for calculating prediction scores in trial predictions. This relationship between different sliding time windows is illustrated in Fig. 8.

For scores that are node centralities, the score-counting period is the period from which the matches are used to build the network, i.e. $t_{\textrm{s}} = t_{\textrm{nb}}$. For the results shown in Table 4, the length of both time windows were chosen to be $t_{\textrm{f}} = 1\,\textrm{year}$ and $t_{\textrm{s}} = 1\,\textrm{year}$. Our time resolution was also 1 year, i.e. the scores at the end of the previous year were taken as the actual scores of players through a whole calendar year. (E.g., Novak Djokovic had 11,360 ATP points on December 31, 2014, so we used this value for making predictions for all the matches Djokovic played in 2015. The prediction parameters used in 2015 was $\hat{\alpha }_{SFS} (2014) = 0.17$ and $\hat{\beta }_{SFS} (2014) = 1.3 \cdot 10^{-3}$, which were obtained by fitting formula (2) on match results from 2014.)

Once, we calculated the outcome probabilities for the matches with a certain score, we compared them with the real results. If we estimated probability $p_m$ for the real outcome of match *m*, the larger $p_m$ value is, the more accurate our method is. We measured the collective accuracy for all *M* matches of the method by three quantities:

*Fraction of mistakes (FM)* is the number of matches where we predicted lower probability for the winner to win divided by the number of all matches. Basically, if we have had to guess for each match which player would win, this is the fraction of times we would have been wrong. Formally,

$$\begin{aligned} \text {FM} = \frac{1}{M} \cdot \# \left\{ m: p_m < 0.5 \right\} . \end{aligned}$$

(If the estimated probability was 0.5 for both players, we counted it as a half mistake, since in this case we would have to choose between the two players randomly.) Optimization for this quantity can provide us to be the best prediction reliability. For an absolute random prediction, this quantity would be 0.5.

*Average squared error (SE)*, i.e., the Brier score is the squared difference between the estimated probability and 1, averaged over all matches, i.e.

$$\begin{aligned} \text {SE} = \frac{1}{M} \cdot \sum _m (1 - p_m)^2. \end{aligned}$$

This is the cost function of the fitting using the method of least squares, so optimization for this quantity can be considered as the maximum likelihood strategy. For an absolute random prediction, this would be 0.25.

*Average linear error (LE)* is the linear difference between the estimated probability and 1, averaged over all matches, i.e.

$$\begin{aligned} \text {LE} = \frac{1}{M} \cdot \sum _m (1 - p_m) \end{aligned}$$

Since the single difference of $(1 - p_m)$ is the expected value of making a wrong prediction for match *m*, the whole sum can be considered as the risk of betting on the more probable player at each match. Bookmakers and gamblers want to minimize this value, so optimization for this value yields us the best financial profit. For an absolute random prediction, this would be 0.5.

*Area under the ROC curve (AUC)* measures the entire two-dimensional area underneath the ROC curve, providing an aggregate measure of a binary classifier’s performance across all possible classification thresholds. Statistically, it represents the probability that the model will rank a randomly chosen positive instance higher than a randomly chosen negative one^80^. For an absolute random prediction, this would be 0.5.

### Cycle abundance and cycle enrichment

To quantify network hierarchy resulting from the organizational structure of the championships and the dominance relationship between players, we presented several method to measure how much more cycle is contained in the graph than it should be in some kinds of idealisation. In Fig. 2b, d, f, and h, we plotted the cycle abundance measured in two different ways, which are meant to quantify how many cycles the graphs contain normed to their size. The two formalisations of cycle abundance are: 

*Cycle density* is the number of 3-cycles divided by the number of all possible node-triads in the graph, i.e. $$\left( {\begin{array}{c}N\\ 3\end{array}}\right)$$ where *N* is the number of nodes. This is a simple size-normalised number of cycles.

$$\begin{aligned} \text {Cycle density} = \frac{\# \{\text {3-cycles}\}}{\left( {\begin{array}{c}N\\ 3\end{array}}\right) } \end{aligned}$$

*Cycle-FFL ratio* is the number of 3-cycles divided by the number of all weakly connected node-triads (i.e. the sum of 3-cycles and feedforward 3-loops) in the graph. The normalisation of this quantity is done not by just the size of the graph, but the clustering of nodes.

$$\begin{aligned} \text {Cycle-FFL ratio} = \frac{\# \{\text {3-cycles}\}}{\# \{\text {3-cycles}\} + \# \{\text {FFLs}\}} \end{aligned}$$

In Fig. 3, we plotted the cycle enrichment, which measures how many more cycles there are in the network than expected from the given distribution of player abilities. For this, we relinked the network in the following way: we went through all the matches represented by the network’s links and guessed the winner of each match by randomly drawing from the Bernoulli distribution given by the winning probability of the prediction process detailed in the previous subsection. This guessing did not take into account the real result of the match, only the calculated ranking scores and the fitted parameters which were gained from the previous match results. If the randomly drawn winner matched the actual winner, we left the link unchanged. However, if the randomly drawn winner was the other player, we reversed the direction of the link. Hence, in the new network, the number of 3-cycles could differ from the original one, while the weak connectivity of nodes remained the same. The ratio of the real number of 3-cycles to their number in the relinked network expresses how much more probable a cycle to form in real life than expected, and how much more enriched the real graph with cycles than the sampled one.

$$\begin{aligned} \text {Cycle enrichment} = \frac{\# \{\text {3-cycles in the real network}\}}{\# \{\text {3-cycles in the simulated network}\}} \end{aligned}$$

During our work, we generated $N_{\textrm{sim}} = 10$ simulated networks in each year in each sports category, which can estimate the typical sizes of the distribution within roughly 24% of relative uncertainty. In Fig. 3, the distribution of the simulated values was estimated based on this sample.

## Supplementary Information

Supplementary Information.

## Data Availability

We used datasets publicly available on the internet. Men’s and women’s tennis data was collected by Jeff Sackmann who made them downloadable from his corresponding GitHub repositories, https://github.com/JeffSackmann/tennis_atp , https://github.com/JeffSackmann/tennis_wta . All fencing data was downloaded from the official website of the International Fencing Federation, using the code of Anya Post-Michaelsen, https://github.com/amichaelsen/fie-fencing-dataset.
