# Supplementary material for: Hierarchy and ranking in fencing and tennis
Source: Sci Rep. 2026 May 31;16:24877. doi: 10.1038/s41598-026-53459-7 (PMC13457841; doi:10.1038/s41598-026-53459-7)
Supplement: Supplementary file 1 [file 41598_2026_53459_MOESM1_ESM.pdf]

# Hierarchy and ranking in fencing and tennis: Supplementary Information

**Bogdán Asztalos, Boldizsár Balázs, Gergely Palla, and Tamás Vicsek**

In this document, we present the results for each sports category, which could not be included in the main text. In Figures 2-7 of the main text, only the cases of men's tennis and men's sabre are showed, and conclusion is drawn based on them. Here, the analogous figures are collected for all studied sports, and the reader can see that they also support the claims of the paper.

## Hierarchy measures in the network of matches

In the left column of Figure 2 of the main text, we plotted the different hierarchy measures over time for the network of real matches, and the value of these measures for two types of network randomisation. There, we presented the results of men's tennis tournaments, and men's sabre tournaments. In this section, we show the analogous results of all the studied dataset in Figures S1-S8.

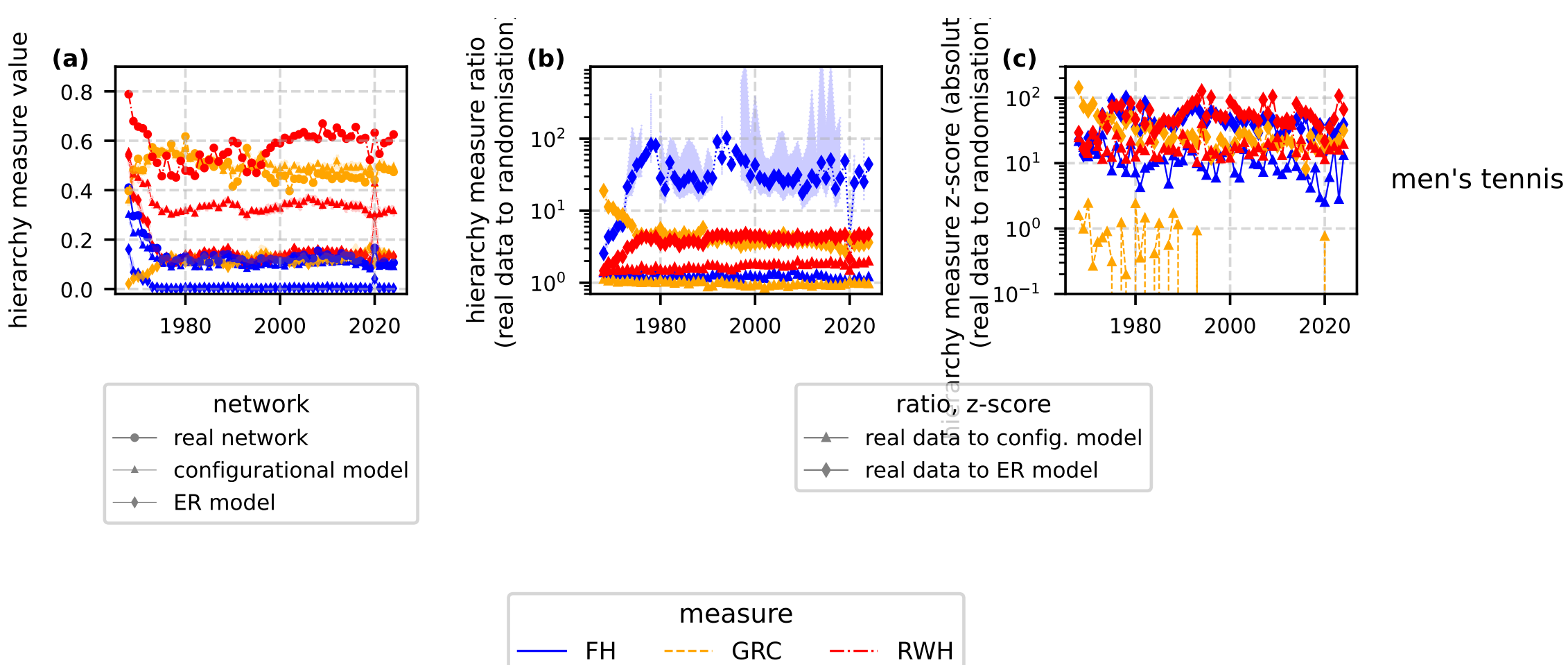


**Figure S1.** **Hierarchy measures in the network of men's tennis matches. (a)** The flow hierarchy (blue), the global reaching centrality based on 2-reach (orange), and the random walk hierarchy (red) over time, displaying both the measured value in the original networks (circles) and also the average value in randomised networks according to the configuration model (triangles) and the Erdős–Rényi model (diamonds). **(b)** The three hierarchy values measured in the real network shown in panel (a), divided by the values in the randomised networks according to the configuration model (triangles) and the Erdős–Rényi model (diamonds). **(c)** The three hierarchy values measured in the real network shown in panel (a), transformed as a z-score over the distribution of the values in the randomised networks according to the configuration model (triangles) and the Erdős–Rényi model (diamonds).

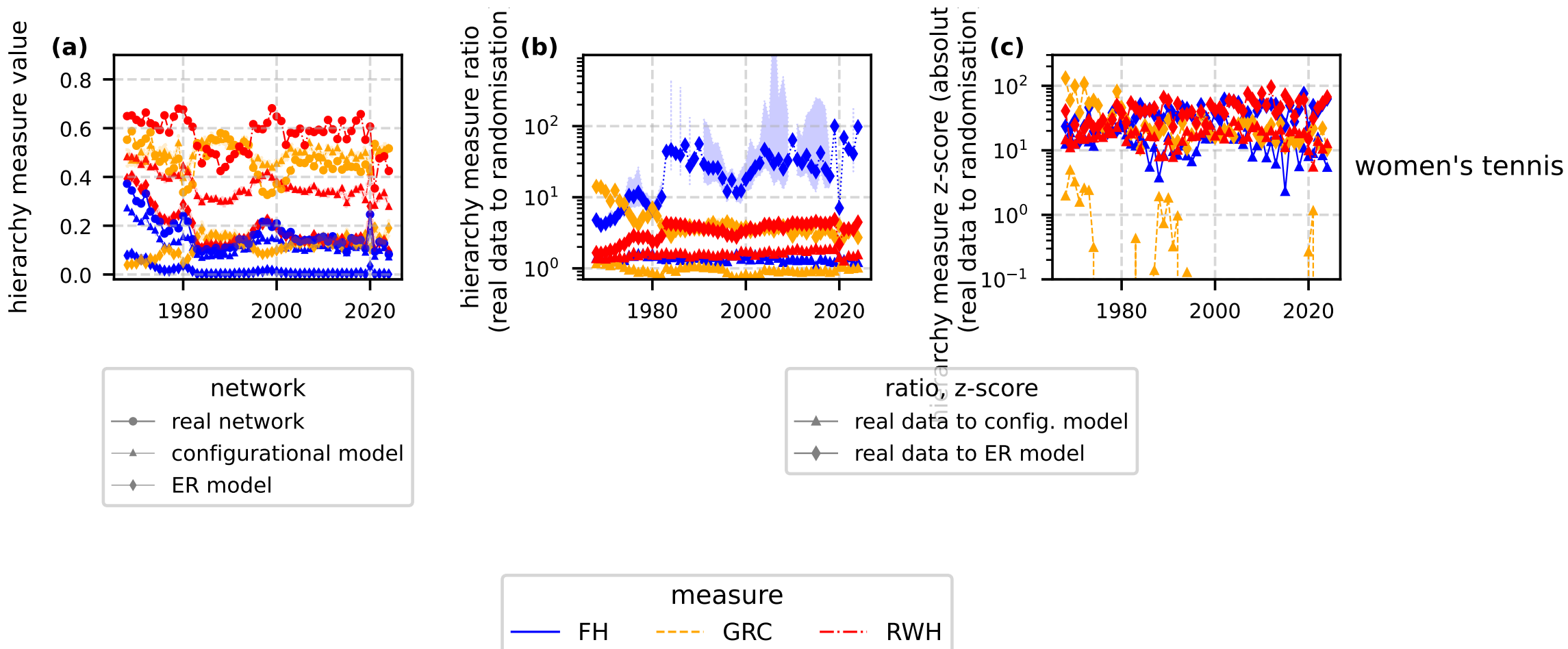


**Figure S2. Hierarchy measures in the network of women's tennis matches. (a)** The flow hierarchy (blue), the global reaching centrality based on 2-reach (orange), and the random walk hierarchy (red) over time, displaying both the measured value in the original networks (circles) and also the average value in randomised networks according to the configuration model (triangles) and the Erdős–Rényi model (diamonds). **(b)** The three hierarchy values measured in the real network shown in panel (a), divided by the values in the randomised networks according to the configuration model (triangles) and the Erdős–Rényi model (diamonds). **(c)** The three hierarchy values measured in the real network shown in panel (a), transformed as a z-score over the distribution of the values in the randomised networks according to the configuration model (triangles) and the Erdős–Rényi model (diamonds).

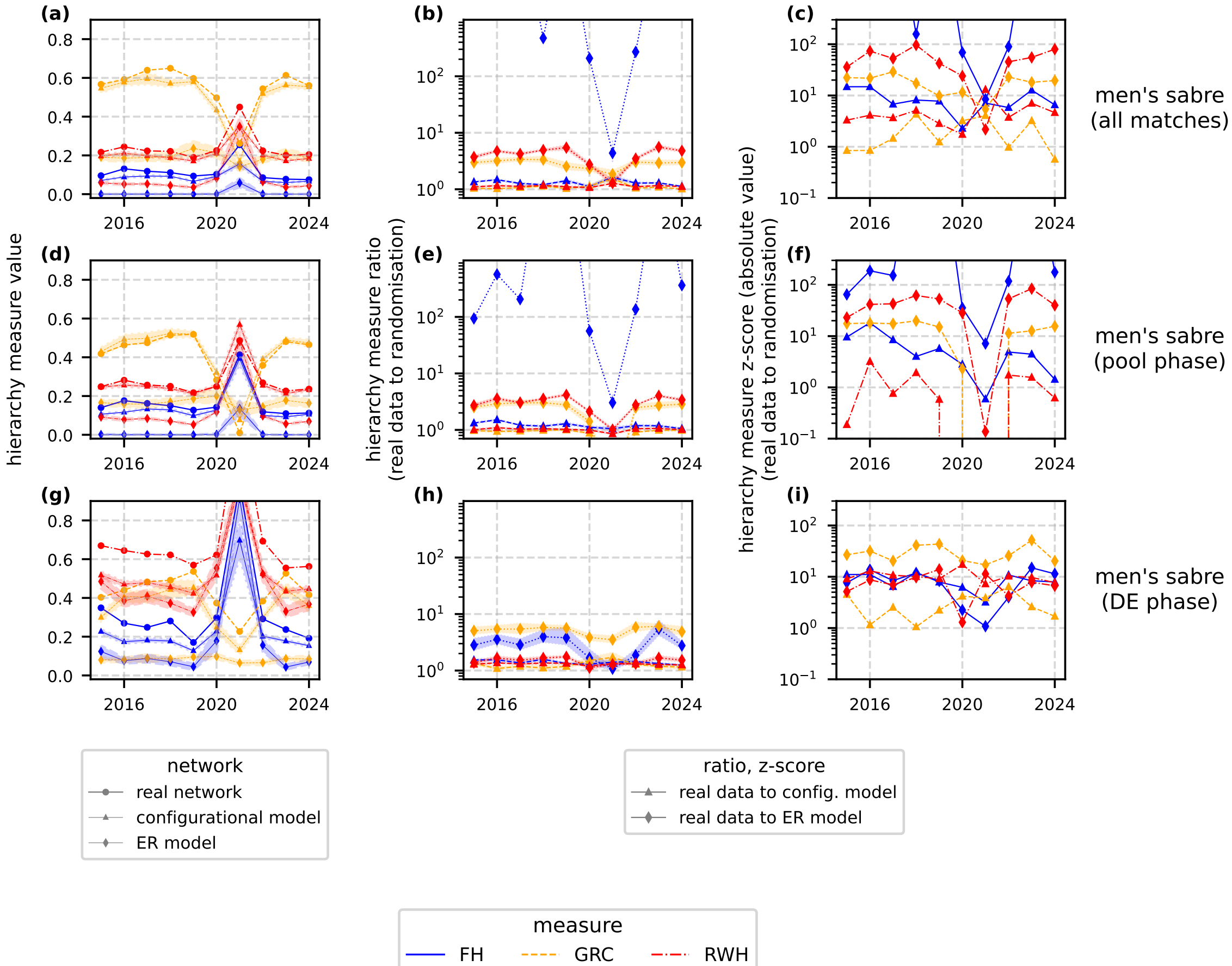


**Figure S3. Hierarchy measures in the network of men's sabre matches. (a, d, g)** The flow hierarchy (blue), the global reaching centrality based on 2-reach (orange), and the random walk hierarchy (red) over time, displaying both the measured value in the original networks (circles) and also the average value in randomised networks according to the configuration model (triangles) and the Erdős–Rényi model (diamonds). **(b, e, h)** The three hierarchy values measured in the real network shown in panel (a, d, g), divided by the values in the randomised networks according to the configuration model (triangles) and the Erdős–Rényi model (diamonds). **(c, f, i)** The three hierarchy values measured in the real network shown in panel (a, d, g), transformed as a z-score over the distribution of the values in the randomised networks according to the configuration model (triangles) and the Erdős–Rényi model (diamonds).

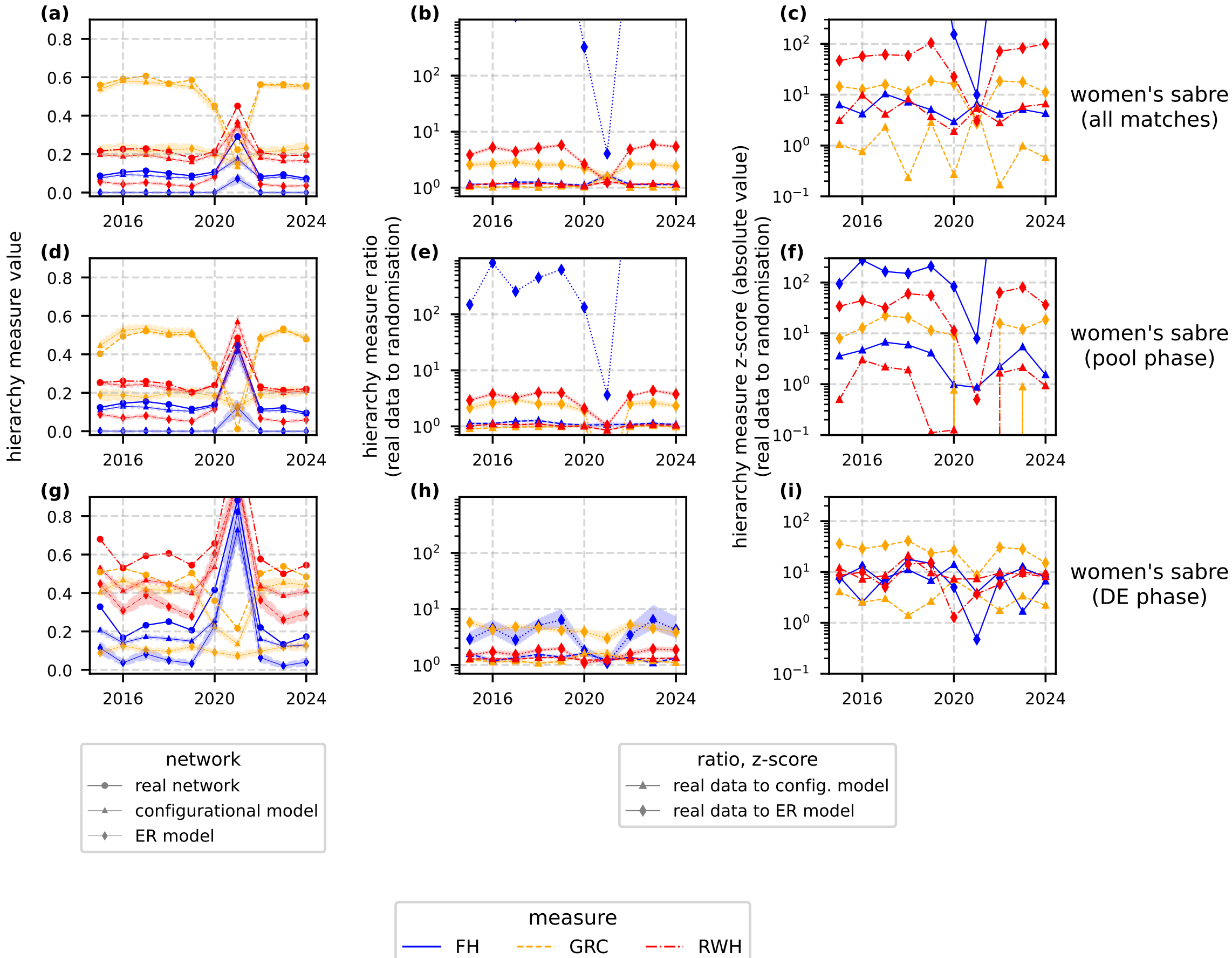


**Figure S4. Hierarchy measures in the network of women's sabre matches. (a, d, g)** The flow hierarchy (blue), the global reaching centrality based on 2-reach (orange), and the random walk hierarchy (red) over time, displaying both the measured value in the original networks (circles) and also the average value in randomised networks according to the configuration model (triangles) and the Erdős–Rényi model (diamonds). **(b, e, h)** The three hierarchy values measured in the real network shown in panel (a, d, g), divided by the values in the randomised networks according to the configuration model (triangles) and the Erdős–Rényi model (diamonds). **(c, f, i)** The three hierarchy values measured in the real network shown in panel (a, d, g), transformed as a z-score over the distribution of the values in the randomised networks according to the configuration model (triangles) and the Erdős–Rényi model (diamonds).

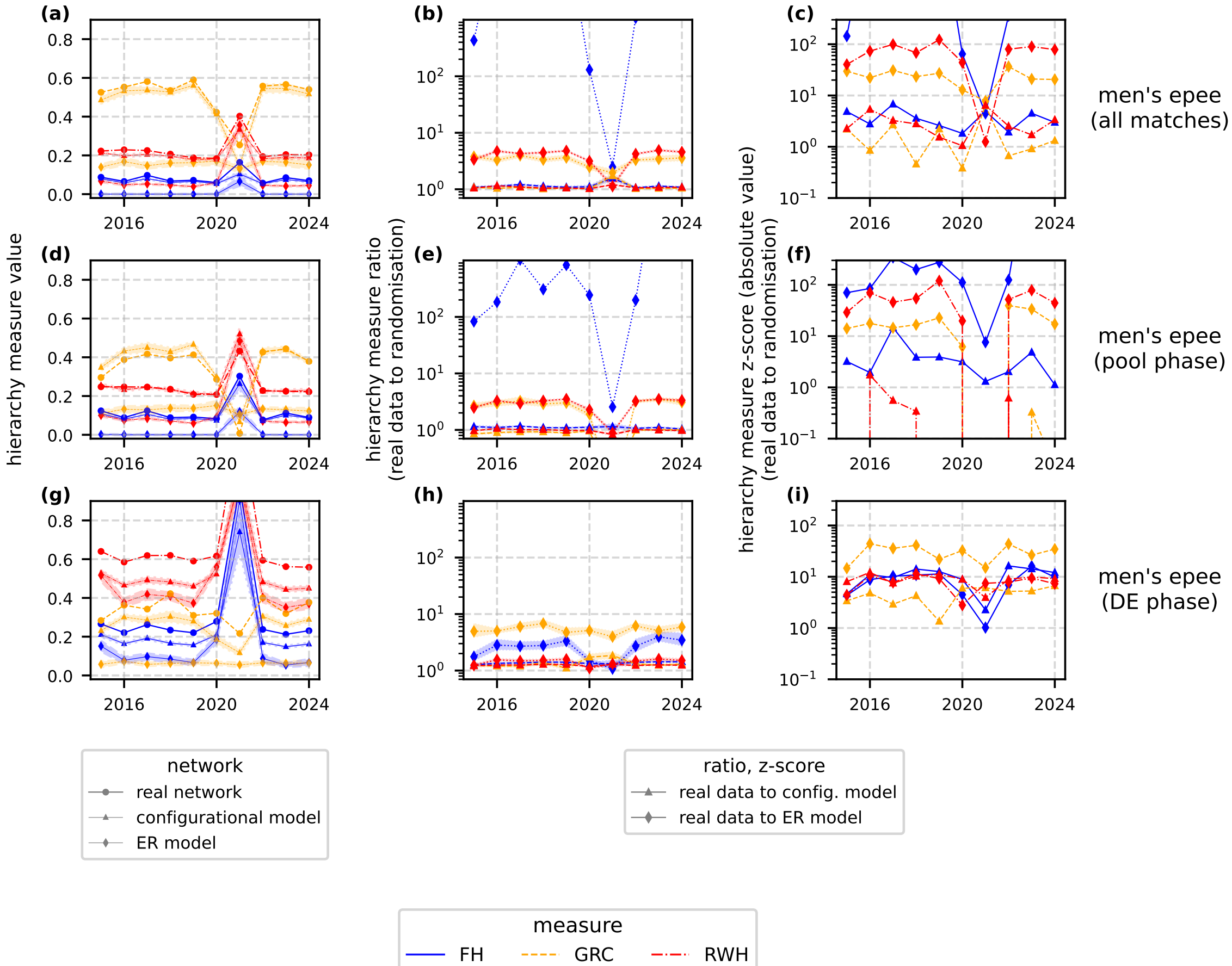


**Figure S5. Hierarchy measures in the network of men's épée matches. (a, d, g)** The flow hierarchy (blue), the global reaching centrality based on 2-reach (orange), and the random walk hierarchy (red) over time, displaying both the measured value in the original networks (circles) and also the average value in randomised networks according to the configuration model (triangles) and the Erdős–Rényi model (diamonds). **(b, e, h)** The three hierarchy values measured in the real network shown in panel (a, d, g), divided by the values in the randomised networks according to the configuration model (triangles) and the Erdős–Rényi model (diamonds). **(c, f, i)** The three hierarchy values measured in the real network shown in panel (a, d, g), transformed as a z-score over the distribution of the values in the randomised networks according to the configuration model (triangles) and the Erdős–Rényi model (diamonds).

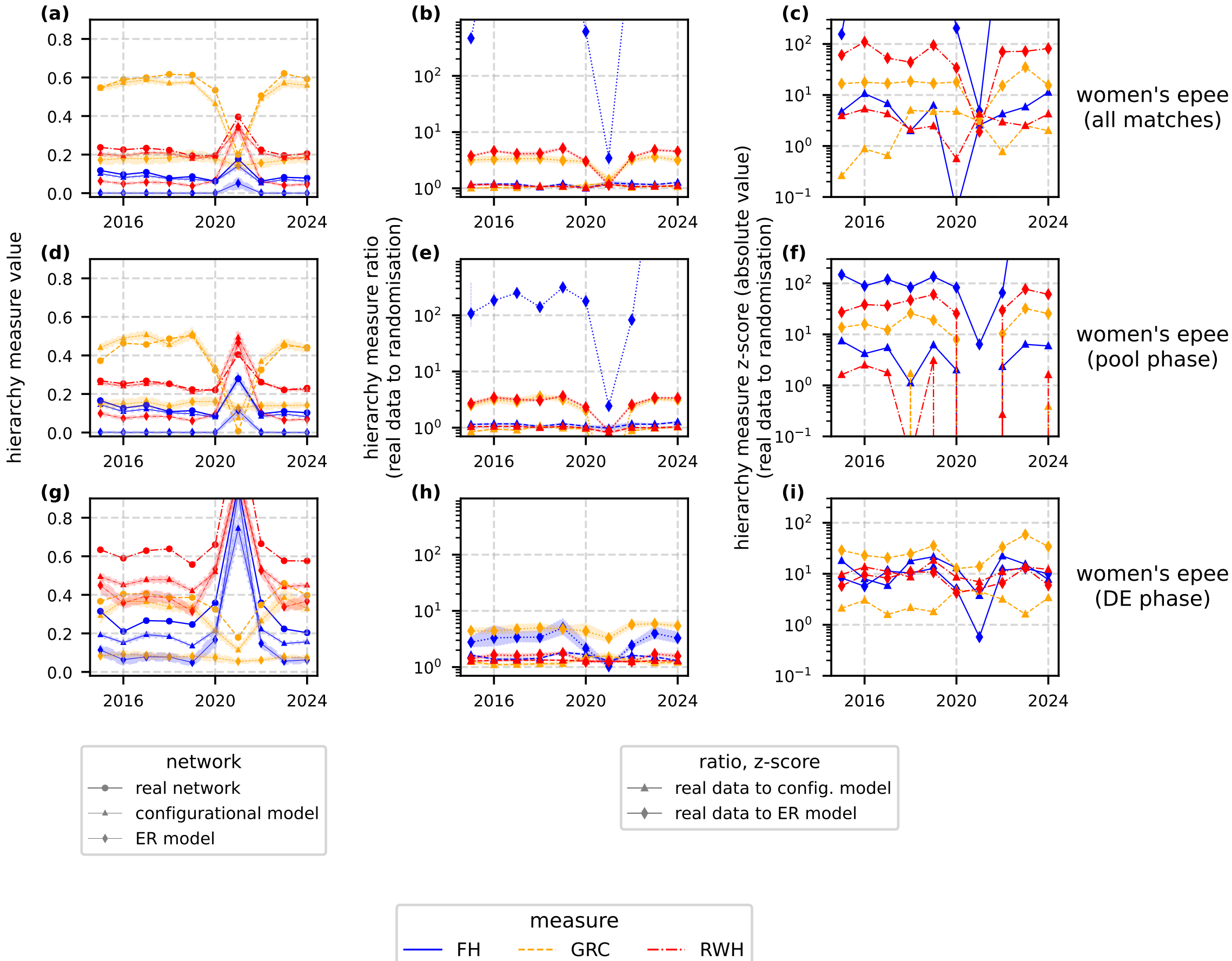


**Figure S6.** **Hierarchy measures in the network of women's épée matches. (a, d, g)** The flow hierarchy (blue), the global reaching centrality based on 2-reach (orange), and the random walk hierarchy (red) over time, displaying both the measured value in the original networks (circles) and also the average value in randomised networks according to the configuration model (triangles) and the Erdős–Rényi model (diamonds). **(b, e, h)** The three hierarchy values measured in the real network shown in panel (a, d, g), divided by the values in the randomised networks according to the configuration model (triangles) and the Erdős–Rényi model (diamonds). **(c, f, i)** The three hierarchy values measured in the real network shown in panel (a, d, g), transformed as a z-score over the distribution of the values in the randomised networks according to the configuration model (triangles) and the Erdős–Rényi model (diamonds).

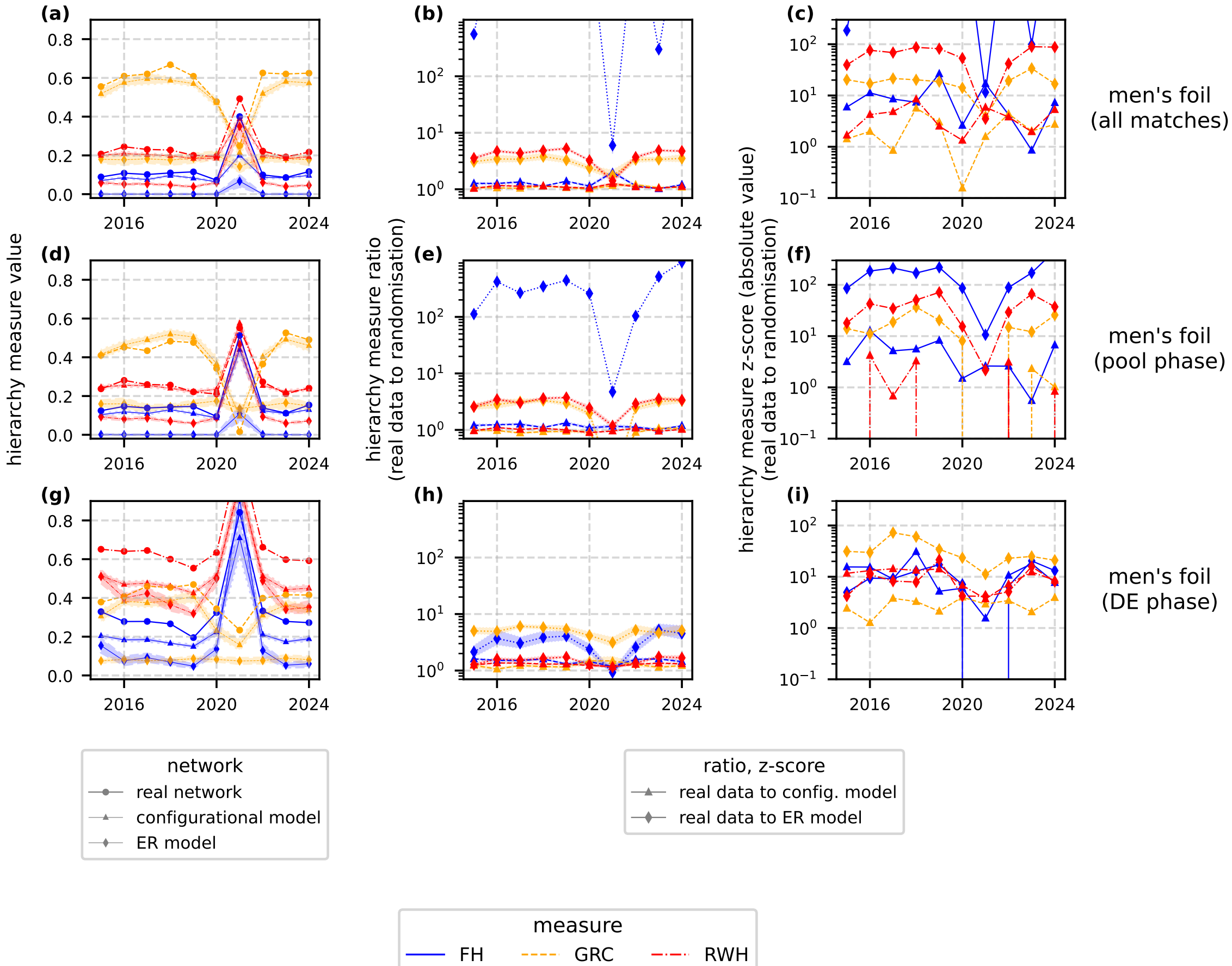


**Figure S7. Hierarchy measures in the network of men's foil matches. (a, d, g)** The flow hierarchy (blue), the global reaching centrality based on 2-reach (orange), and the random walk hierarchy (red) over time, displaying both the measured value in the original networks (circles) and also the average value in randomised networks according to the configuration model (triangles) and the Erdős–Rényi model (diamonds). **(b, e, h)** The three hierarchy values measured in the real network shown in panel (a, d, g), divided by the values in the randomised networks according to the configuration model (triangles) and the Erdős–Rényi model (diamonds). **(c, f, i)** The three hierarchy values measured in the real network shown in panel (a, d, g), transformed as a z-score over the distribution of the values in the randomised networks according to the configuration model (triangles) and the Erdős–Rényi model (diamonds).

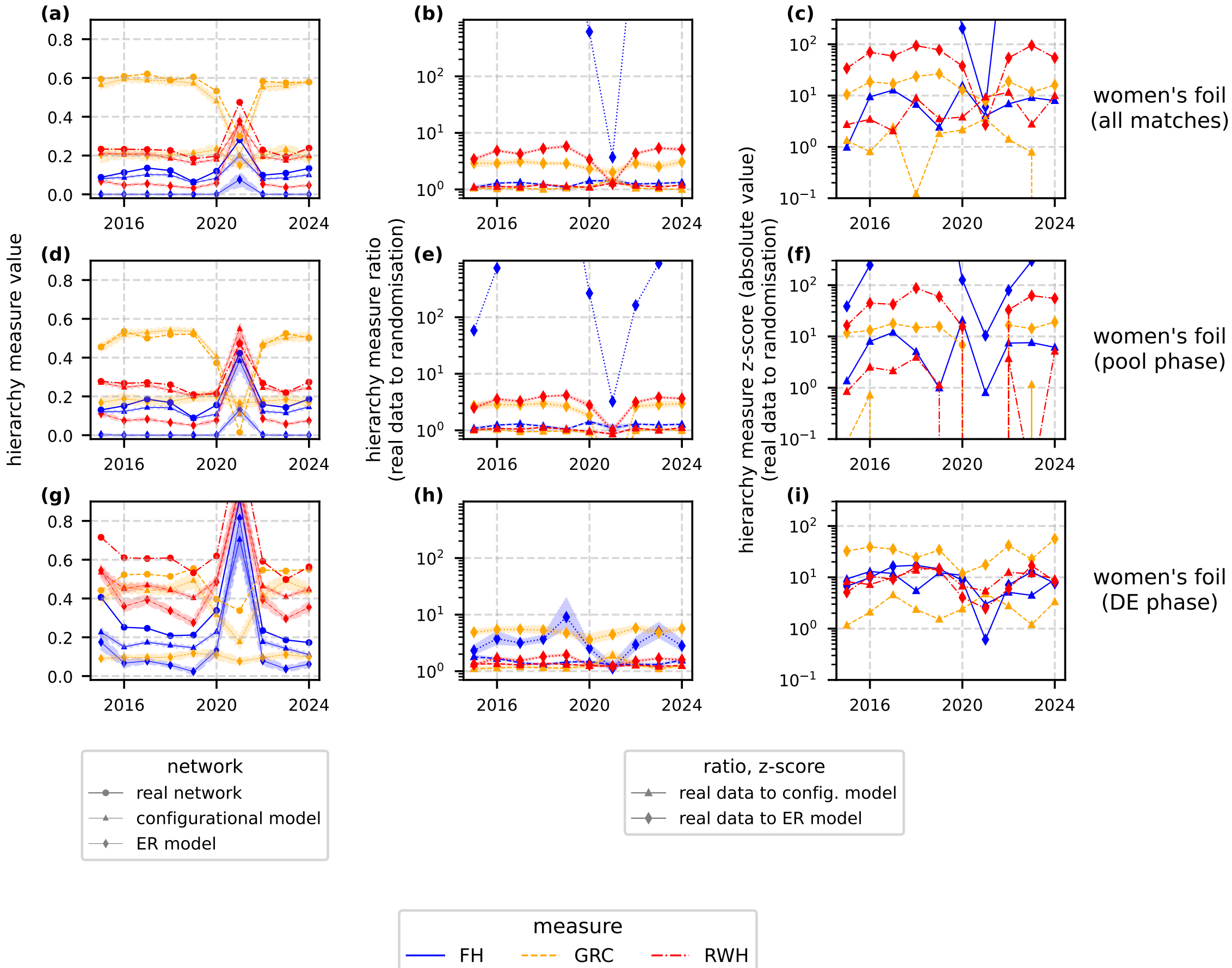


**Figure S8. Hierarchy measures in the network of women's foil matches. (a, d, g)** The flow hierarchy (blue), the global reaching centrality based on 2-reach (orange), and the random walk hierarchy (red) over time, displaying both the measured value in the original networks (circles) and also the average value in randomised networks according to the configuration model (triangles) and the Erdős–Rényi model (diamonds). **(b, e, h)** The three hierarchy values measured in the real network shown in panel (a, d, g), divided by the values in the randomised networks according to the configuration model (triangles) and the Erdős–Rényi model (diamonds). **(c, f, i)** The three hierarchy values measured in the real network shown in panel (a, d, g), transformed as a z-score over the distribution of the values in the randomised networks according to the configuration model (triangles) and the Erdős–Rényi model (diamonds).

## 3-cycle abundance in the network of matches

In the right column of Figure 2 of the main text, we plotted two ways of measure the cycle abundance over time for the network of real matches, and the value of these measures for two types of network randomisation. These quantities measured how many cycles are there in the network relative to the size of the network. There, we presented the results of men's tennis tournaments, and men's sabre tournaments. In this section, we show the analogous results of all the studied dataset in Figures S9-S16.

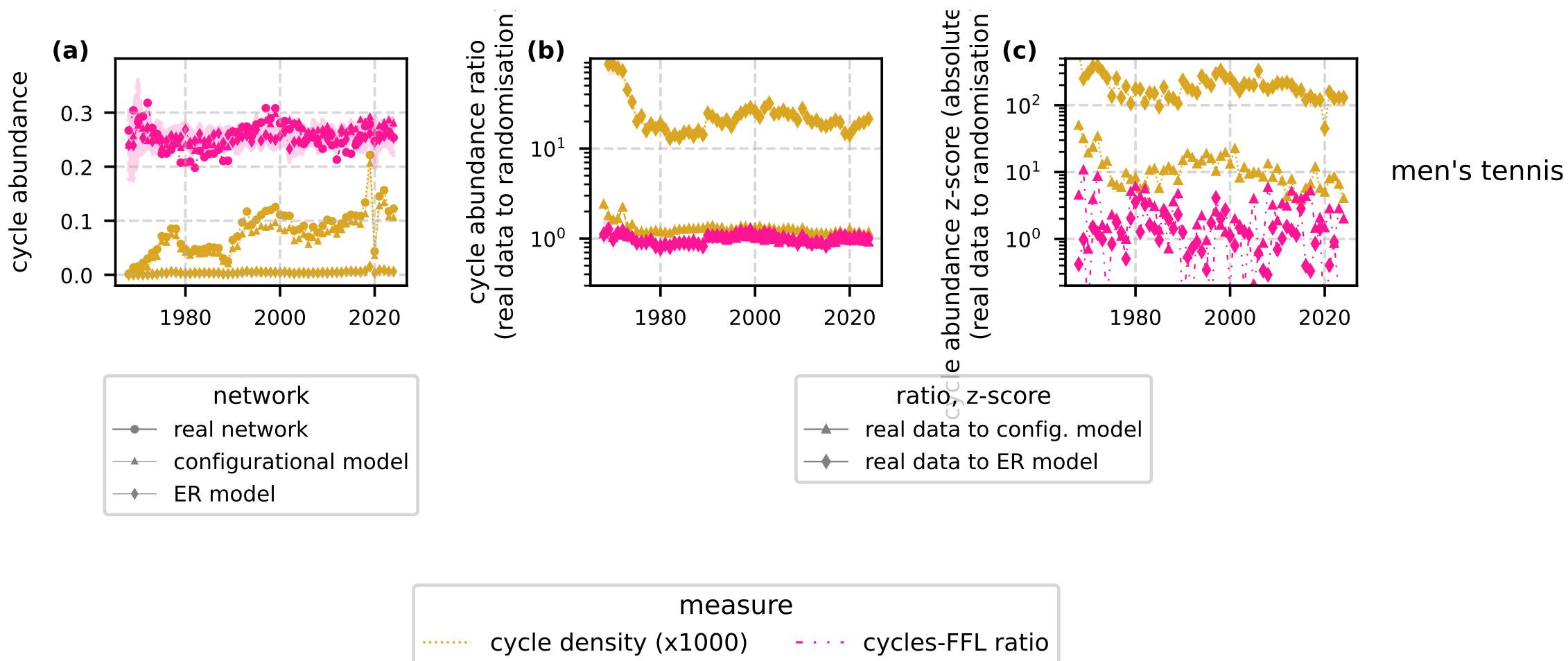


**Figure S9.** **3-cycle abundance in the network of men's tennis matches.** **(a)** The number of 3-cycles in the graph normed by the number all node triads (yellow), and its ratio to the number of feedforward 3-loops (FFLs) (pink) over time, displaying both the measured value in the original networks (circles), and also the average value in randomised networks according to the configuration model (triangles) and the Erdős–Rényi model (diamonds). The shaded regions around the averages indicate the standard deviation. **(b)** The two abundance values measured in the real network shown in panel (a), divided by the values in the randomised networks according to the configuration model (triangles) and the Erdős–Rényi model (diamonds). **(c)** The two abundance values measured in the real network shown in panel (a), transformed as a z-score over the distribution of the values in the randomised networks according to the configuration model (triangles) and the Erdős–Rényi model (diamonds).

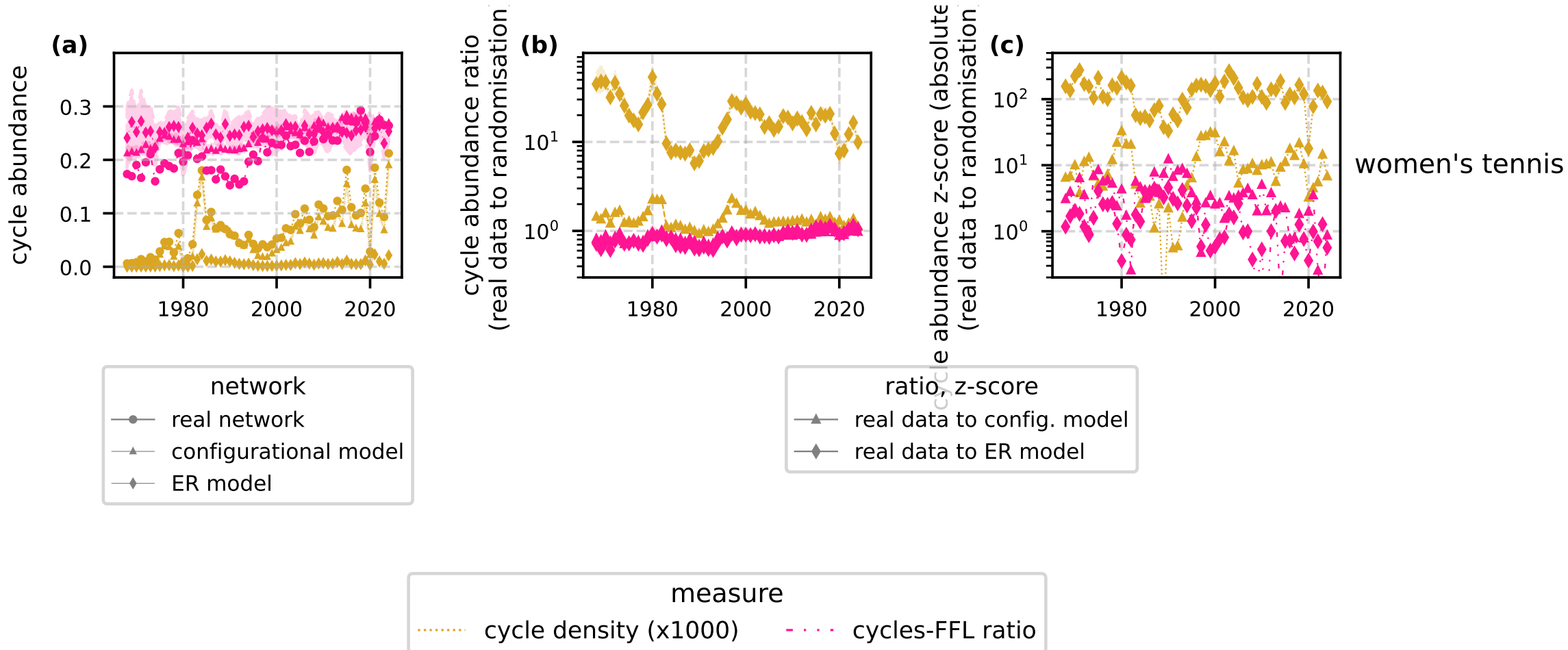


**Figure S10. 3-cycle abundance in the network of women's tennis matches. (a)** The number of 3-cycles in the graph normed by the number all node triads (yellow), and its ratio to the number of feedforward 3-loops (FFLs) (pink) over time, displaying both the measured value in the original networks (circles), and also the average value in randomised networks according to the configuration model (triangles) and the Erdős–Rényi model (diamonds). The shaded regions around the averages indicate the standard deviation. **(b)** The two abundance values measured in the real network shown in panel (a), divided by the values in the randomised networks according to the configuration model (triangles) and the Erdős–Rényi model (diamonds). **(c)** The two abundance values measured in the real network shown in panel (a), transformed as a z-score over the distribution of the values in the randomised networks according to the configuration model (triangles) and the Erdős–Rényi model (diamonds).

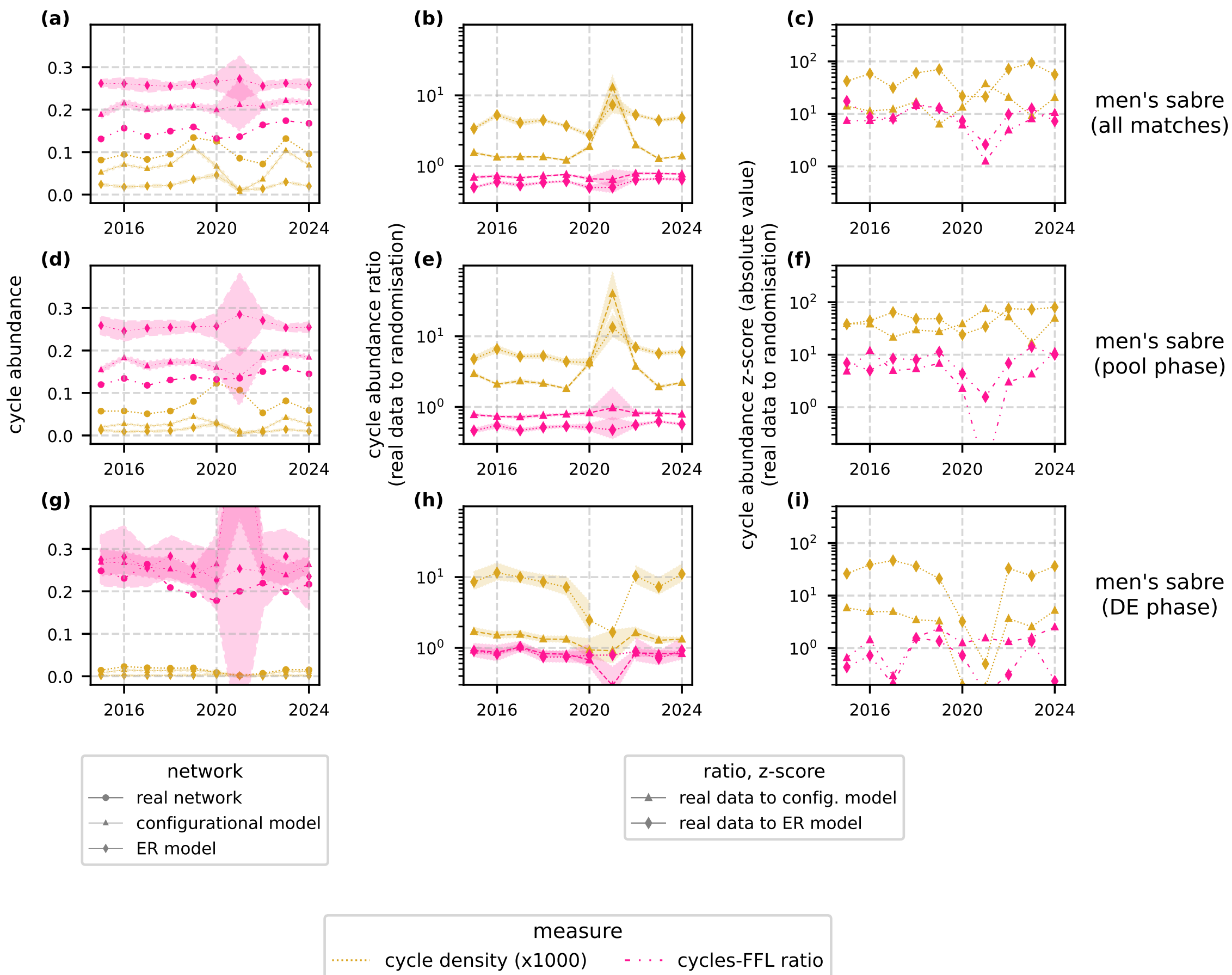


**Figure S11.** **3-cycle abundance in the network of men's sabre matches. (a, d, g)** The number of 3-cycles in the graph normed by the number all node triads (yellow), and its ratio to the number of feedforward 3-loops (FFLs) (pink) over time, displaying both the measured value in the original networks (circles), and also the average value in randomised networks according to the configuration model (triangles) and the Erdős–Rényi model (diamonds). The shaded regions around the averages indicate the standard deviation. **(b, e, h)** The two abundance values measured in the real network shown in panel (a, d, g), divided by the values in the randomised networks according to the configuration model (triangles) and the Erdős–Rényi model (diamonds). **(c, f, i)** The two abundance values measured in the real network shown in panel (a, d, g), transformed as a z-score over the distribution of the values in the randomised networks according to the configuration model (triangles) and the Erdős–Rényi model (diamonds).

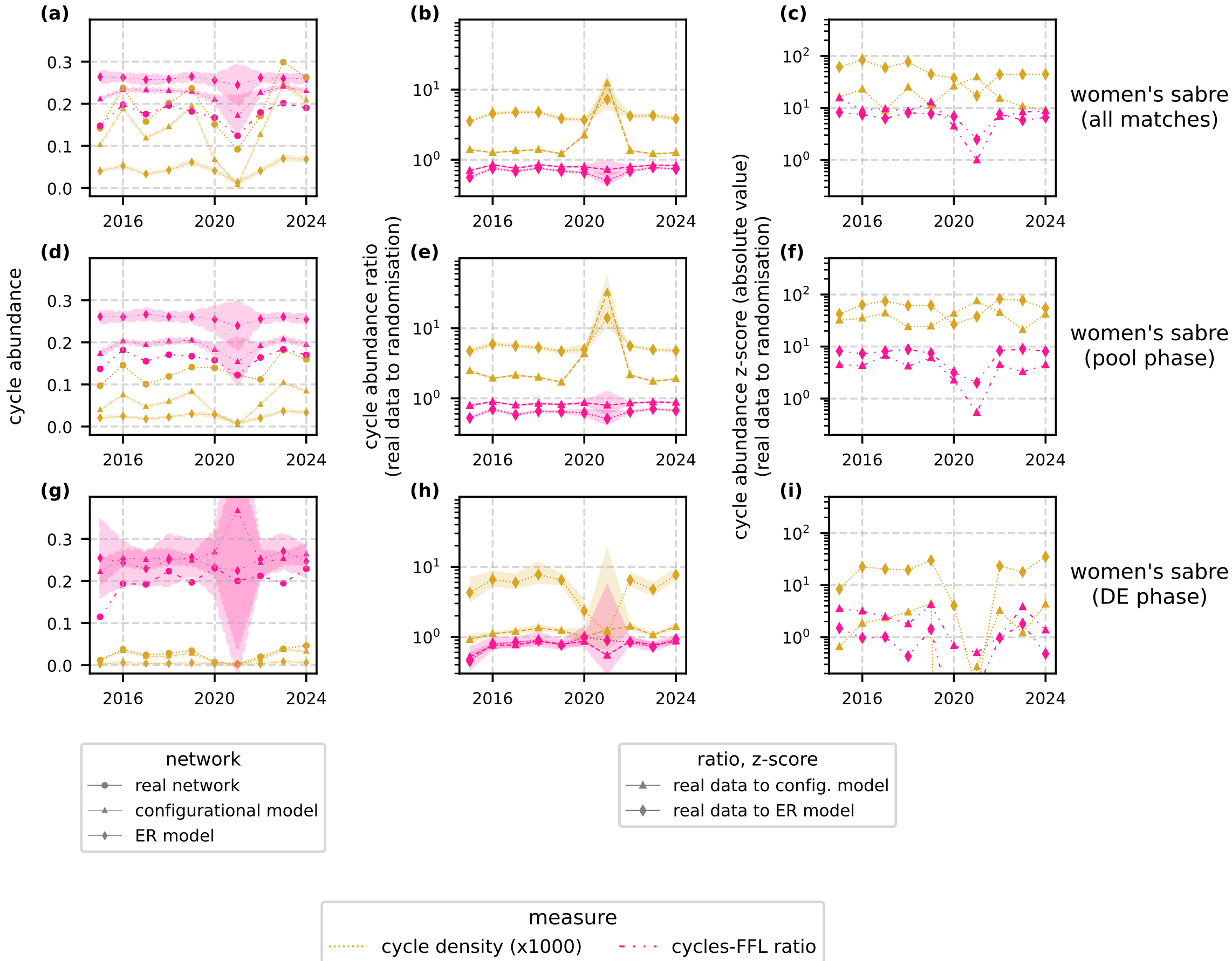


**Figure S12. 3-cycle abundance in the network of women's sabre matches.** **(a, d, g)** The number of 3-cycles in the graph normed by the number all node triads (yellow), and its ratio to the number of feedforward 3-loops (FFLs) (pink) over time, displaying both the measured value in the original networks (circles), and also the average value in randomised networks according to the configuration model (triangles) and the Erdős–Rényi model (diamonds). The shaded regions around the averages indicate the standard deviation. **(b, e, h)** The two abundance values measured in the real network shown in panel (a, d, g), divided by the values in the randomised networks according to the configuration model (triangles) and the Erdős–Rényi model (diamonds). **(c, f, i)** The two abundance values measured in the real network shown in panel (a, d, g), transformed as a z-score over the distribution of the values in the randomised networks according to the configuration model (triangles) and the Erdős–Rényi model (diamonds).

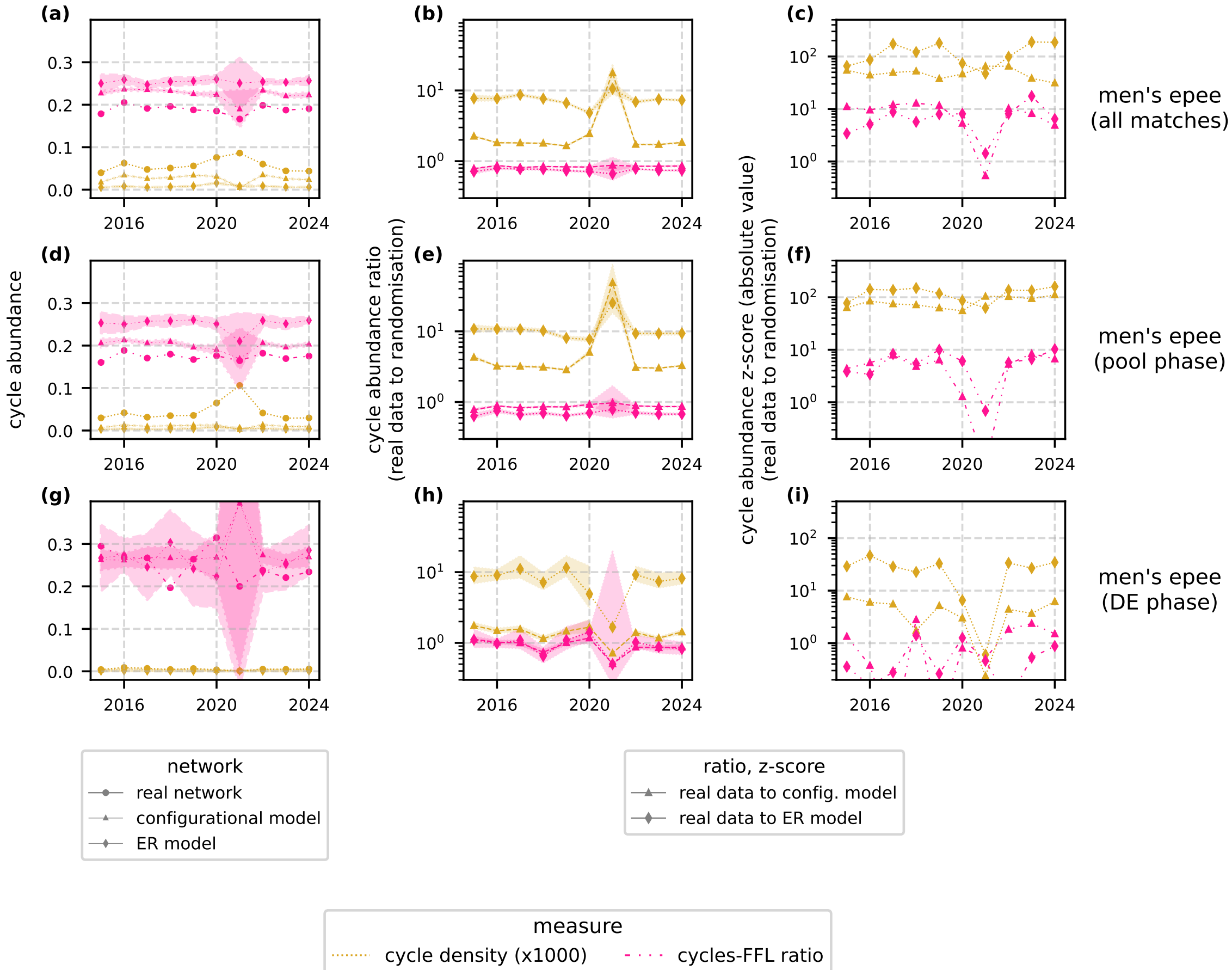


**Figure S13.** **3-cycle abundance in the network of men's épée matches. (a, d, g)** The number of 3-cycles in the graph normed by the number all node triads (yellow), and its ratio to the number of feedforward 3-loops (FFLs) (pink) over time, displaying both the measured value in the original networks (circles), and also the average value in randomised networks according to the configuration model (triangles) and the Erdős–Rényi model (diamonds). The shaded regions around the averages indicate the standard deviation. **(b, e, h)** The two abundance values measured in the real network shown in panel (a, d, g), divided by the values in the randomised networks according to the configuration model (triangles) and the Erdős–Rényi model (diamonds). **(c, f, i)** The two abundance values measured in the real network shown in panel (a, d, g), transformed as a z-score over the distribution of the values in the randomised networks according to the configuration model (triangles) and the Erdős–Rényi model (diamonds).

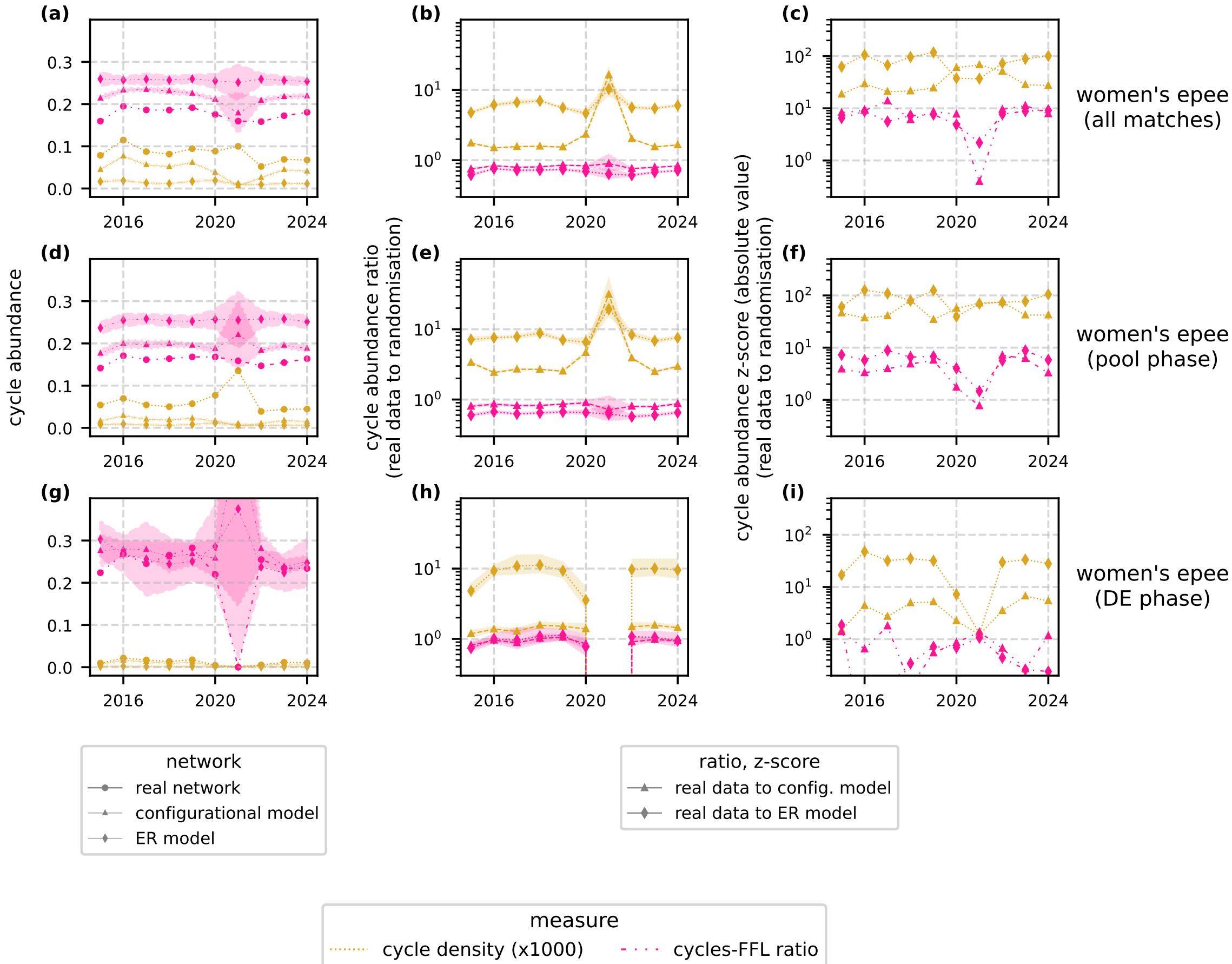


**Figure S14.** **3-cycle abundance in the network of women's épée matches. (a, d, g)** The number of 3-cycles in the graph normed by the number all node triads (yellow), and its ratio to the number of feedforward 3-loops (FFLs) (pink) over time, displaying both the measured value in the original networks (circles), and also the average value in randomised networks according to the configuration model (triangles) and the Erdős–Rényi model (diamonds). The shaded regions around the averages indicate the standard deviation. **(b, e, h)** The two abundance values measured in the real network shown in panel (a, d, g), divided by the values in the randomised networks according to the configuration model (triangles) and the Erdős–Rényi model (diamonds). **(c, f, i)** The two abundance values measured in the real network shown in panel (a, d, g), transformed as a z-score over the distribution of the values in the randomised networks according to the configuration model (triangles) and the Erdős–Rényi model (diamonds).

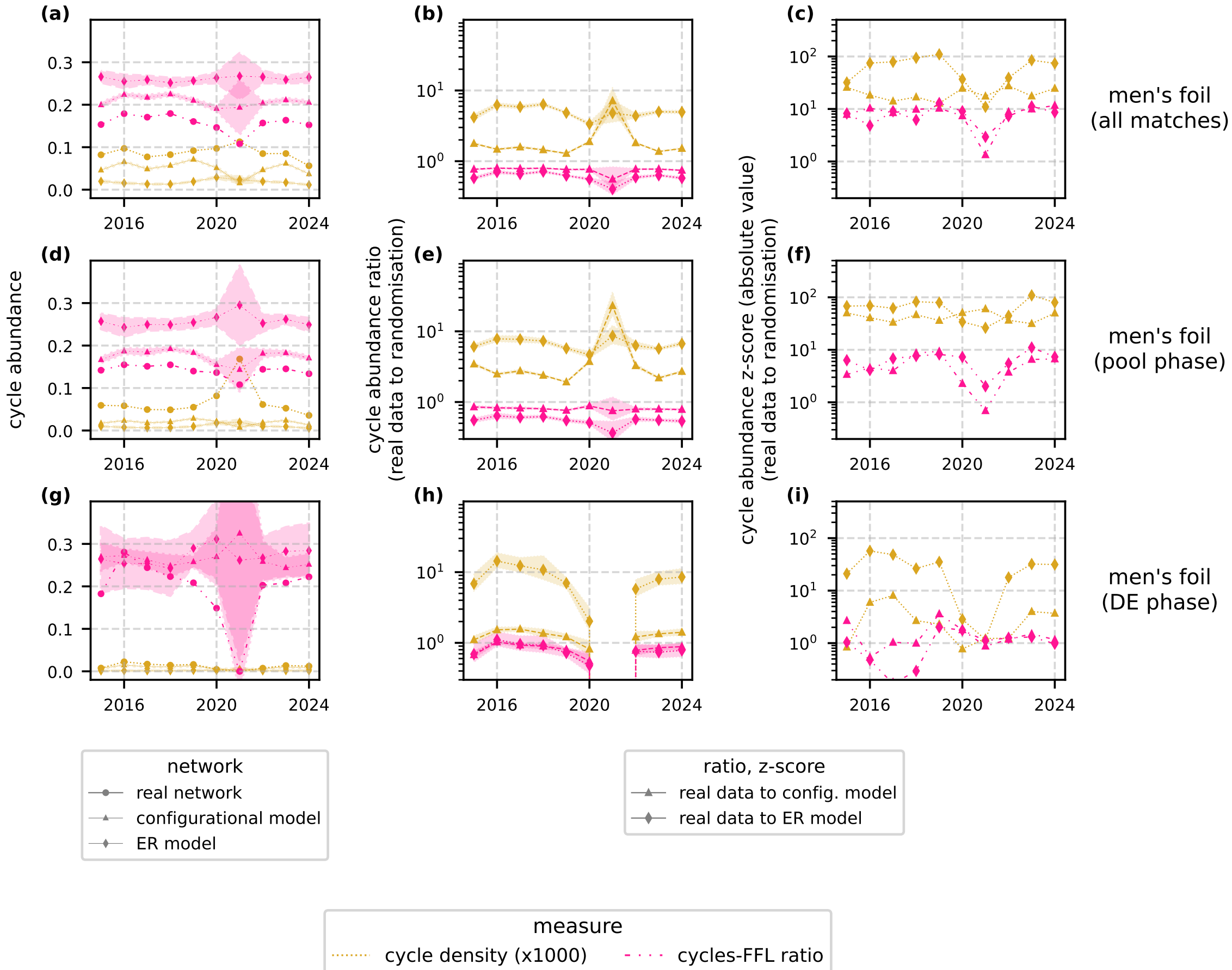


**Figure S15. 3-cycle abundance in the network of men's foil matches. (a, d, g)** The number of 3-cycles in the graph normed by the number all node triads (yellow), and its ratio to the number of feedforward 3-loops (FFLs) (pink) over time, displaying both the measured value in the original networks (circles), and also the average value in randomised networks according to the configuration model (triangles) and the Erdős–Rényi model (diamonds). The shaded regions around the averages indicate the standard deviation. **(b, e, h)** The two abundance values measured in the real network shown in panel (a, d, g), divided by the values in the randomised networks according to the configuration model (triangles) and the Erdős–Rényi model (diamonds). **(c, f, i)** The two abundance values measured in the real network shown in panel (a, d, g), transformed as a z-score over the distribution of the values in the randomised networks according to the configuration model (triangles) and the Erdős–Rényi model (diamonds).

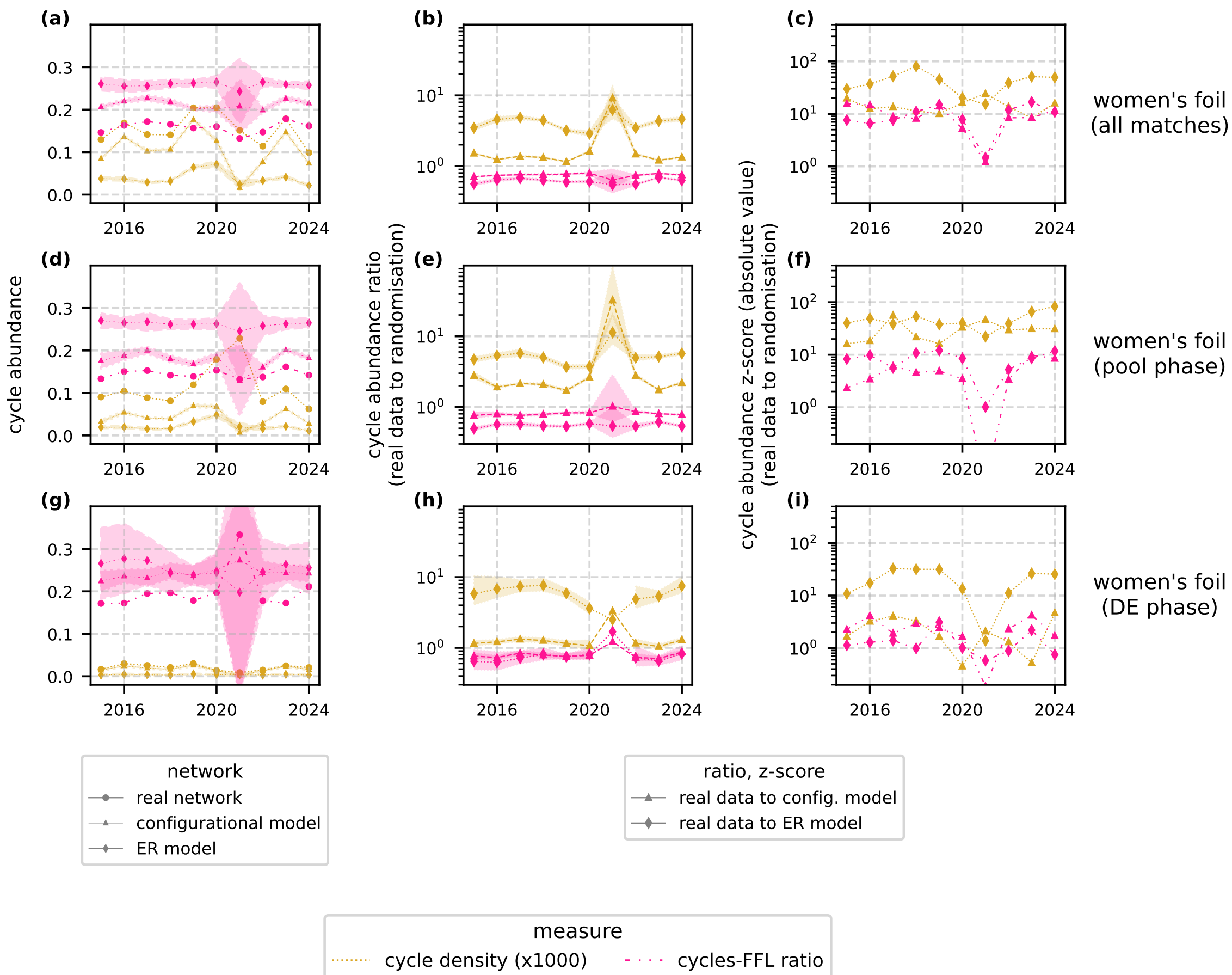


**Figure S16. 3-cycle abundance in the network of women's foil matches. (a, d, g)** The number of 3-cycles in the graph normed by the number all node triads (yellow), and its ratio to the number of feedforward 3-loops (FFLs) (pink) over time, displaying both the measured value in the original networks (circles), and also the average value in randomised networks according to the configuration model (triangles) and the Erdős–Rényi model (diamonds). The shaded regions around the averages indicate the standard deviation. **(b, e, h)** The two abundance values measured in the real network shown in panel (a, d, g), divided by the values in the randomised networks according to the configuration model (triangles) and the Erdős–Rényi model (diamonds). **(c, f, i)** The two abundance values measured in the real network shown in panel (a, d, g), transformed as a z-score over the distribution of the values in the randomised networks according to the configuration model (triangles) and the Erdős–Rényi model (diamonds).

## Cycle enrichment in the network of matches

In Figure 3 of the main text, we plotted the cycle enrichment calculated with SFS and ELO rankings over time for the network of matches. This quantity measured how much more 3-cycles are there in the network than expected based on the given ranking distribution. There, we presented the results using only the above two ranking scores, and only for men's tennis tournaments, and men's sabre tournaments. In this section, we show the analogous results of all the studied dataset in Figures S17-S23.

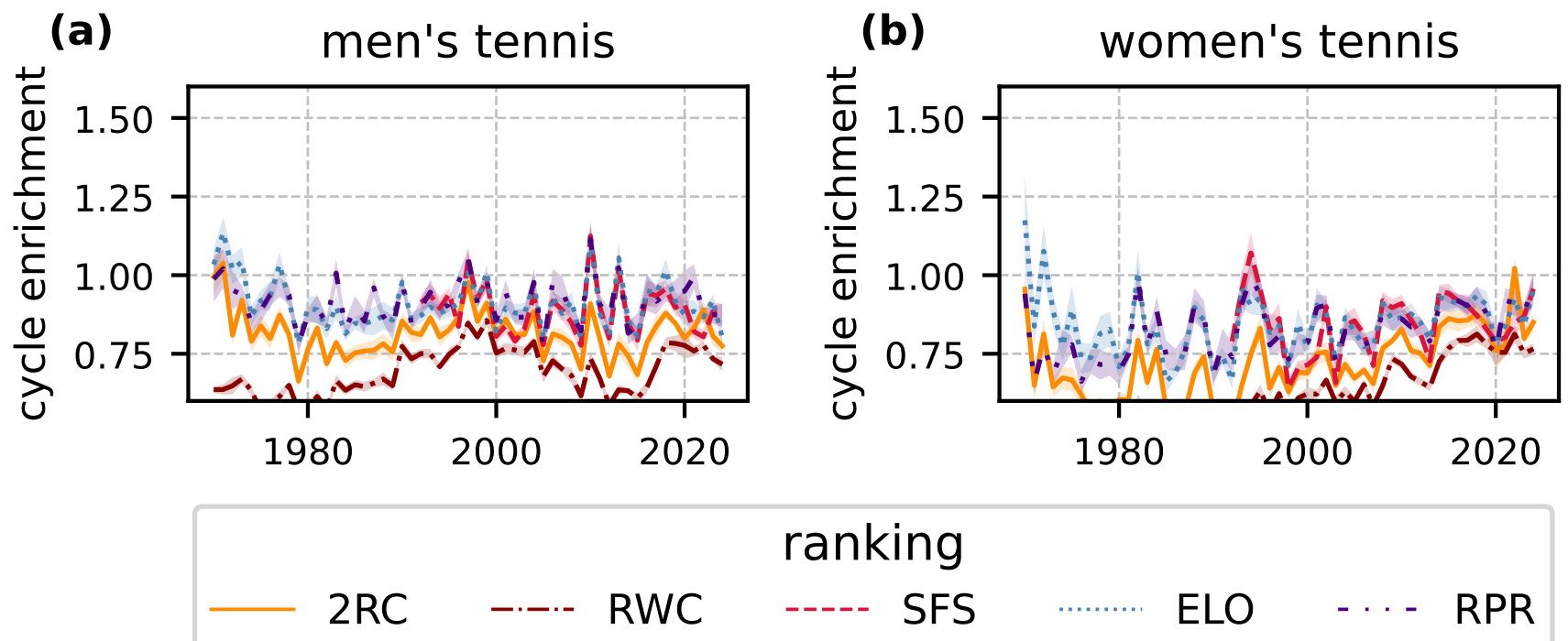


**Figure S17. Cycle enrichment calculated with all ranking scores in the network of matches.** The fraction of the number of 3-cycles and the expected number of 3-cycles from a given player strength distribution. The shaded regions around the averages indicate the standard deviation. **(a)** The cycle enrichment values for the network of men's tennis matches over the years. **(b)** The cycle enrichment values for the network of men's tennis matches over the years.

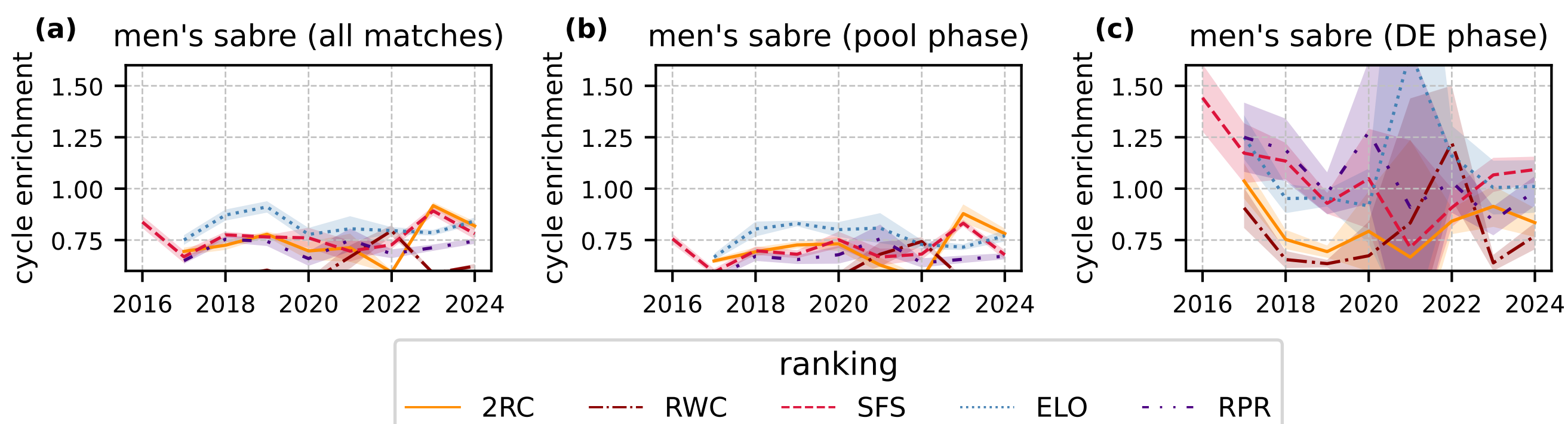


**Figure S18. Cycle enrichment calculated with all ranking scores in the network of matches.** The fraction of the number of 3-cycles and the expected number of 3-cycles from a given player strength distribution. The shaded regions around the averages indicate the standard deviation. **(a)** The cycle enrichment values for the network of men's sabre matches over the years. **(b)** The cycle enrichment values for the network of matches played in the pool phase in men's sabre tournaments. **(c)** The cycle enrichment values for the network of matches played in the direct elimination phase in men's sabre tournaments.

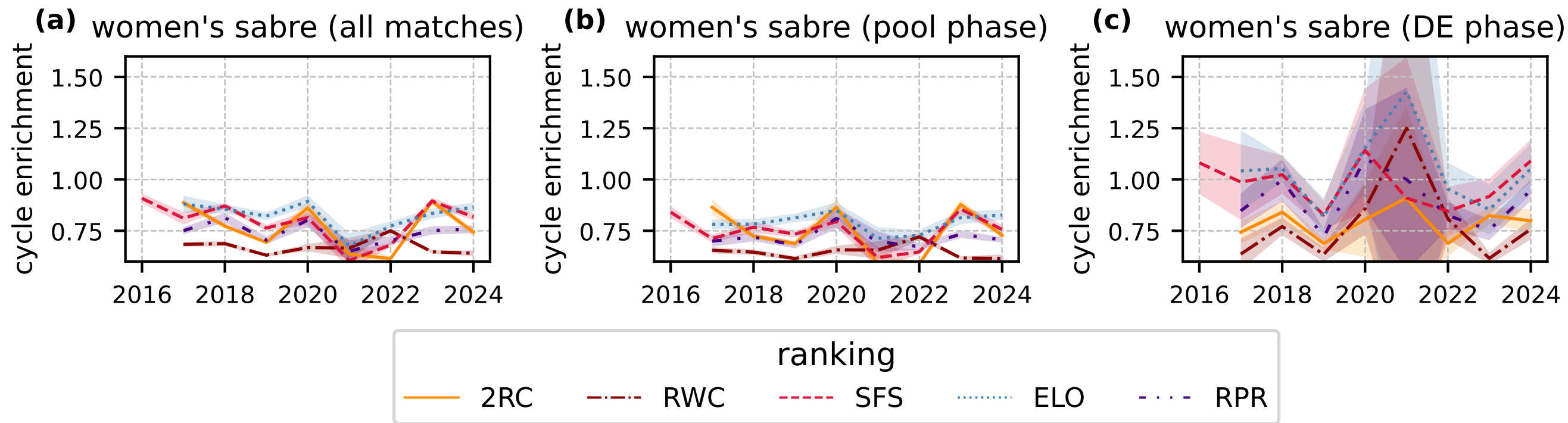


**Figure S19.** **Cycle enrichment calculated with all ranking scores in the network of matches.** The fraction of the number of 3-cycles and the expected number of 3-cycles from a given player strength distribution. The shaded regions around the averages indicate the standard deviation. **(a)** The cycle enrichment values for the network of women's sabre matches over the years. **(b)** The cycle enrichment values for the network of matches played in the pool phase in women's sabre tournaments. **(c)** The cycle enrichment values for the network of matches played in the direct elimination phase in women's sabre tournaments.

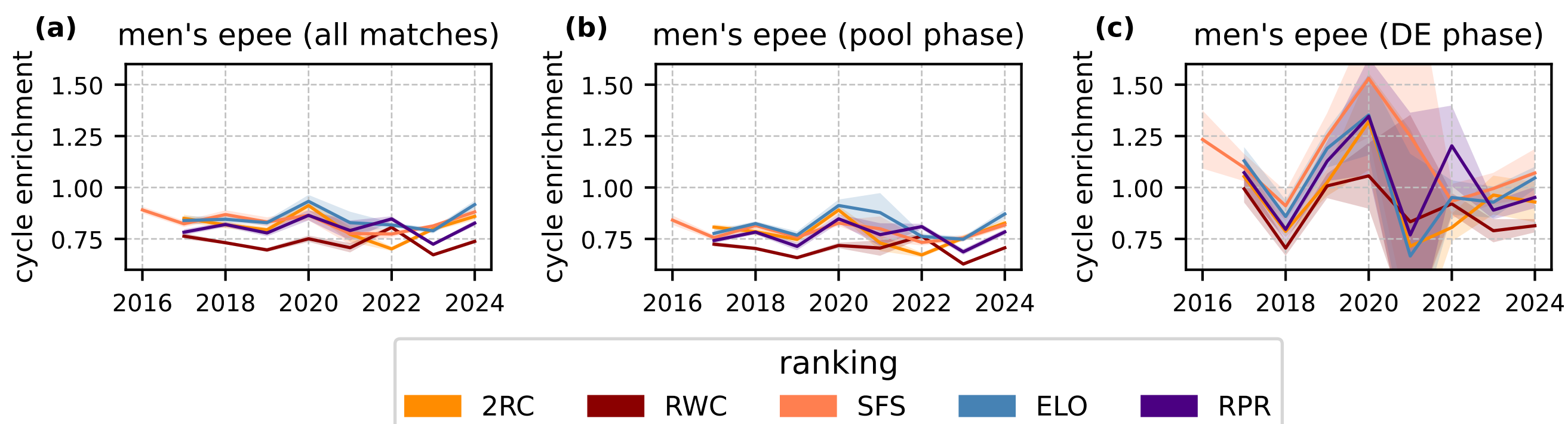


**Figure S20.** **Cycle enrichment calculated with all ranking scores in the network of matches.** The fraction of the number of 3-cycles and the expected number of 3-cycles from a given player strength distribution. The shaded regions around the averages indicate the standard deviation. **(a)** The cycle enrichment values for the network of men's épée matches over the years. **(b)** The cycle enrichment values for the network of matches played in the pool phase in men's épée tournaments. **(c)** The cycle enrichment values for the network of matches played in the direct elimination phase in men's épée tournaments.

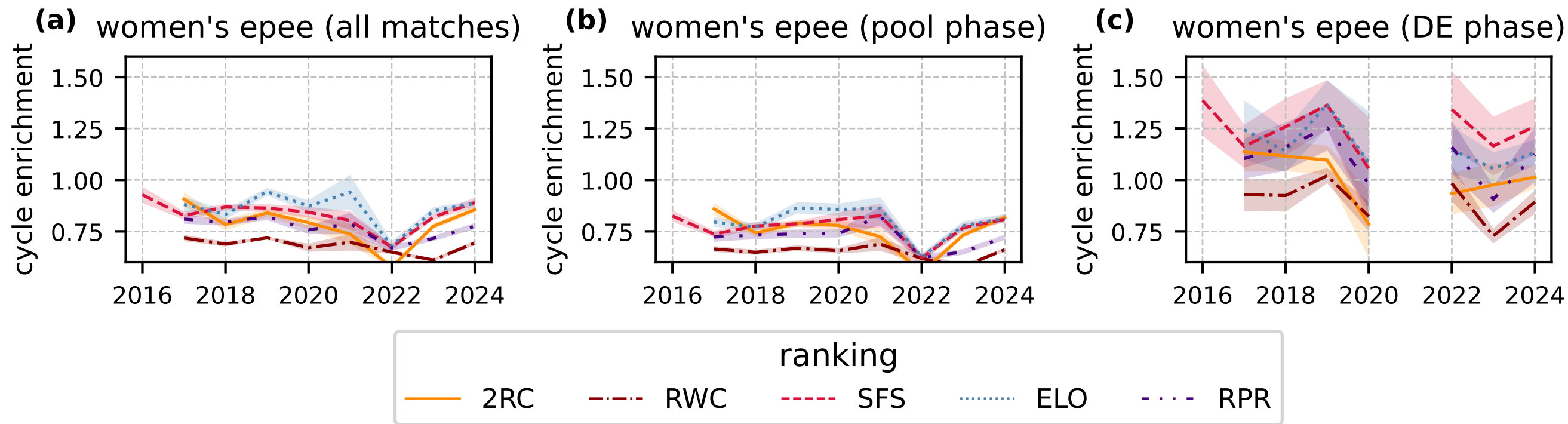


**Figure S21.** **Cycle enrichment calculated with all ranking scores in the network of matches.** The fraction of the number of 3-cycles and the expected number of 3-cycles from a given player strength distribution. The shaded regions around the averages indicate the standard deviation. **(a)** The cycle enrichment values for the network of women's épée matches over the years. **(b)** The cycle enrichment values for the network of matches played in the pool phase in women's épée tournaments. **(c)** The cycle enrichment values for the network of matches played in the direct elimination phase in women's épée tournaments.

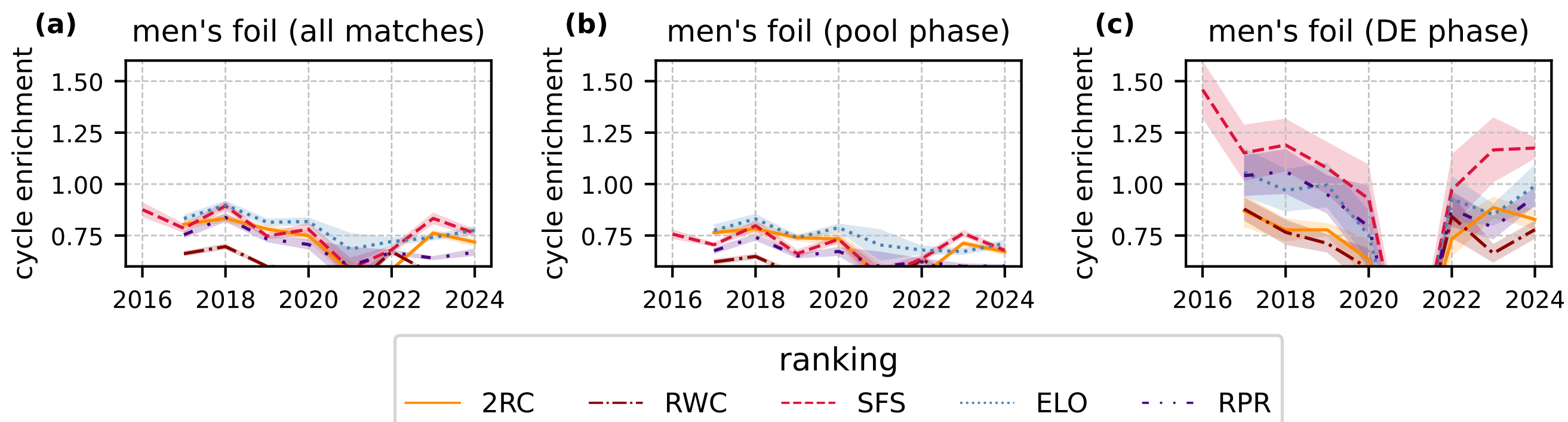


**Figure S22.** **Cycle enrichment calculated with all ranking scores in the network of matches.** The fraction of the number of 3-cycles and the expected number of 3-cycles from a given player strength distribution. The shaded regions around the averages indicate the standard deviation. **(a)** The cycle enrichment values for the network of men's foil matches over the years. **(b)** The cycle enrichment values for the network of matches played in the pool phase in men's foil tournaments. **(c)** The cycle enrichment values for the network of matches played in the direct elimination phase in men's foil tournaments.

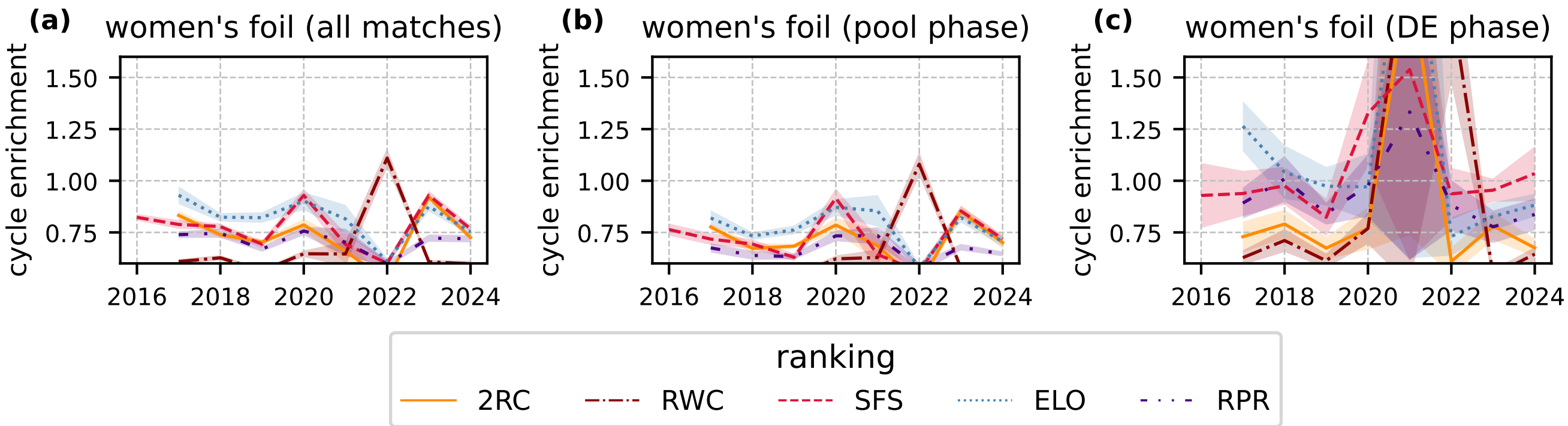


**Figure S23. Cycle enrichment calculated with all ranking scores in the network of matches.** The fraction of the number of 3-cycles and the expected number of 3-cycles from a given player strength distribution. The shaded regions around the averages indicate the standard deviation. **(a)** The cycle enrichment values for the network of women's foil matches over the years. **(b)** The cycle enrichment values for the network of matches played in the pool phase in women's foil tournaments. **(c)** The cycle enrichment values for the network of matches played in the direct elimination phase in women's foil tournaments.

## The structure of the hierarchy

In Figures 4-5 of the main text, we presented the structure of the hierarchy in the network of men's tennis and men's sabre matches in 2015, using 5 different ranking scores. Such illustration can be done for the network of matches of any studied sport and from any year. In this section, we show the analogous results for all studied sports from the years 2015 and 2023 in Figures S24-S39.

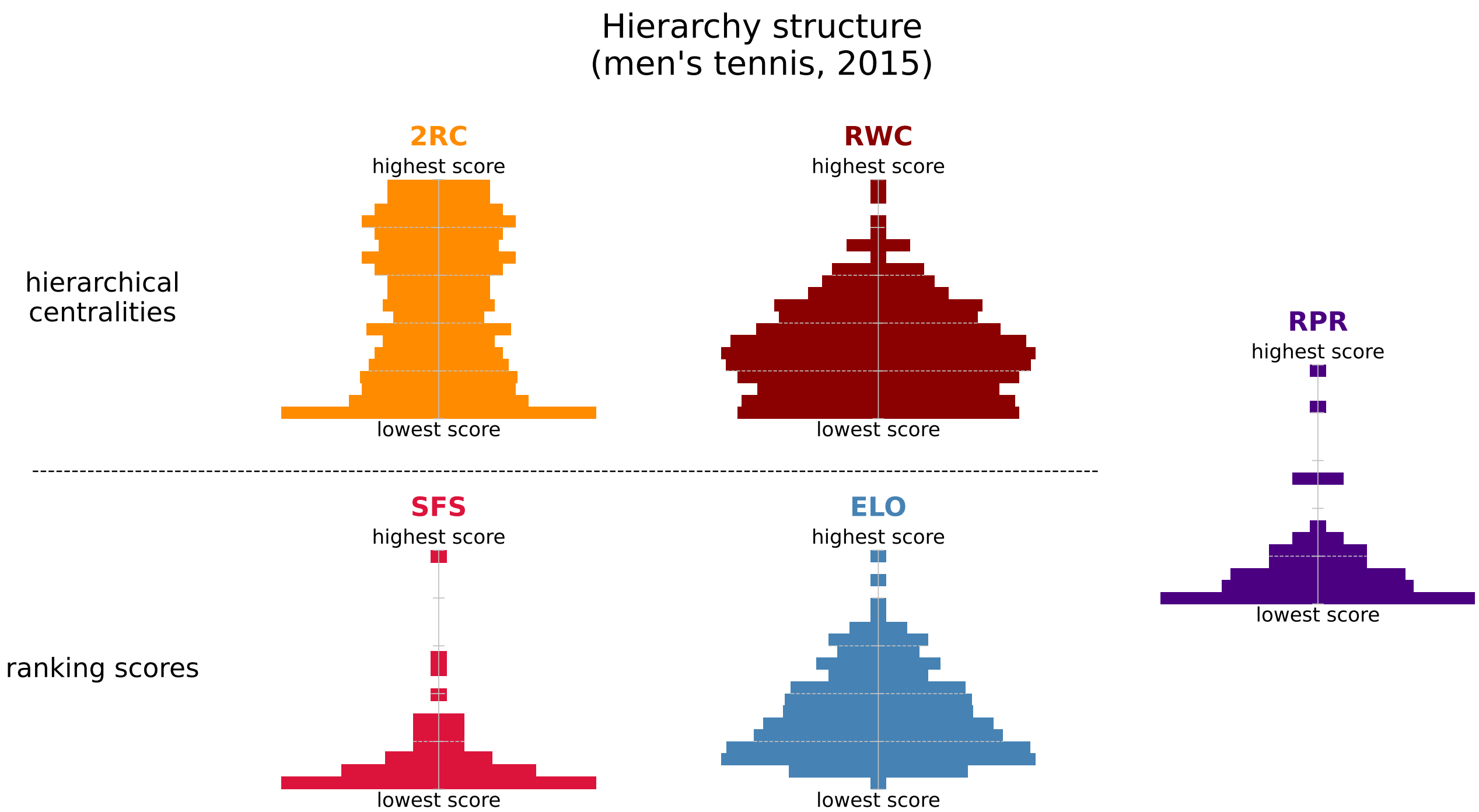


**Figure S24. The structure of the hierarchy for men's tennis in 2015.** The distribution of nodes along the 2-reach centrality (orange), the random walk centrality (red), the official sport ranking (pink), the Elo score (cyan), and the revered PageRank (purple) for the network of men's tennis matches played in 2015. The width of the bars represents the relative number of nodes having a specific centrality value on log-scale.

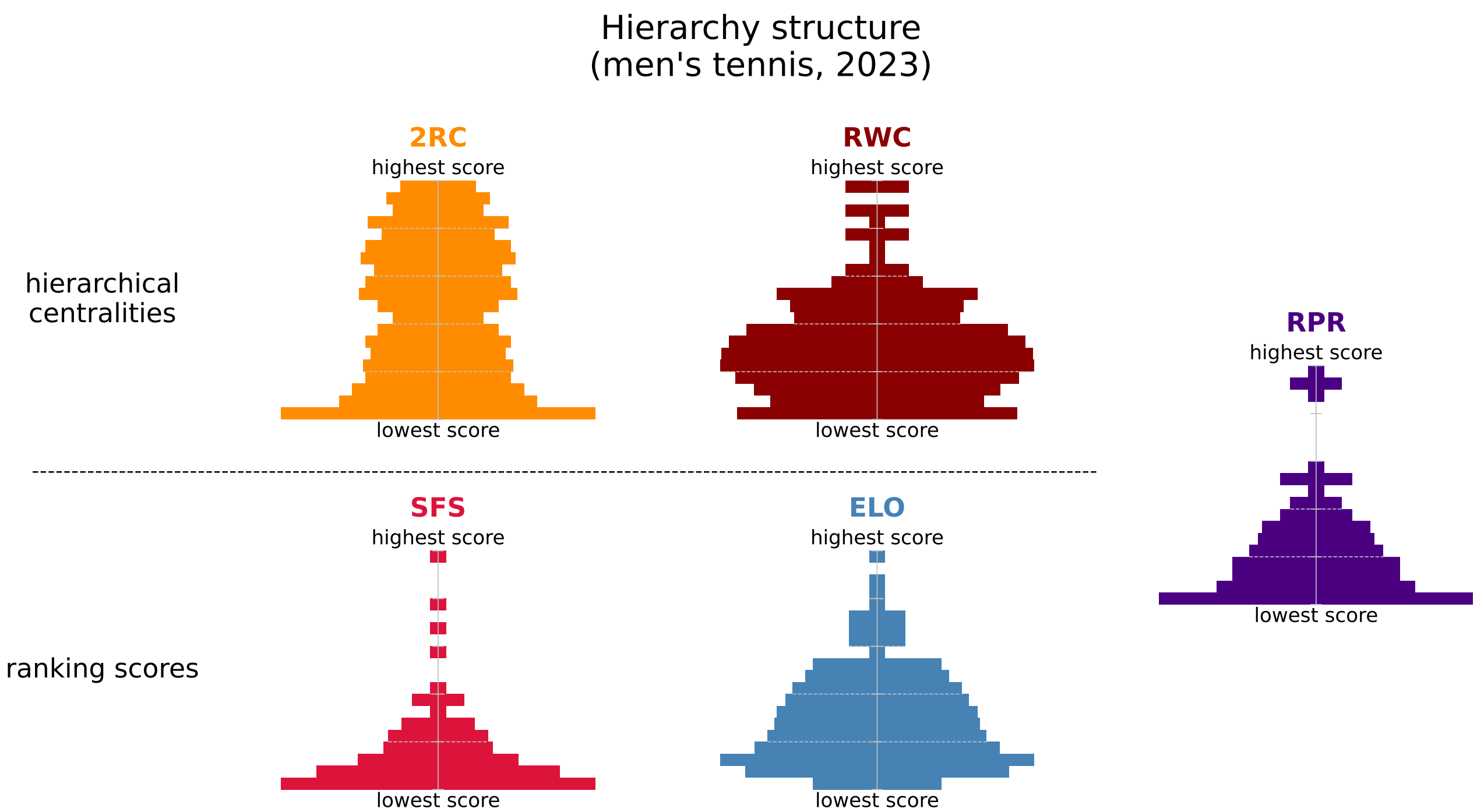


**Figure S25. The structure of the hierarchy for men's tennis in 2023.** The distribution of nodes along the 2-reach centrality (orange), the random walk centrality (red), the official sport ranking (pink), the Elo score (cyan), and the revered PageRank (purple) for the network of men's tennis matches played in 2023. The width of the bars represents the relative number of nodes having a specific centrality value on log-scale.

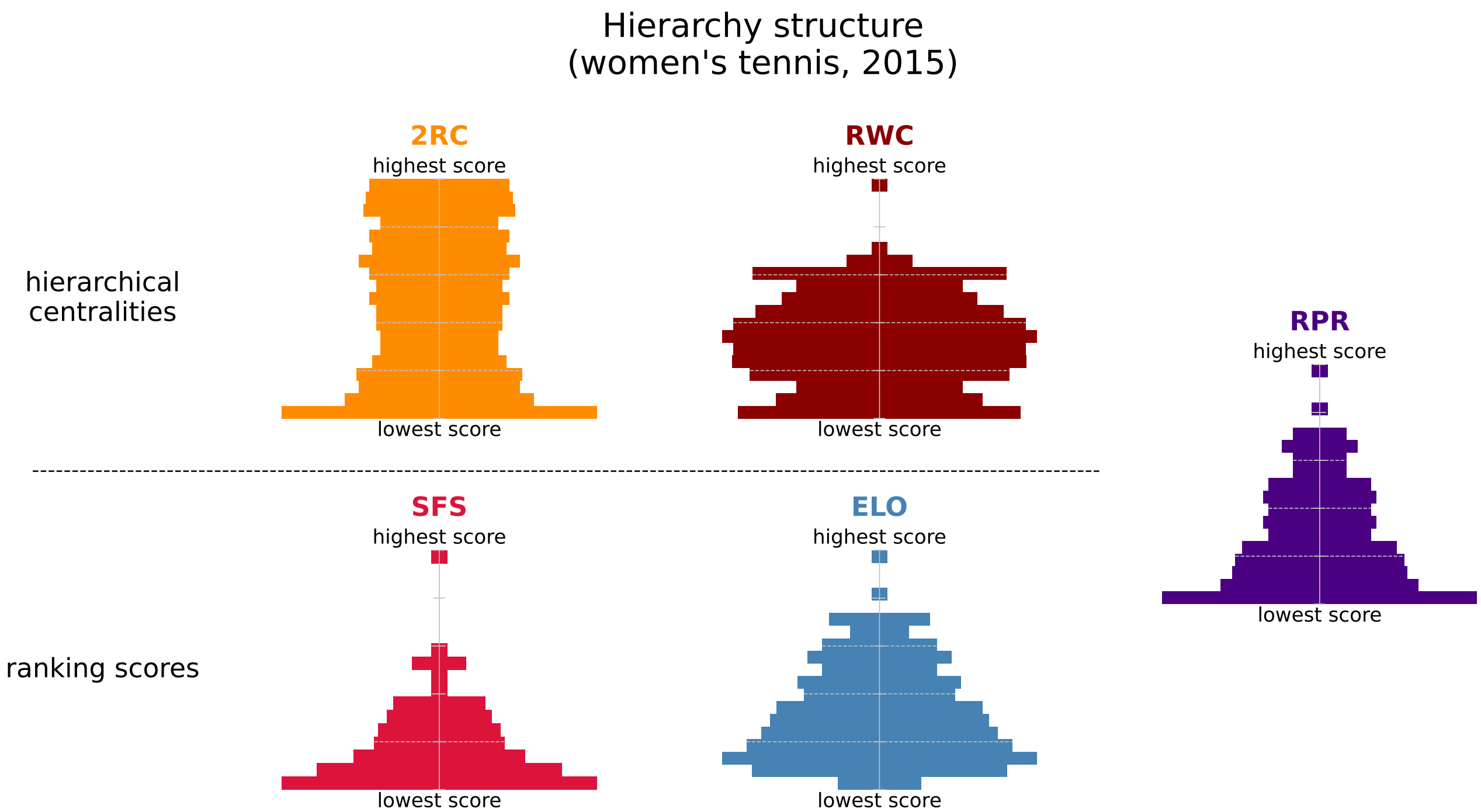


**Figure S26. The structure of the hierarchy for women's tennis in 2015.** The distribution of nodes along the 2-reach centrality (orange), the random walk centrality (red), the official sport ranking (pink), the Elo score (cyan), and the revered PageRank (purple) for the network of women's tennis matches played in 2015. The width of the bars represents the relative number of nodes having a specific centrality value on log-scale.

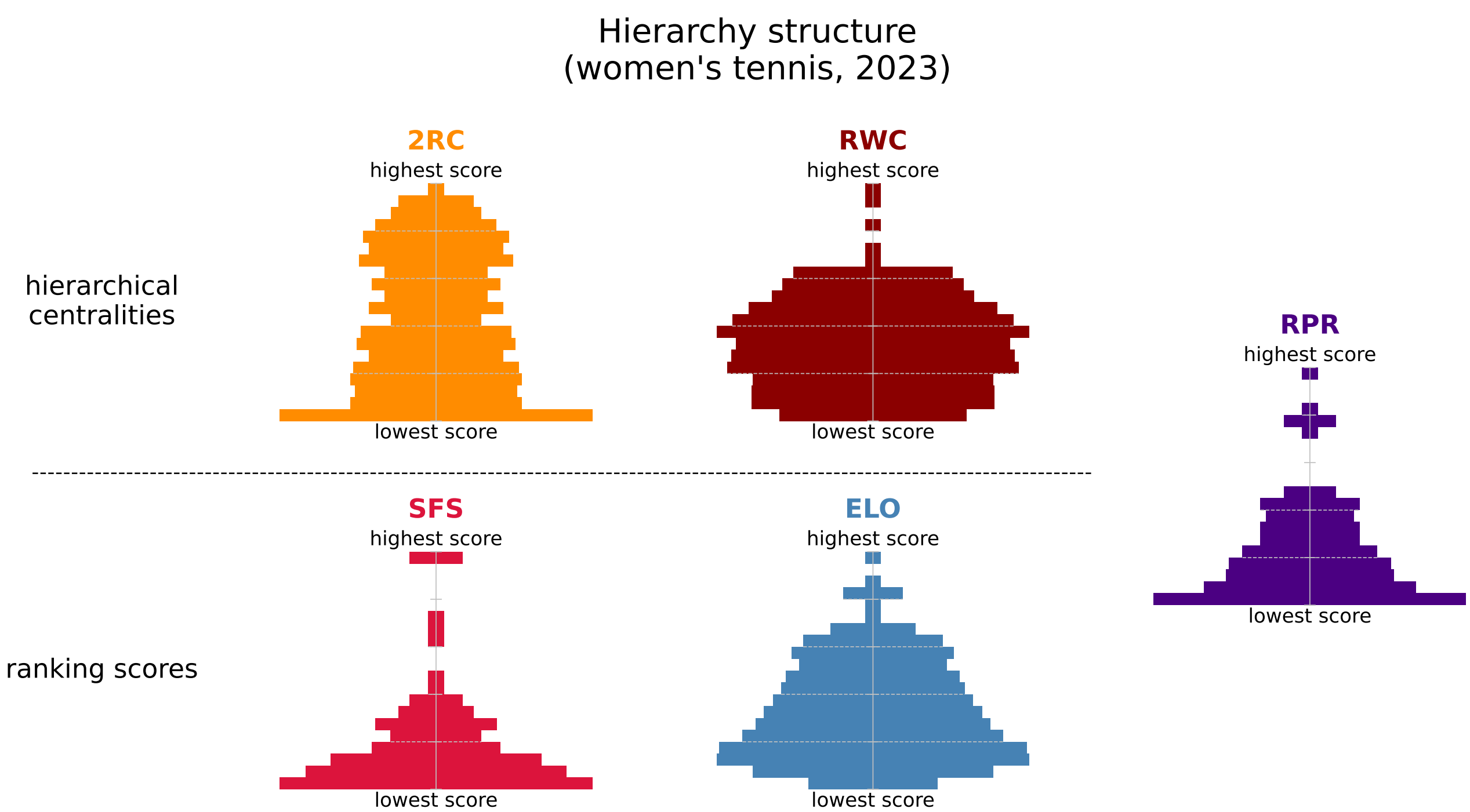


**Figure S27. The structure of the hierarchy for women's tennis in 2023.** The distribution of nodes along the 2-reach centrality (orange), the random walk centrality (red), the official sport ranking (pink), the Elo score (cyan), and the revered PageRank (purple) for the network of women's tennis matches played in 2023. The width of the bars represents the relative number of nodes having a specific centrality value on log-scale.

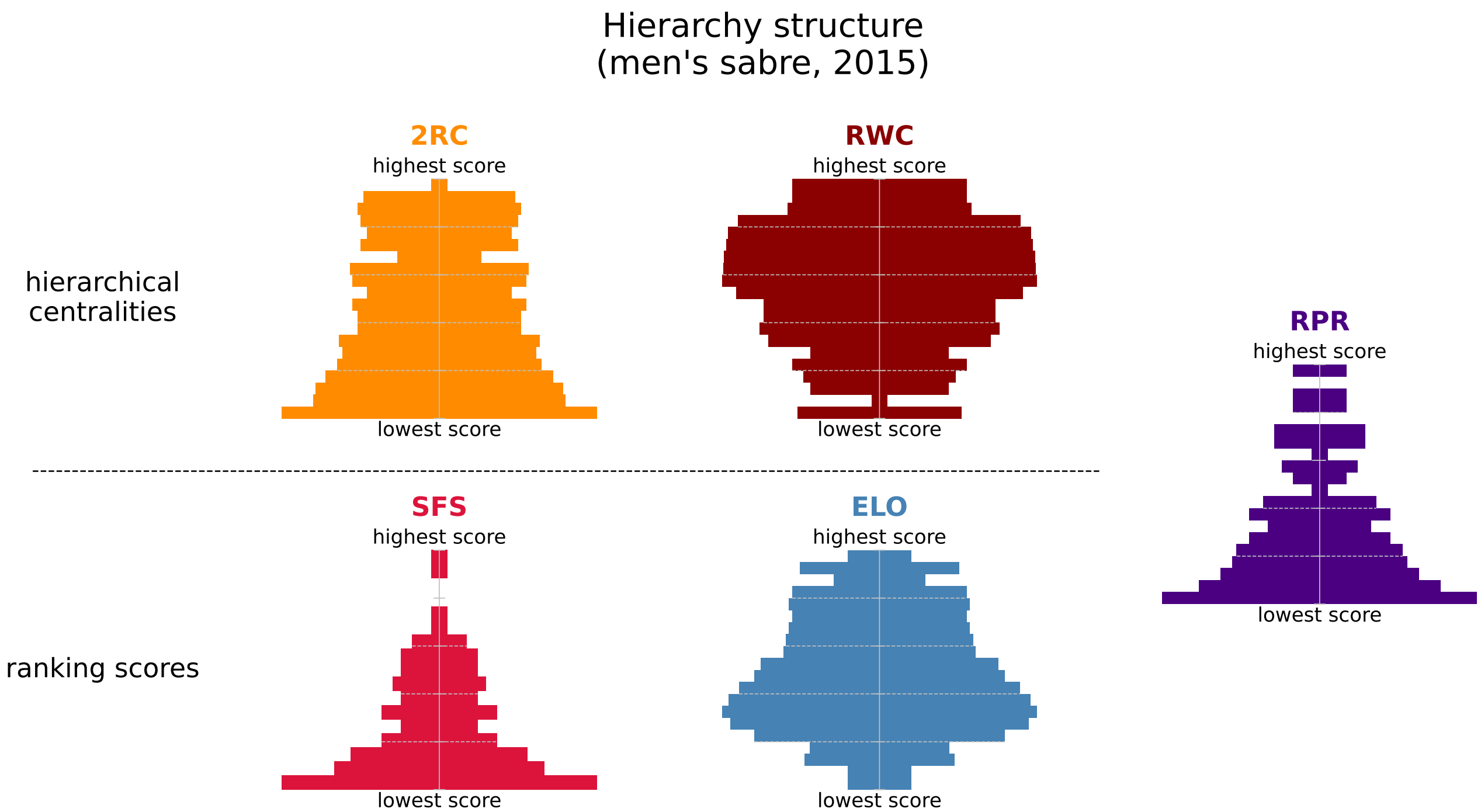


**Figure S28. The structure of the hierarchy for men's sabre in 2015.** The distribution of nodes along the 2-reach centrality (orange), the random walk centrality (red), the official sport ranking (pink), the Elo score (cyan), and the revered PageRank (purple) for the network of men's sabre matches played in 2015. The width of the bars represents the relative number of nodes having a specific centrality value on log-scale.

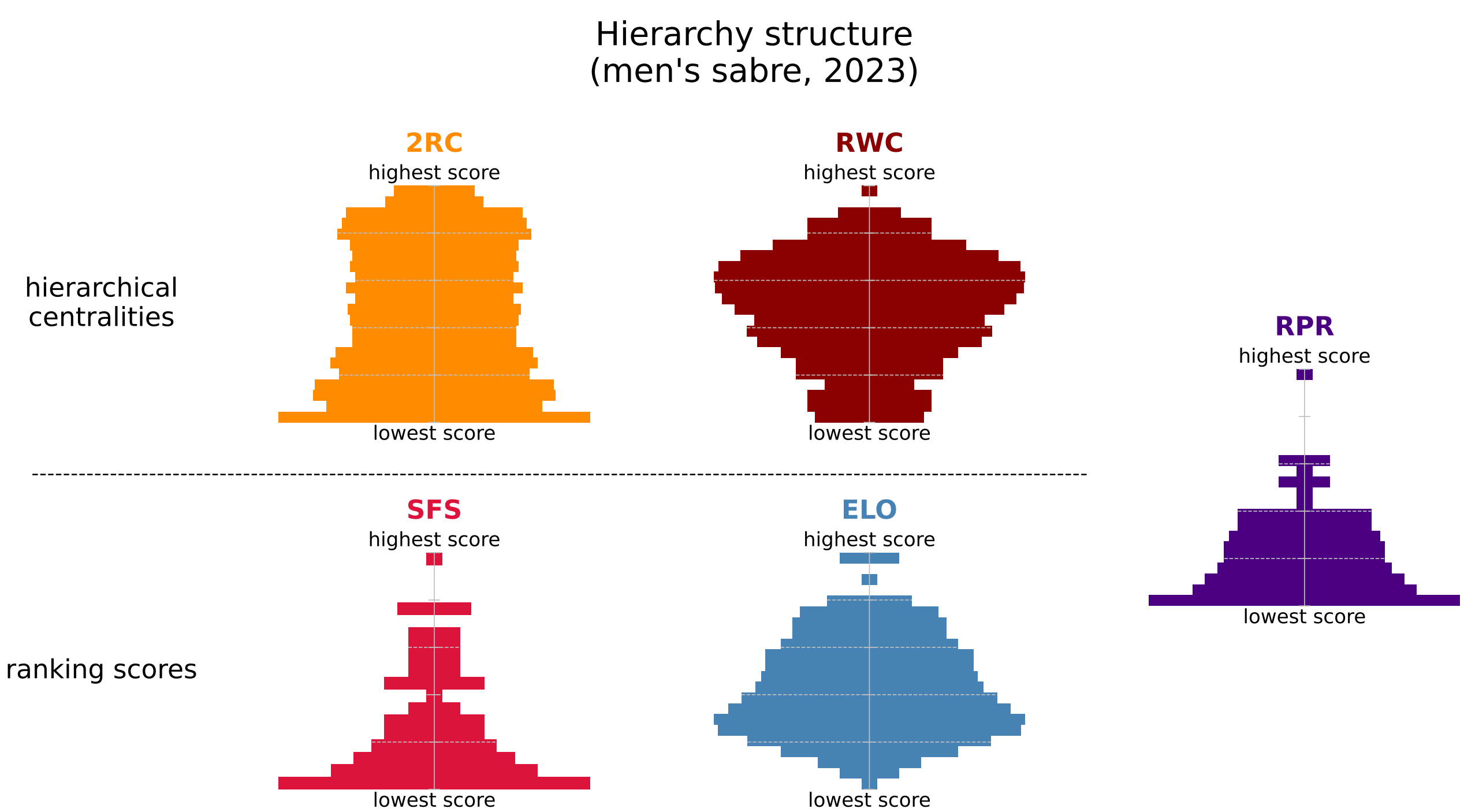


**Figure S29. The structure of the hierarchy for men's sabre in 2023.** The distribution of nodes along the 2-reach centrality (orange), the random walk centrality (red), the official sport ranking (pink), the Elo score (cyan), and the revered PageRank (purple) for the network of men's sabre matches played in 2023. The width of the bars represents the relative number of nodes having a specific centrality value on log-scale.

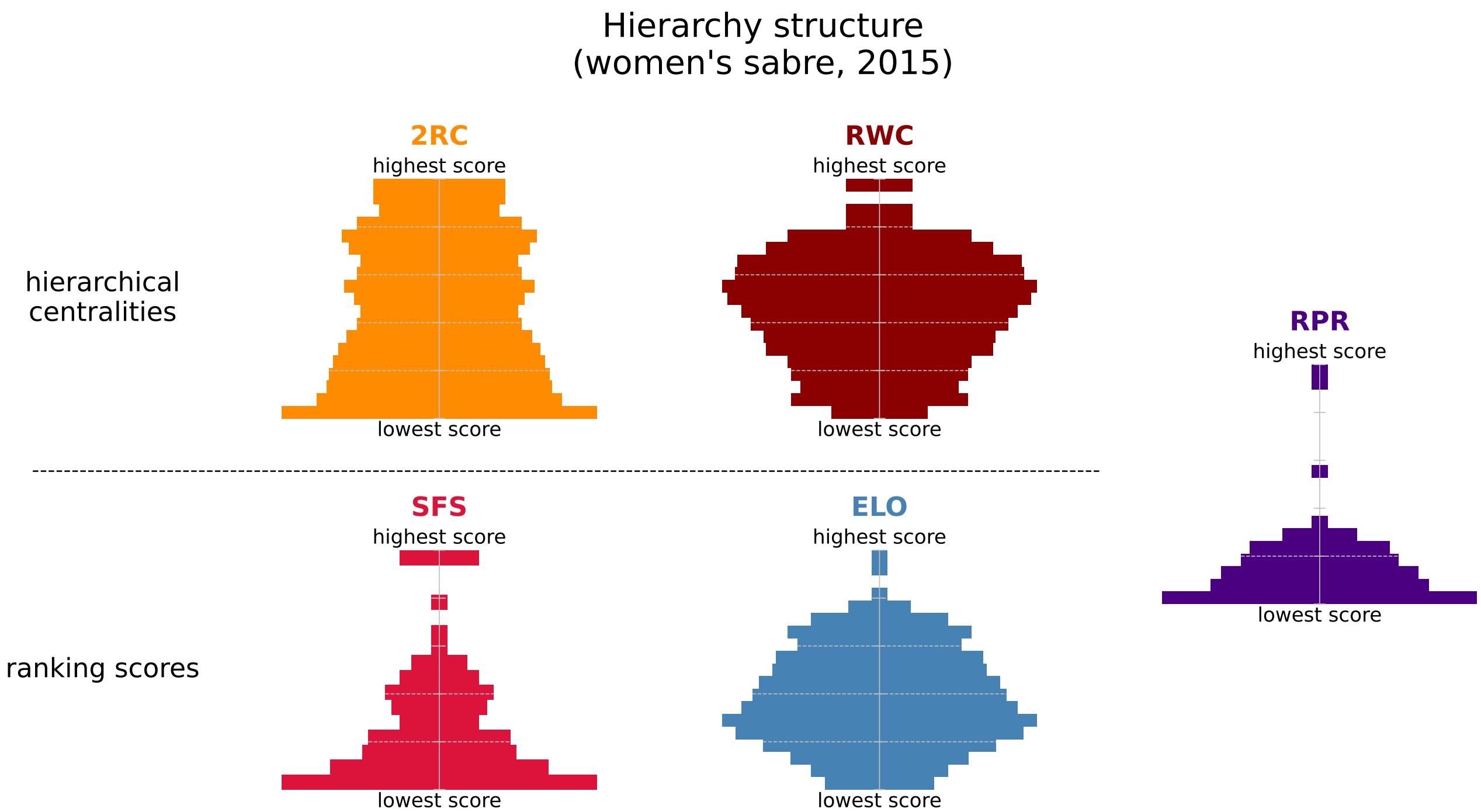


**Figure S30. The structure of the hierarchy for women's sabre in 2015.** The distribution of nodes along the 2-reach centrality (orange), the random walk centrality (red), the official sport ranking (pink), the Elo score (cyan), and the revered PageRank (purple) for the network of women's sabre matches played in 2015. The width of the bars represents the relative number of nodes having a specific centrality value on log-scale.

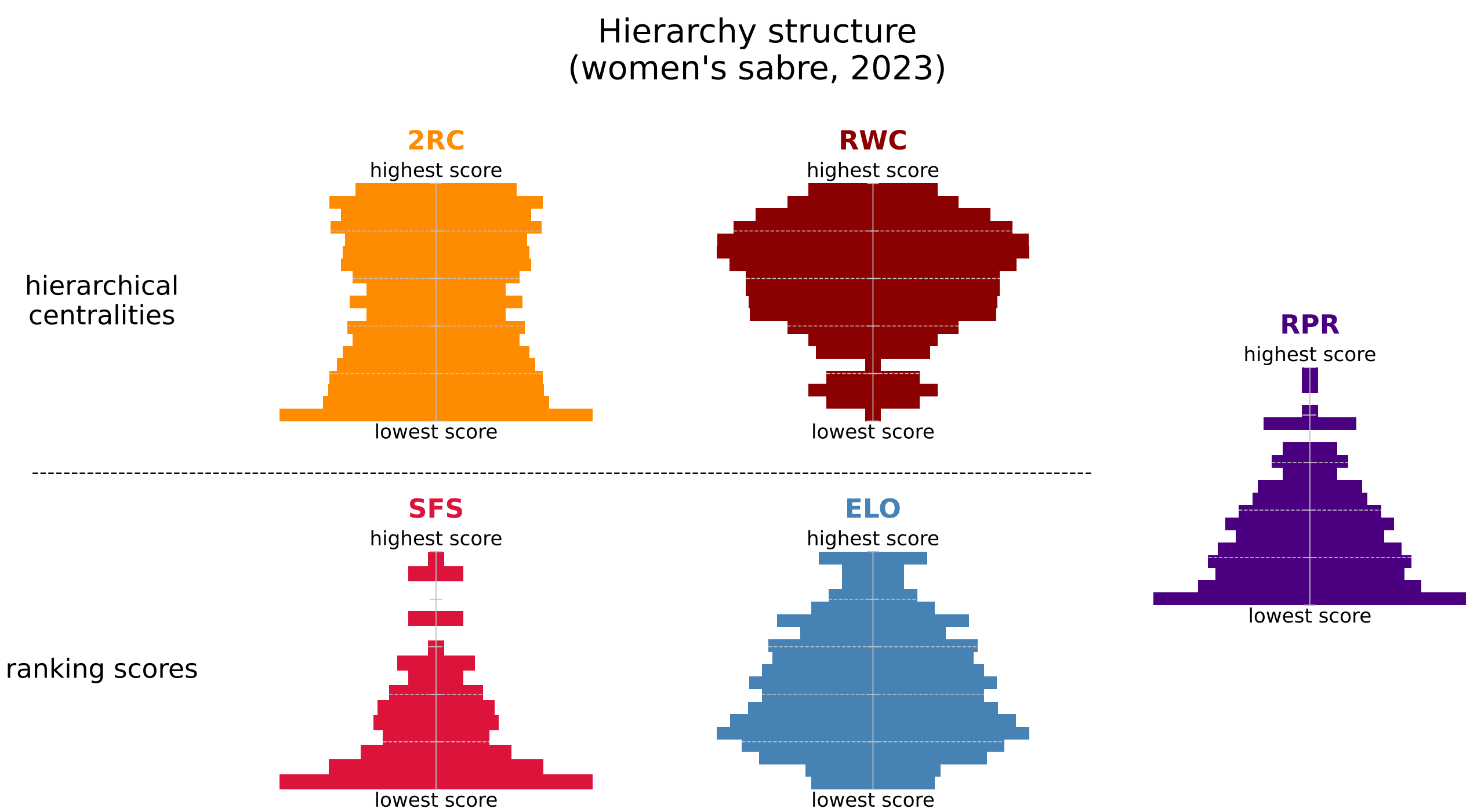


**Figure S31. The structure of the hierarchy for women's sabre in 2023.** The distribution of nodes along the 2-reach centrality (orange), the random walk centrality (red), the official sport ranking (pink), the Elo score (cyan), and the revered PageRank (purple) for the network of women's sabre matches played in 2023. The width of the bars represents the relative number of nodes having a specific centrality value on log-scale.

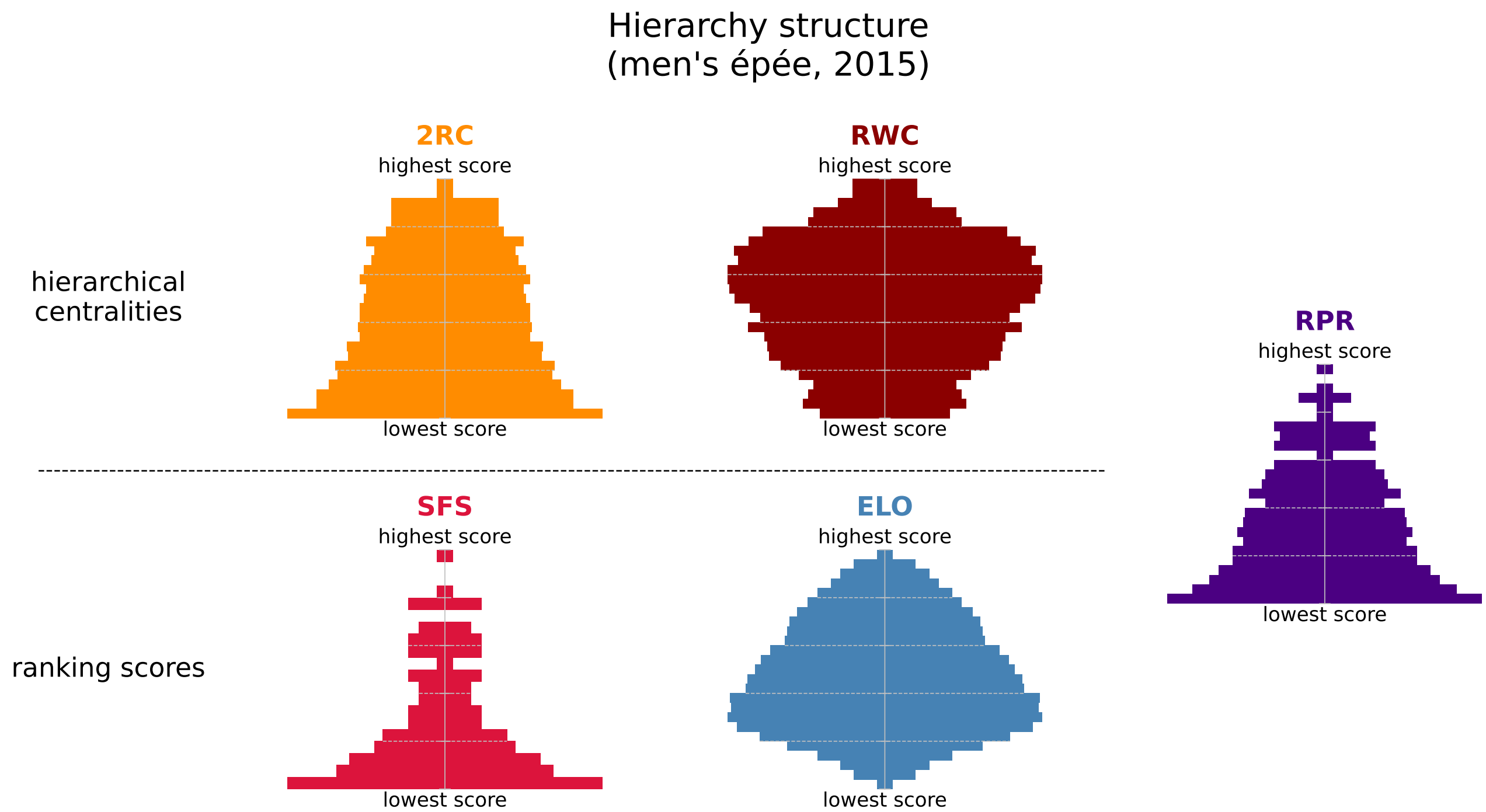


**Figure S32. The structure of the hierarchy for men's épée in 2015.** The distribution of nodes along the 2-reach centrality (orange), the random walk centrality (red), the official sport ranking (pink), the Elo score (cyan), and the revered PageRank (purple) for the network of men's épée matches played in 2015. The width of the bars represents the relative number of nodes having a specific centrality value on log-scale.

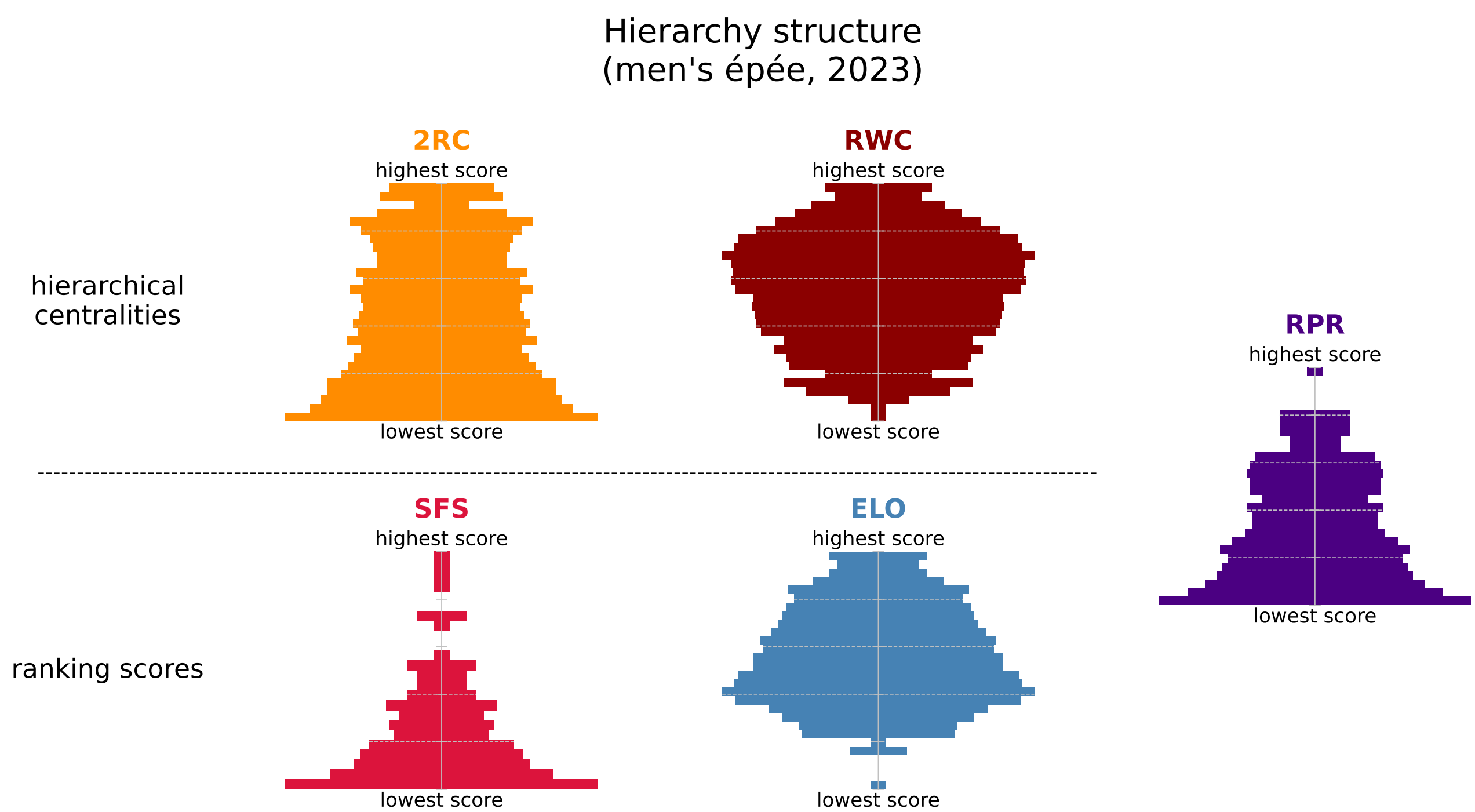


**Figure S33. The structure of the hierarchy for men's épée in 2023.** The distribution of nodes along the 2-reach centrality (orange), the random walk centrality (red), the official sport ranking (pink), the Elo score (cyan), and the revered PageRank (purple) for the network of men's épée matches played in 2023. The width of the bars represents the relative number of nodes having a specific centrality value on log-scale.

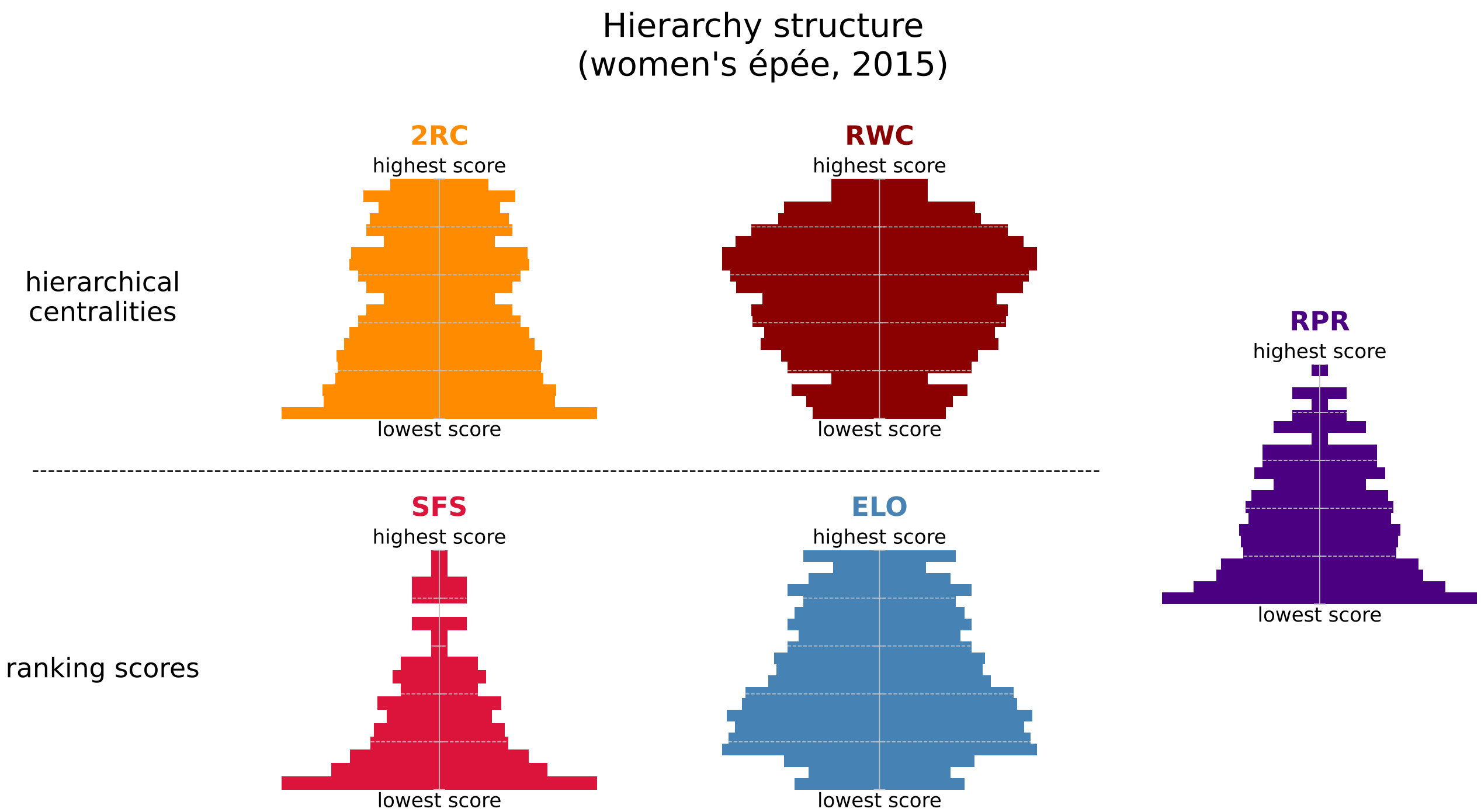


**Figure S34. The structure of the hierarchy for women's épée in 2015.** The distribution of nodes along the 2-reach centrality (orange), the random walk centrality (red), the official sport ranking (pink), the Elo score (cyan), and the revered PageRank (purple) for the network of women's épée matches played in 2015. The width of the bars represents the relative number of nodes having a specific centrality value on log-scale.

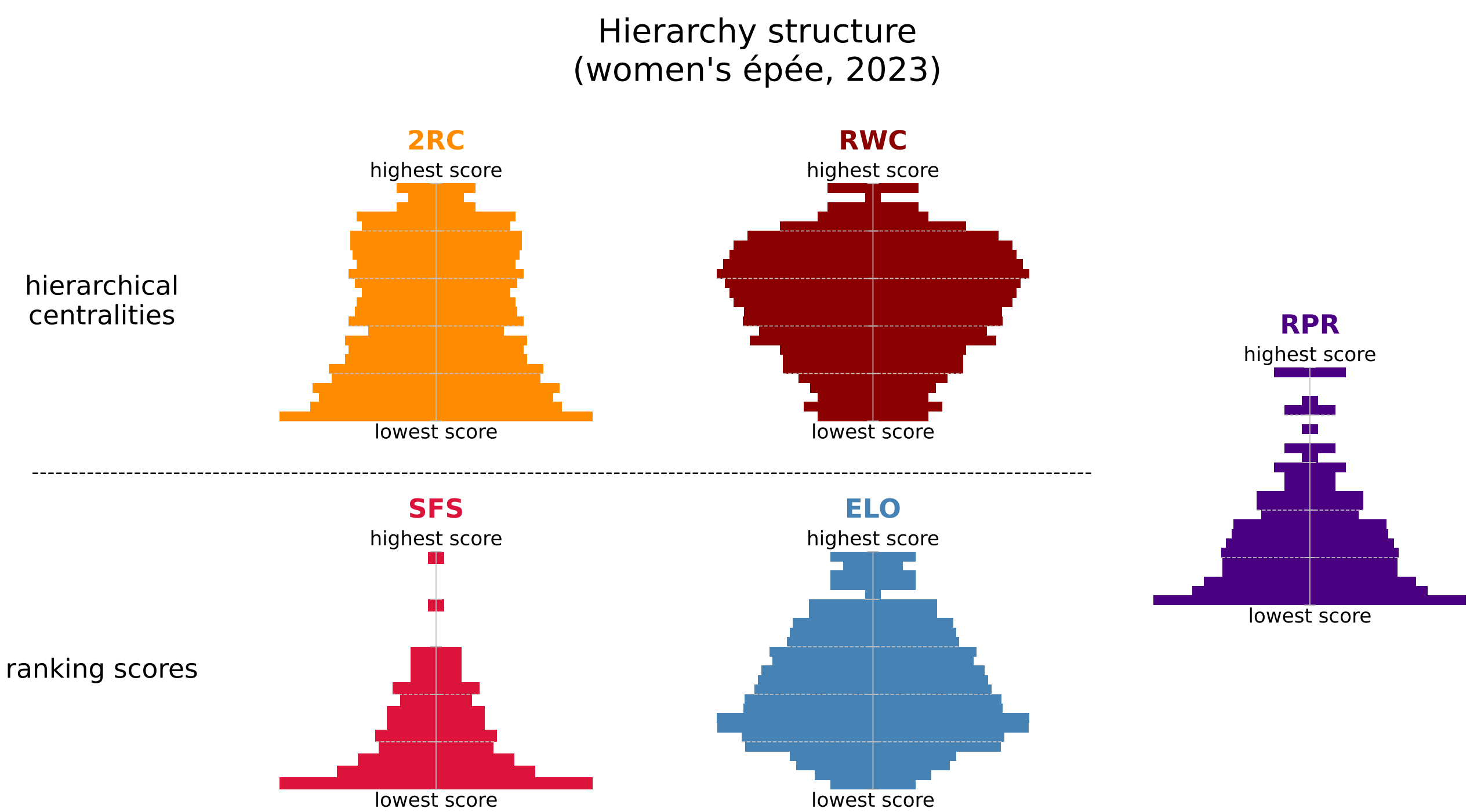


**Figure S35. The structure of the hierarchy for women's épée in 2023.** The distribution of nodes along the 2-reach centrality (orange), the random walk centrality (red), the official sport ranking (pink), the Elo score (cyan), and the revered PageRank (purple) for the network of women's épée matches played in 2023. The width of the bars represents the relative number of nodes having a specific centrality value on log-scale.

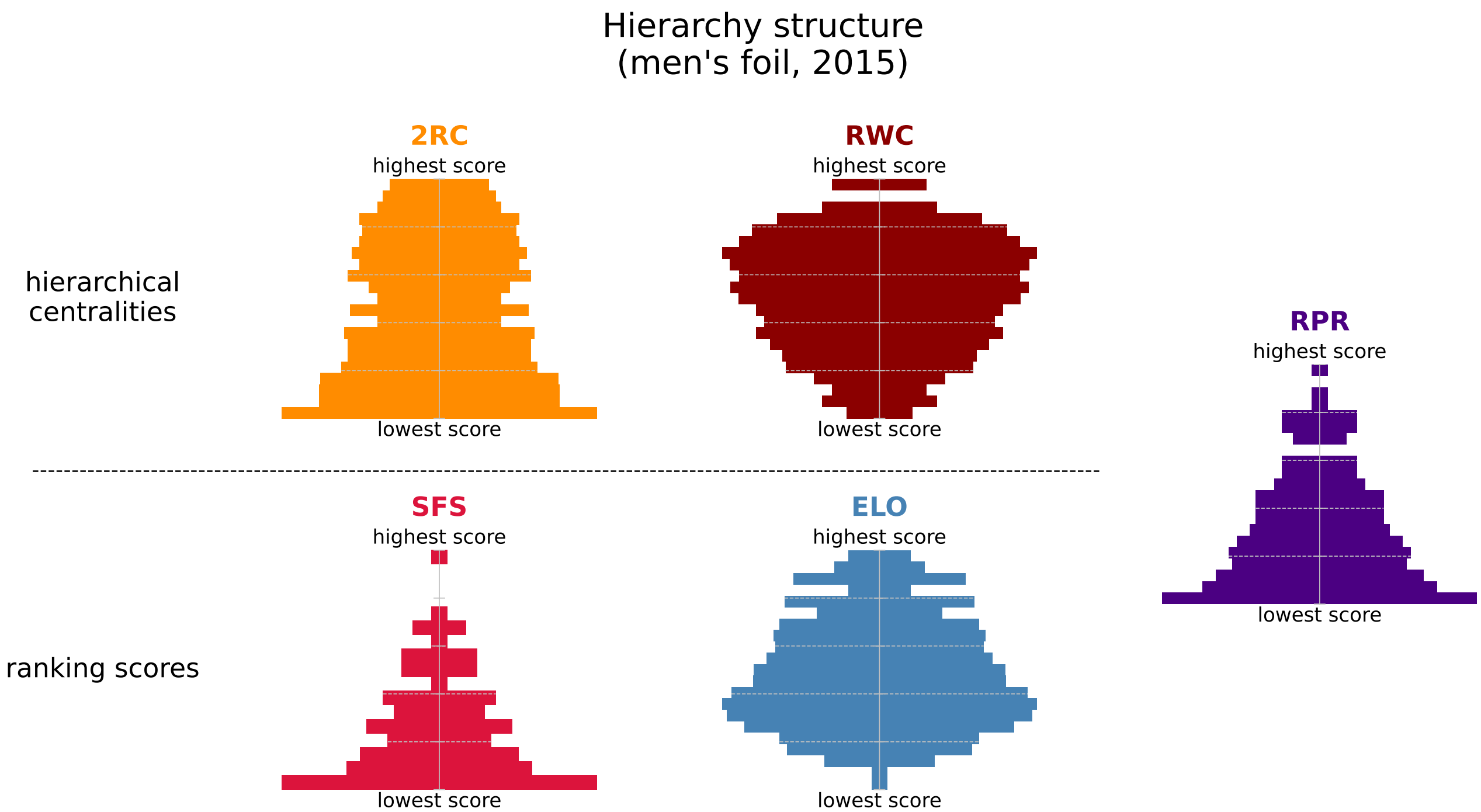


**Figure S36. The structure of the hierarchy for men's foil in 2015.** The distribution of nodes along the 2-reach centrality (orange), the random walk centrality (red), the official sport ranking (pink), the Elo score (cyan), and the revered PageRank (purple) for the network of men's foil matches played in 2015. The width of the bars represents the relative number of nodes having a specific centrality value on log-scale.

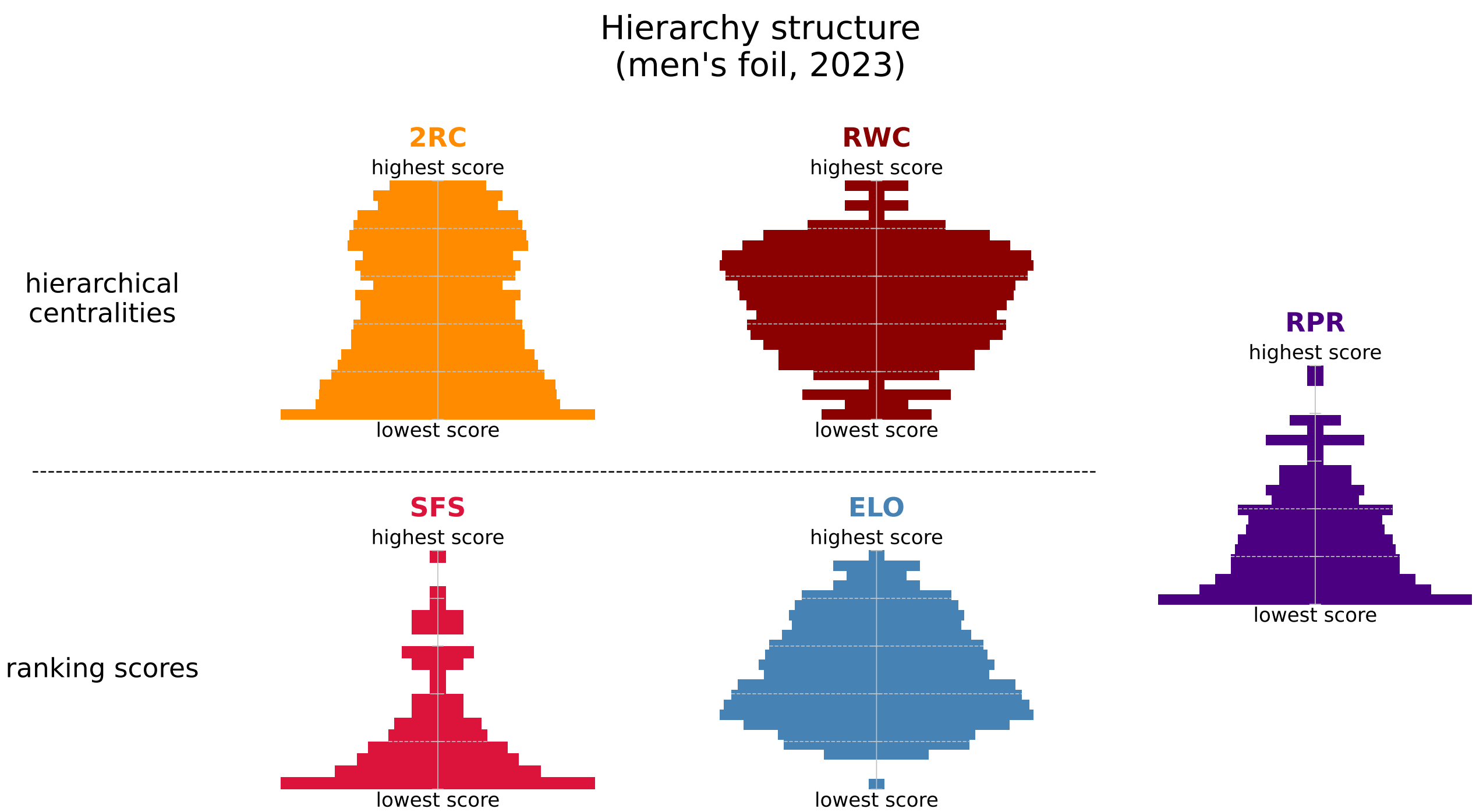


**Figure S37. The structure of the hierarchy for men's foil in 2023.** The distribution of nodes along the 2-reach centrality (orange), the random walk centrality (red), the official sport ranking (pink), the Elo score (cyan), and the revered PageRank (purple) for the network of men's foil matches played in 2023. The width of the bars represents the relative number of nodes having a specific centrality value on log-scale.

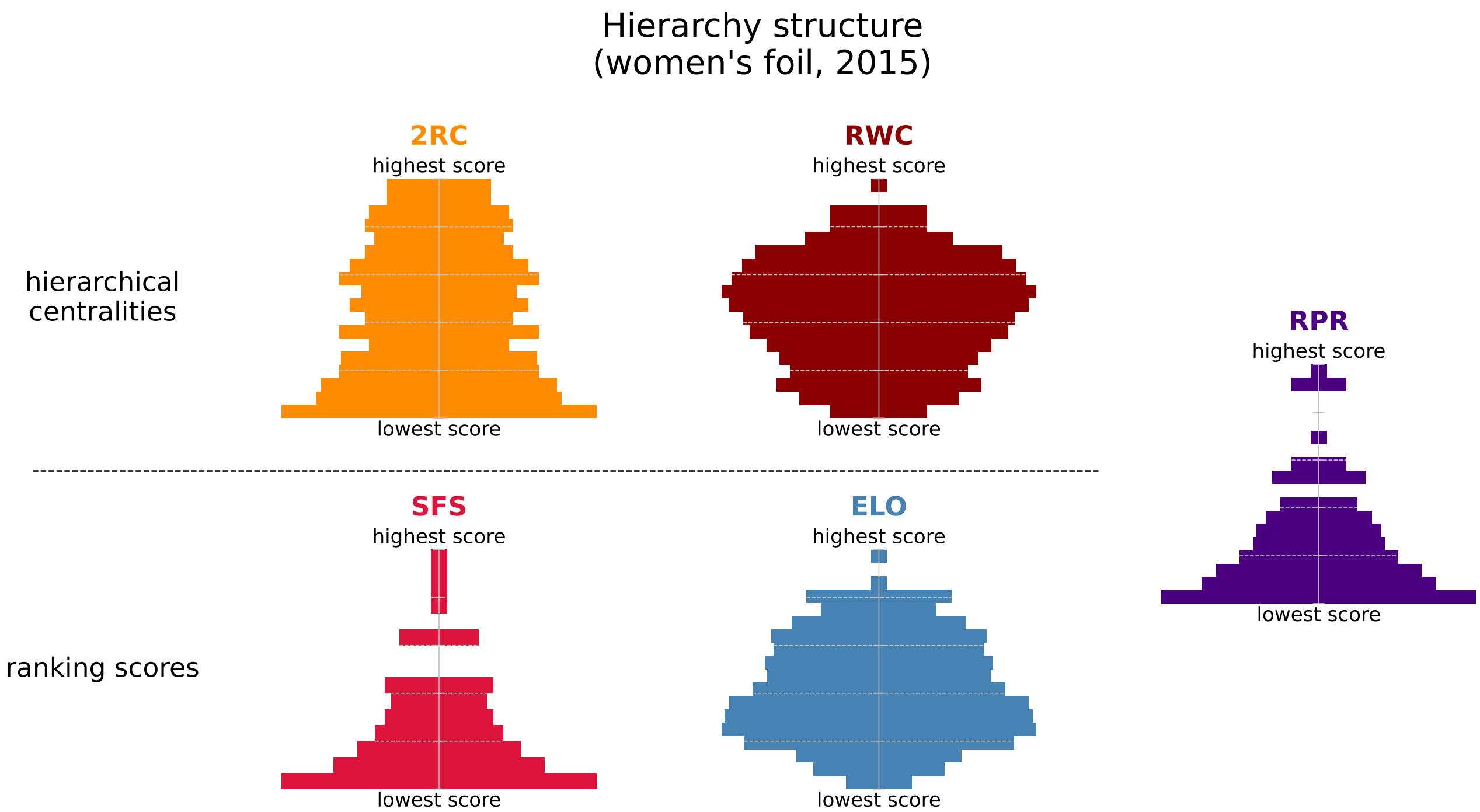


**Figure S38. The structure of the hierarchy for women's foil in 2015.** The distribution of nodes along the 2-reach centrality (orange), the random walk centrality (red), the official sport ranking (pink), the Elo score (cyan), and the revered PageRank (purple) for the network of women's foil matches played in 2015. The width of the bars represents the relative number of nodes having a specific centrality value on log-scale.

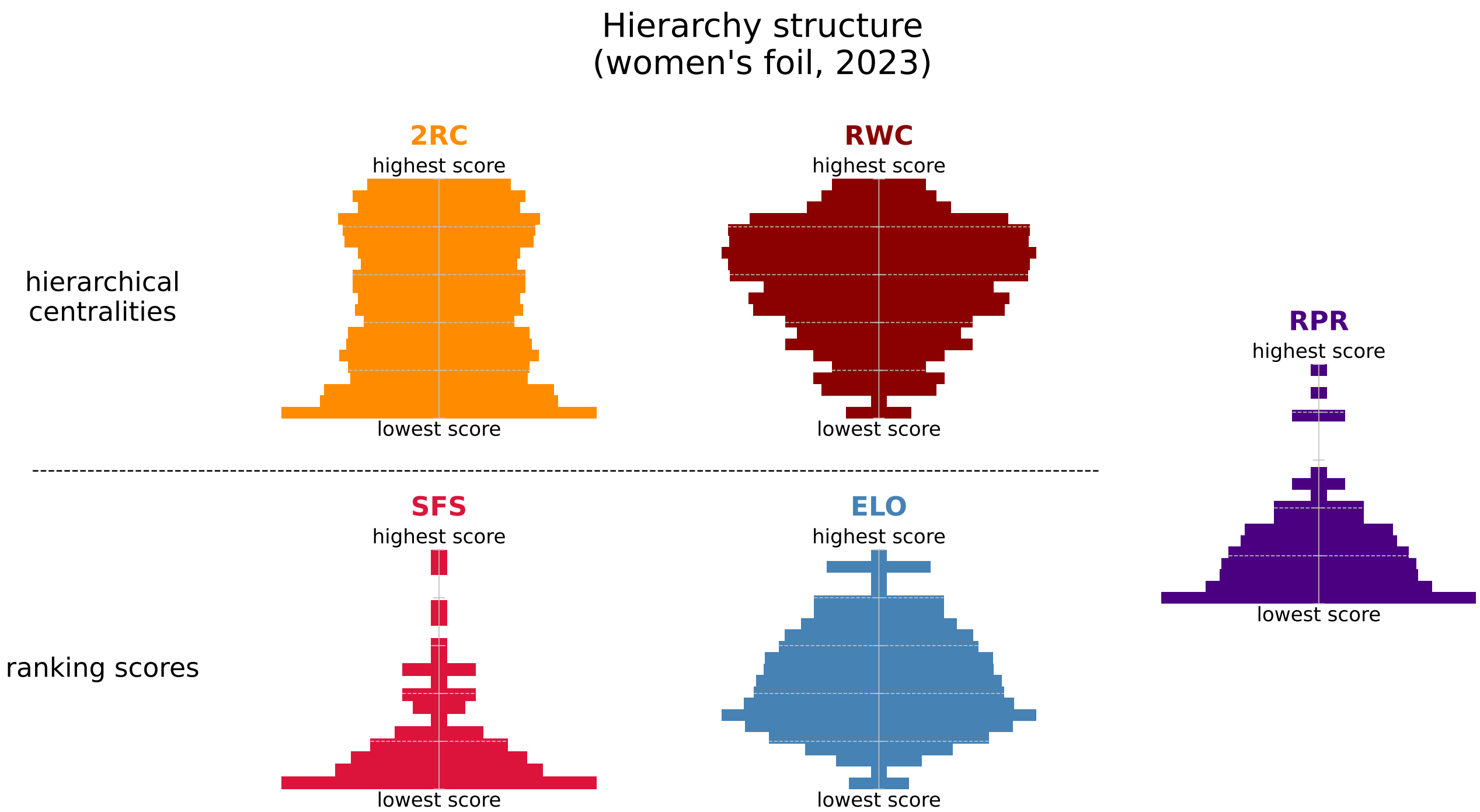


**Figure S39. The structure of the hierarchy for women's foil in 2023.** The distribution of nodes along the 2-reach centrality (orange), the random walk centrality (red), the official sport ranking (pink), the Elo score (cyan), and the revered PageRank (purple) for the network of women's foil matches played in 2023. The width of the bars represents the relative number of nodes having a specific centrality value on log-scale.

## The rank–score distribution of local measures

In Figures 6-7 of the main text, we presented the rank–socre distribution of athletes in men's tennis and men's sabre in 2015, using 5 different ranking scores. Such illustration can be done for the network of matches of any studied sport and from any year. In this section, we show the analogous results for all studied sports from the years 2015 and 2023 in Figures S40-S55.

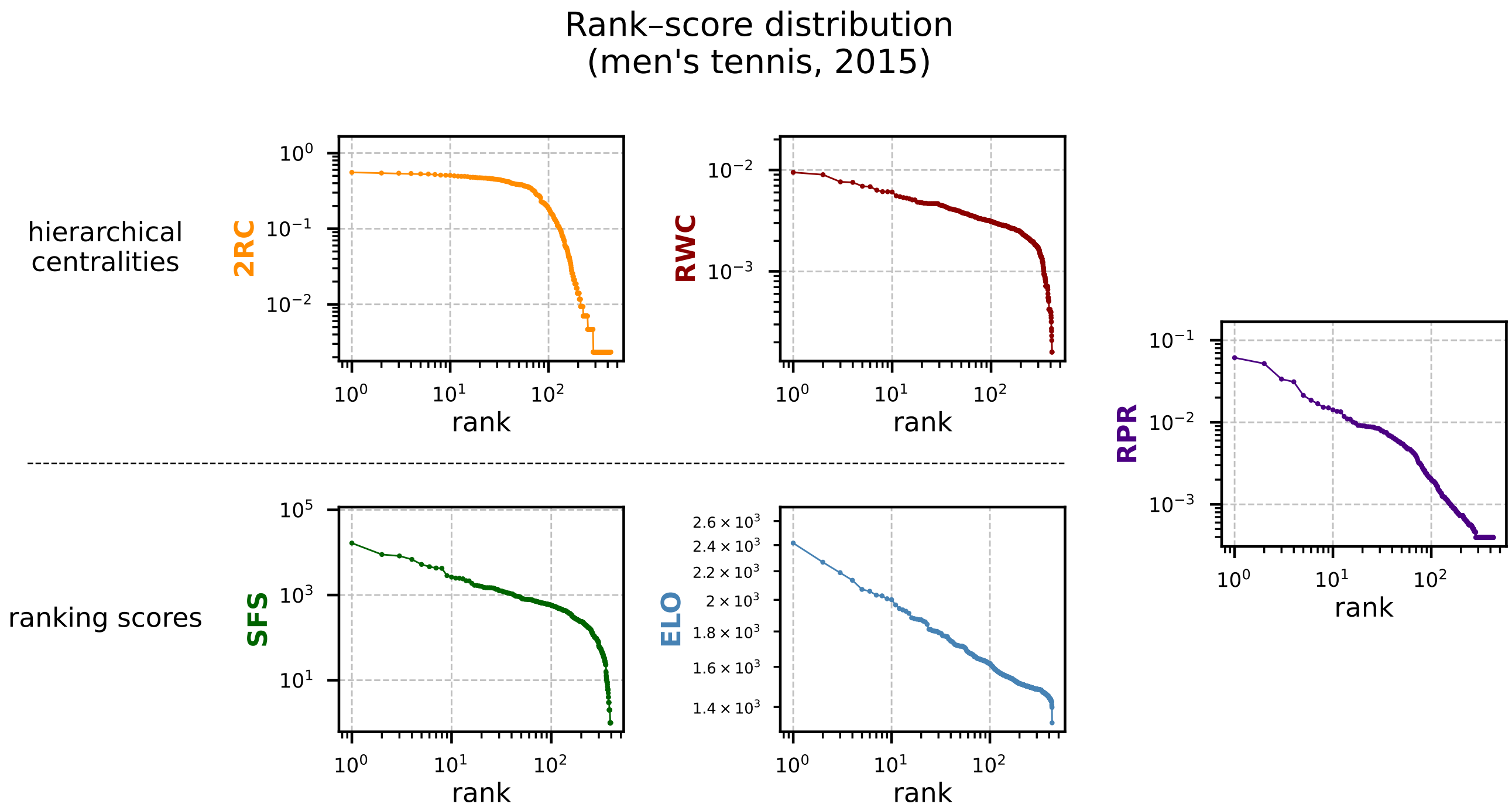


**Figure S40. The rank–score distribution of local measures for men's tennis in 2015.** The rank–score distribution of nodes along the 2-reach centrality (orange), the random walk centrality (red), the official sport ranking (pink), the Elo score (cyan), and the reverse PageRank (purple) for the network of men's tennis matches played in 2015. In the case of reversed PageRank two segments can be found following power-law scaling.

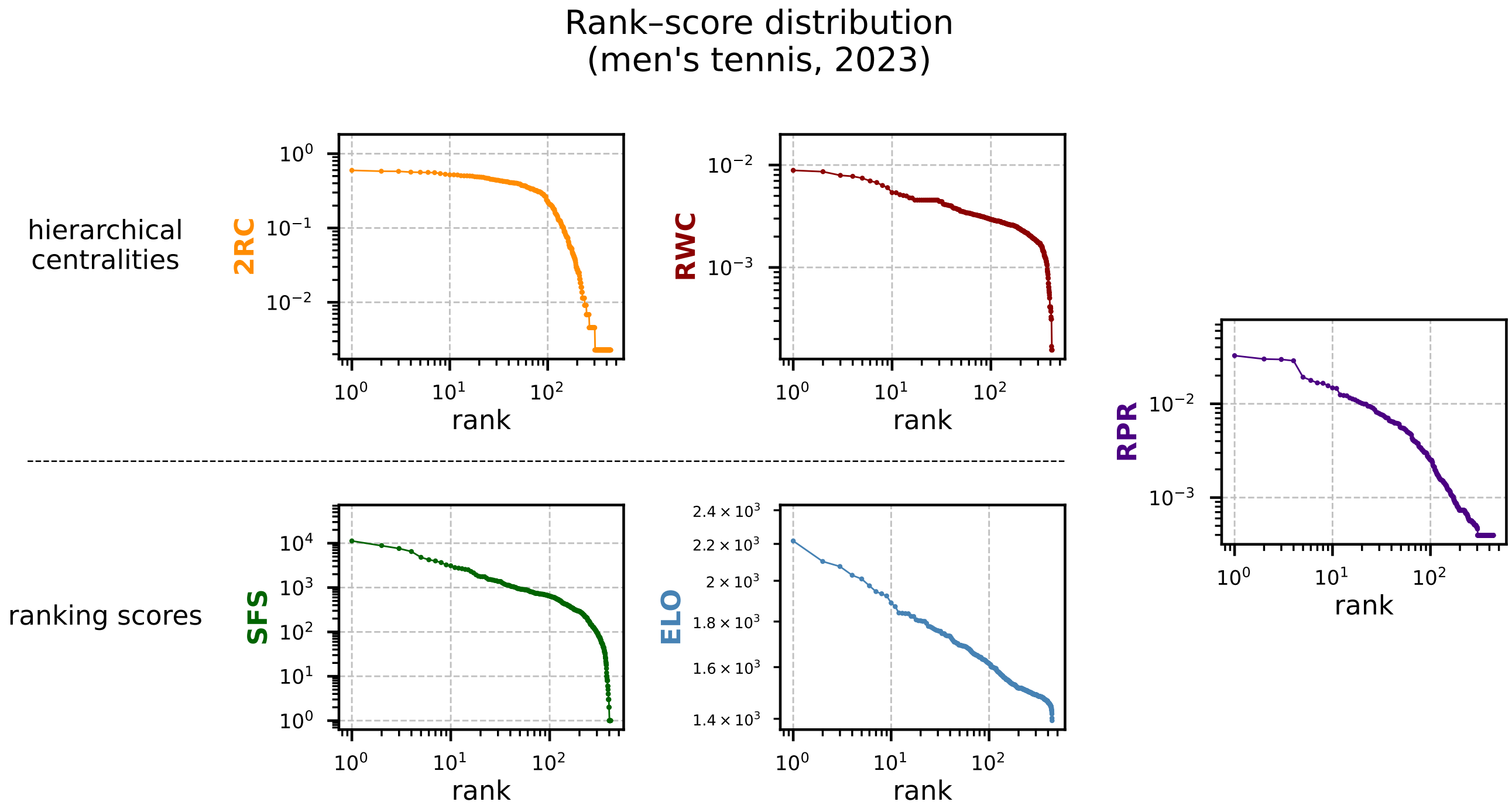


**Figure S41. The rank–score distribution of local measures for men's tennis in 2015.** The rank–score distribution of nodes along the 2-reach centrality (orange), the random walk centrality (red), the official sport ranking (pink), the Elo score (cyan), and the reverse PageRank (purple) for the network of men's tennis matches played in 2015. In the case of reversed PageRank two segments can be found following power-law scaling.

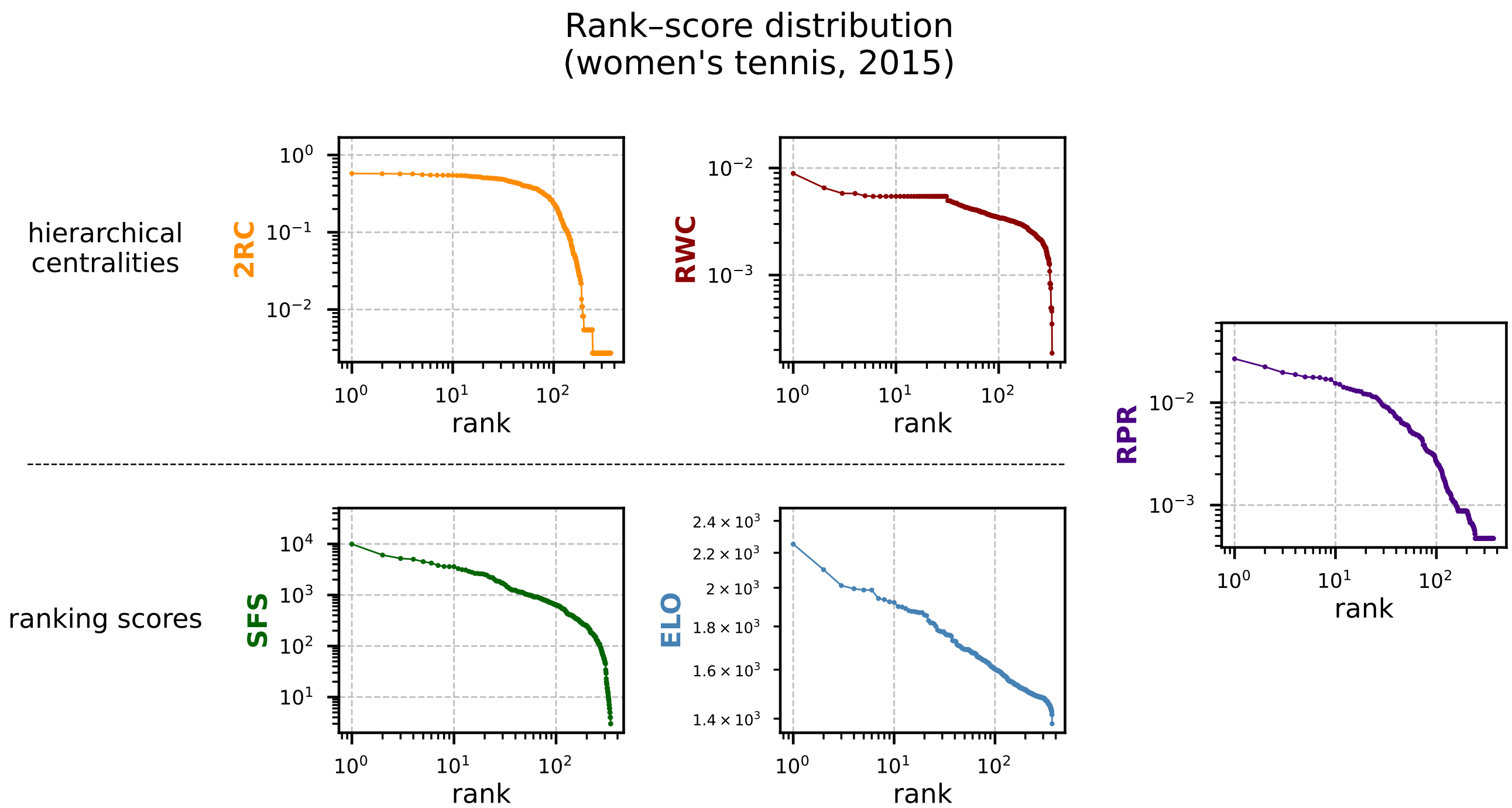


**Figure S42.** **The rank–score distribution of local measures for women's tennis in 2015.** The rank–score distribution of nodes along the 2-reach centrality (orange), the random walk centrality (red), the official sport ranking (pink), the Elo score (cyan), and the reverse PageRank (purple) for the network of women's tennis matches played in 2015. In the case of reversed PageRank two segments can be found following power-law scaling.

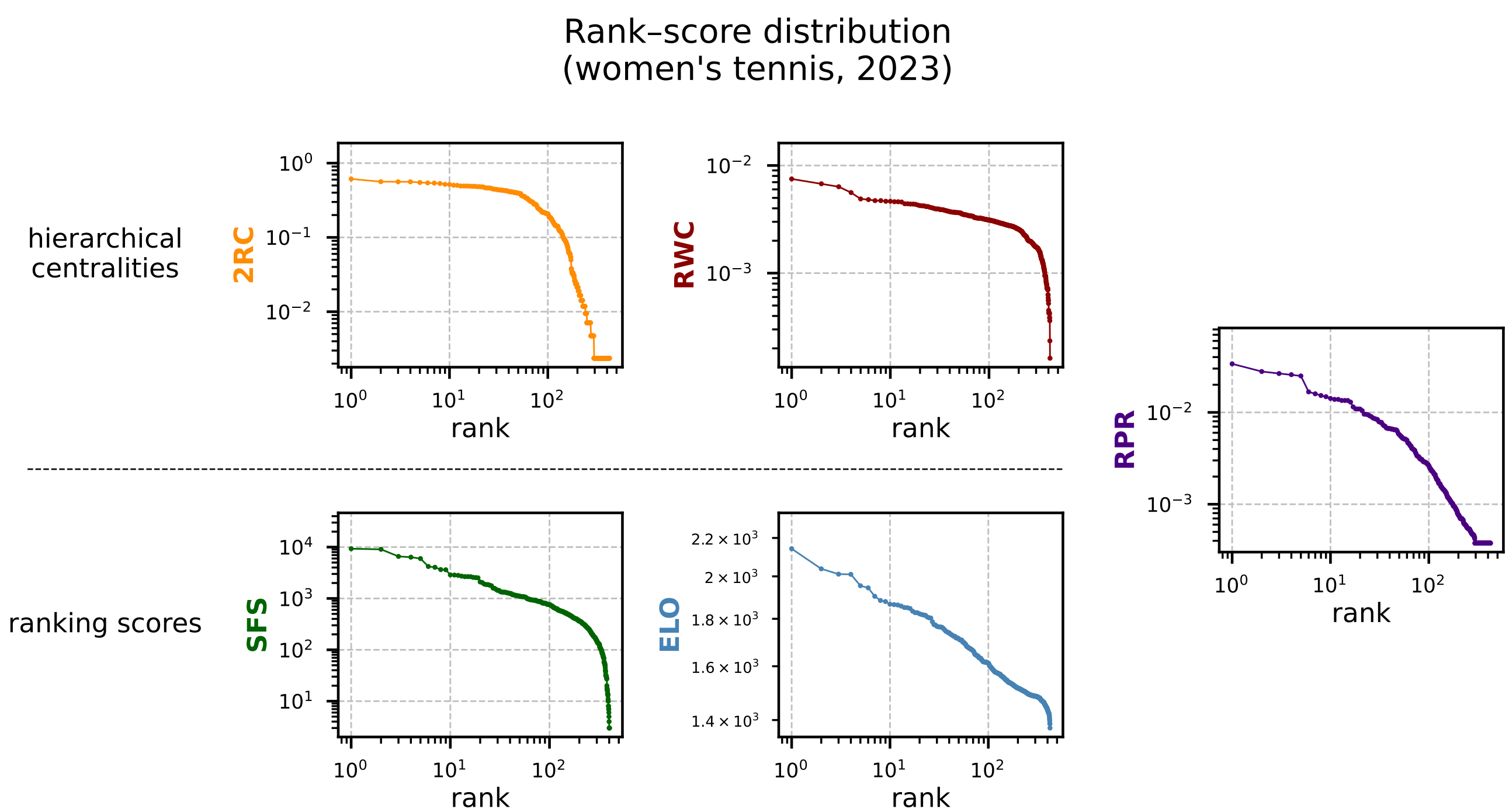


**Figure S43.** **The rank–score distribution of local measures for women's tennis in 2023.** The rank–score distribution of nodes along the 2-reach centrality (orange), the random walk centrality (red), the official sport ranking (pink), the Elo score (cyan), and the reverse PageRank (purple) for the network of women's tennis matches played in 2023. In the case of reversed PageRank two segments can be found following power-law scaling.

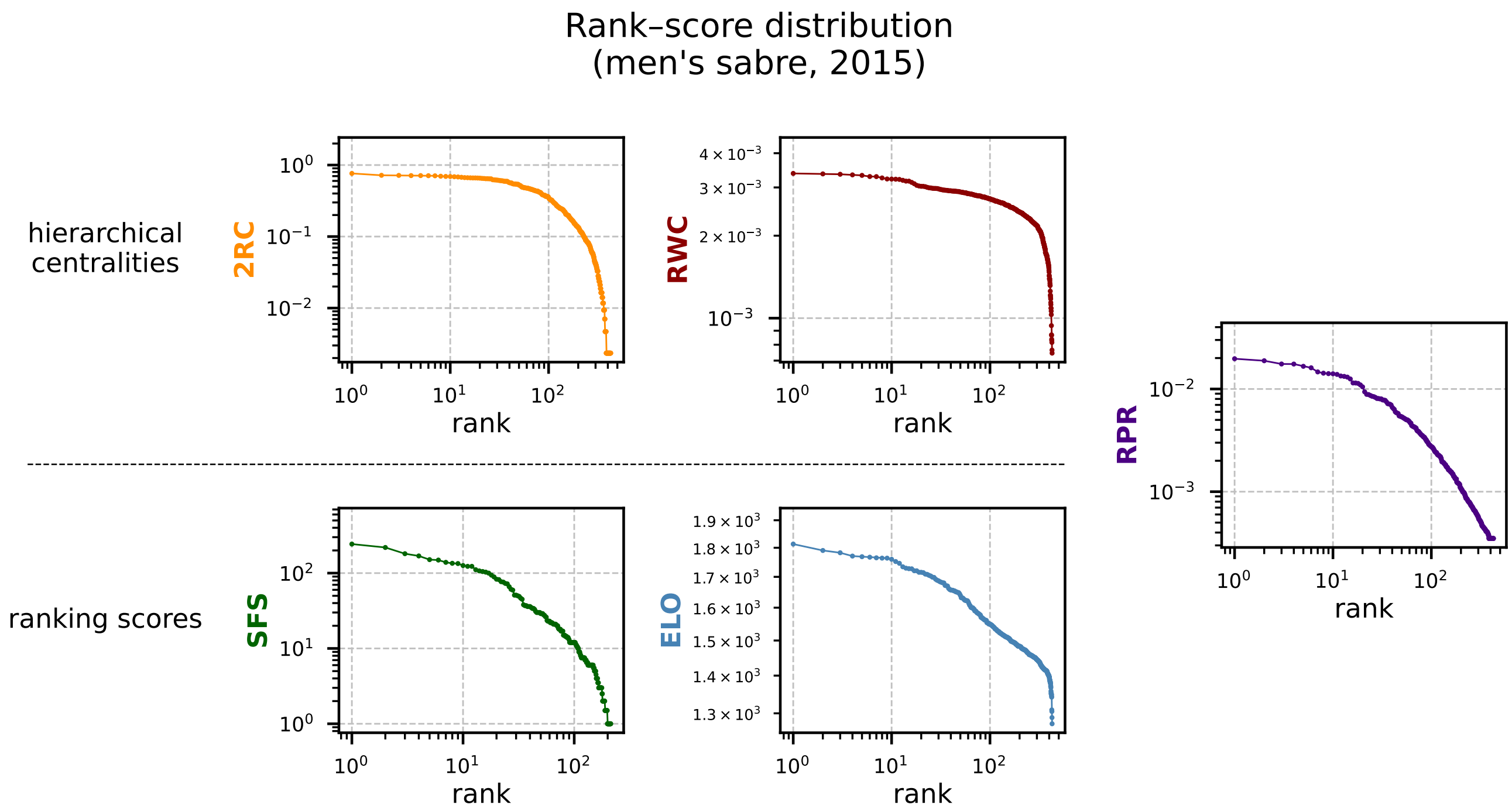


**Figure S44. The rank–score distribution of local measures for men's sabre in 2015.** The rank–score distribution of nodes along the 2-reach centrality (orange), the random walk centrality (red), the official sport ranking (pink), the Elo score (cyan), and the reverse PageRank (purple) for the network of men's sabre matches played in 2015. In the case of reversed PageRank two segments can be found following power-law scaling.

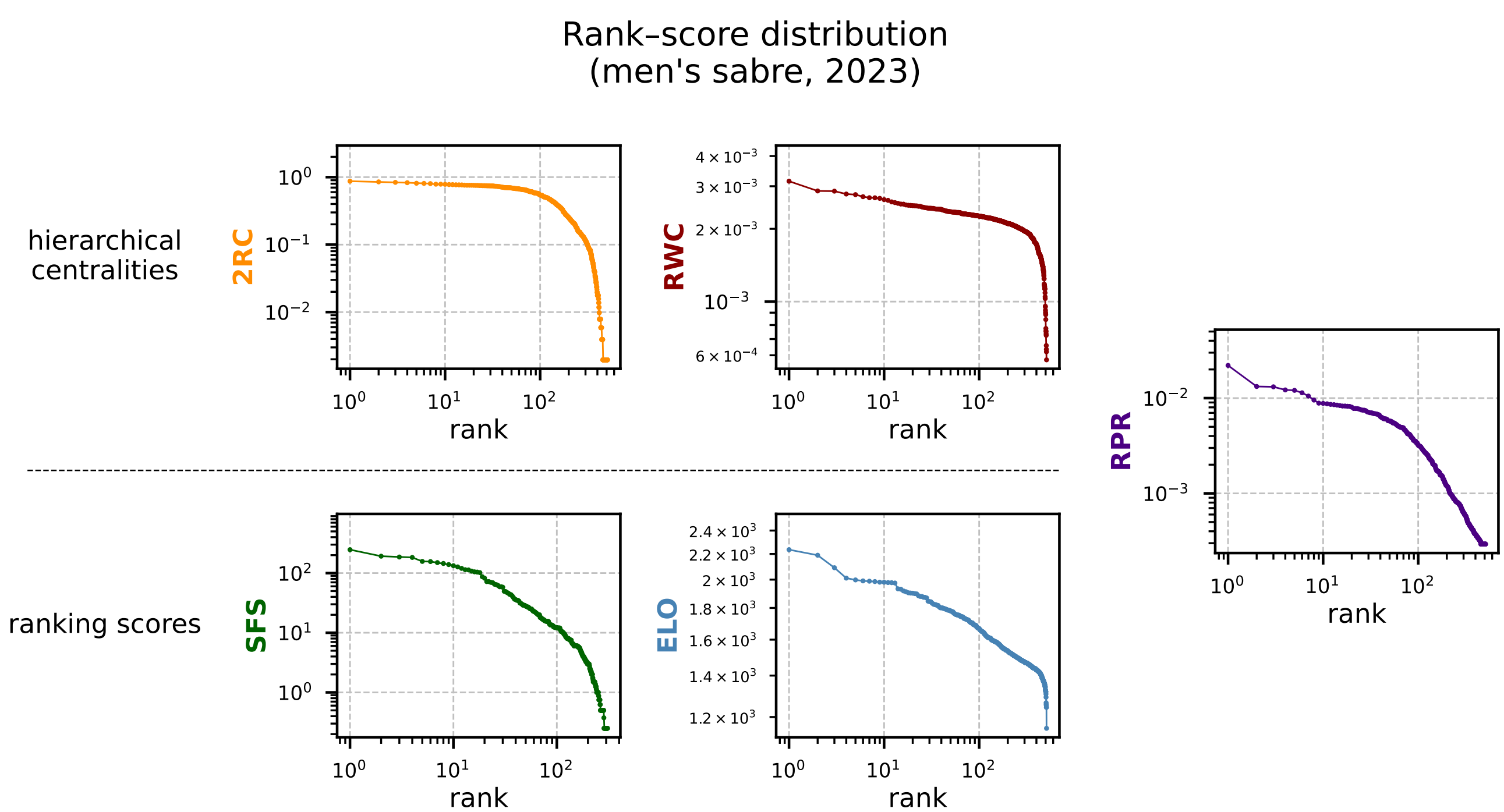


**Figure S45. The rank–score distribution of local measures for men's sabre in 2023.** The rank–score distribution of nodes along the 2-reach centrality (orange), the random walk centrality (red), the official sport ranking (pink), the Elo score (cyan), and the reverse PageRank (purple) for the network of men's sabre matches played in 2023. In the case of reversed PageRank two segments can be found following power-law scaling.

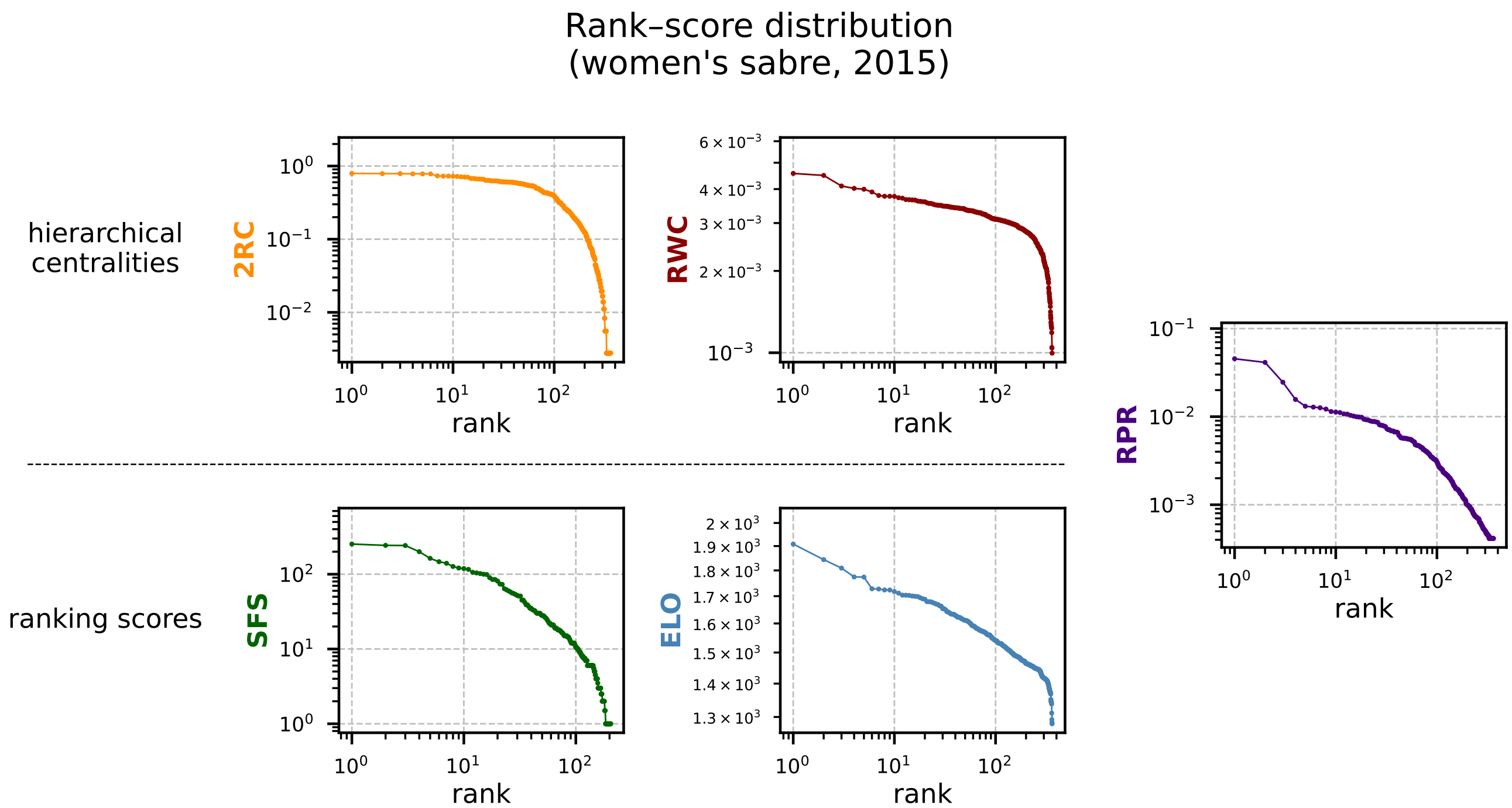


**Figure S46.** **The rank–score distribution of local measures for women's sabre in 2015.** The rank–score distribution of nodes along the 2-reach centrality (orange), the random walk centrality (red), the official sport ranking (pink), the Elo score (cyan), and the reverse PageRank (purple) for the network of women's sabre matches played in 2015. In the case of reversed PageRank two segments can be found following power-law scaling.

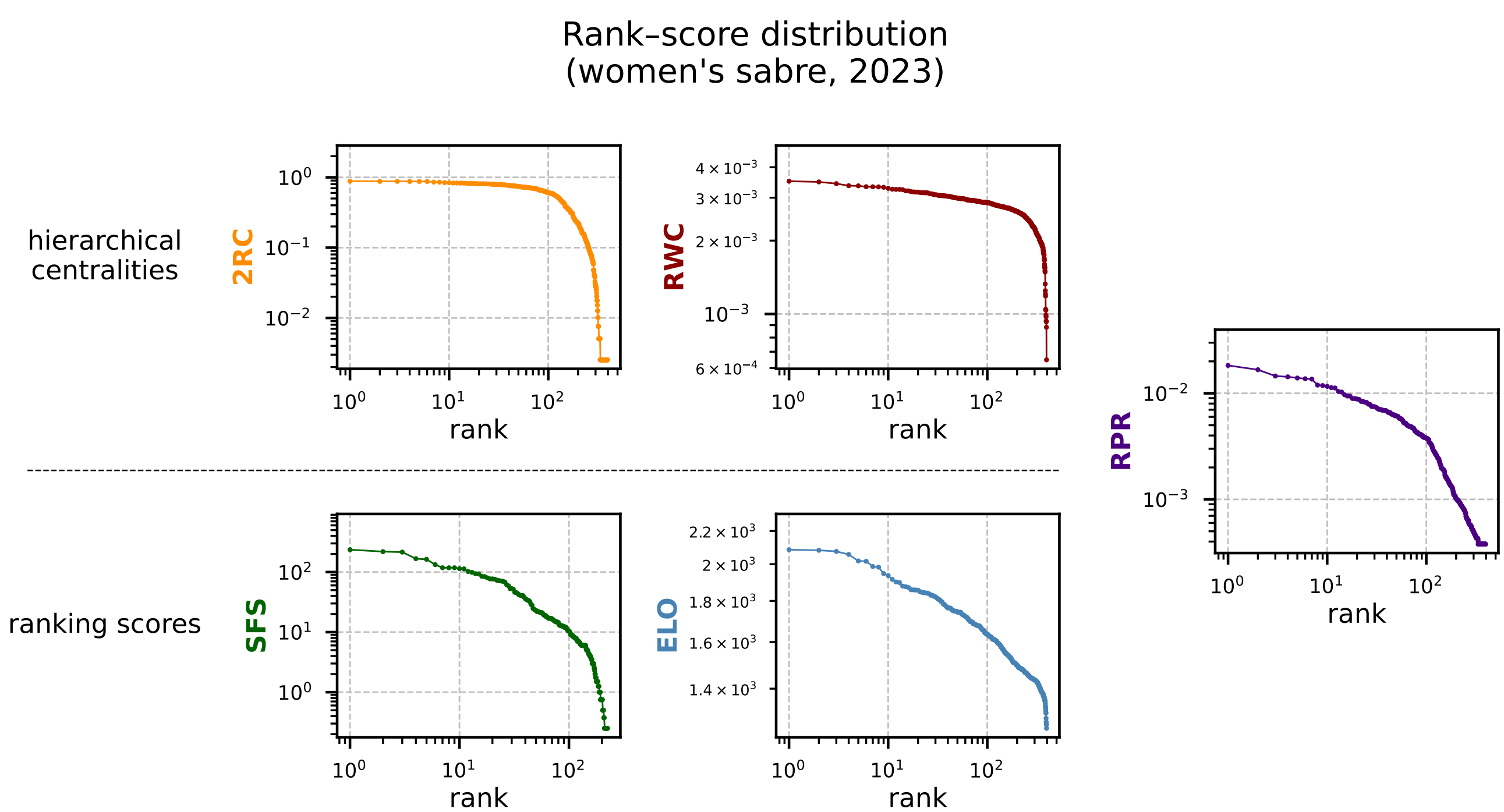


**Figure S47.** **The rank–score distribution of local measures for women's sabre in 2023.** The rank–score distribution of nodes along the 2-reach centrality (orange), the random walk centrality (red), the official sport ranking (pink), the Elo score (cyan), and the reverse PageRank (purple) for the network of women's sabre matches played in 2023. In the case of reversed PageRank two segments can be found following power-law scaling.

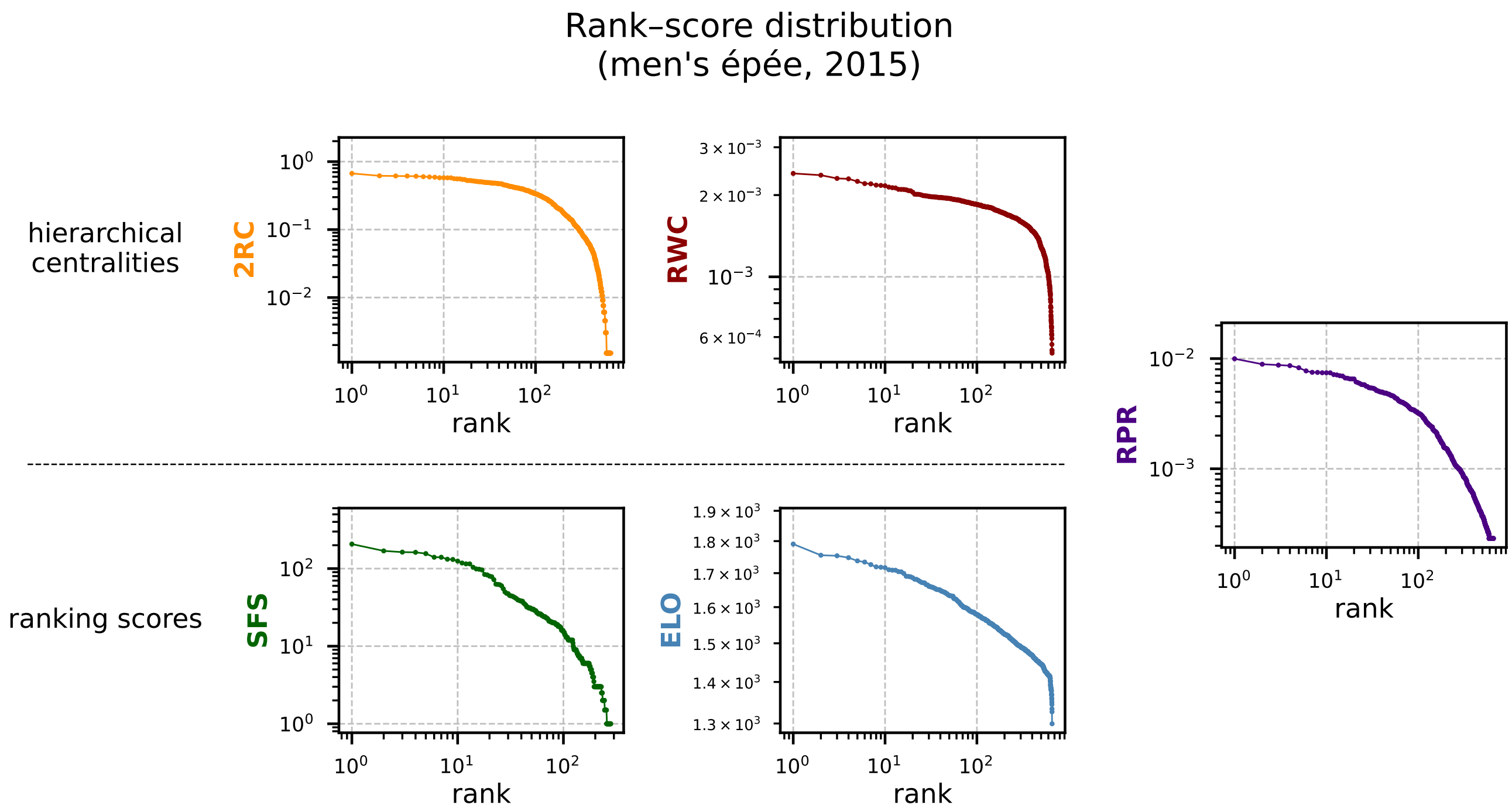


**Figure S48. The rank–score distribution of local measures for men's épée in 2015.** The rank–score distribution of nodes along the 2-reach centrality (orange), the random walk centrality (red), the official sport ranking (pink), the Elo score (cyan), and the reverse PageRank (purple) for the network of men's épée matches played in 2015. In the case of reversed PageRank two segments can be found following power-law scaling.

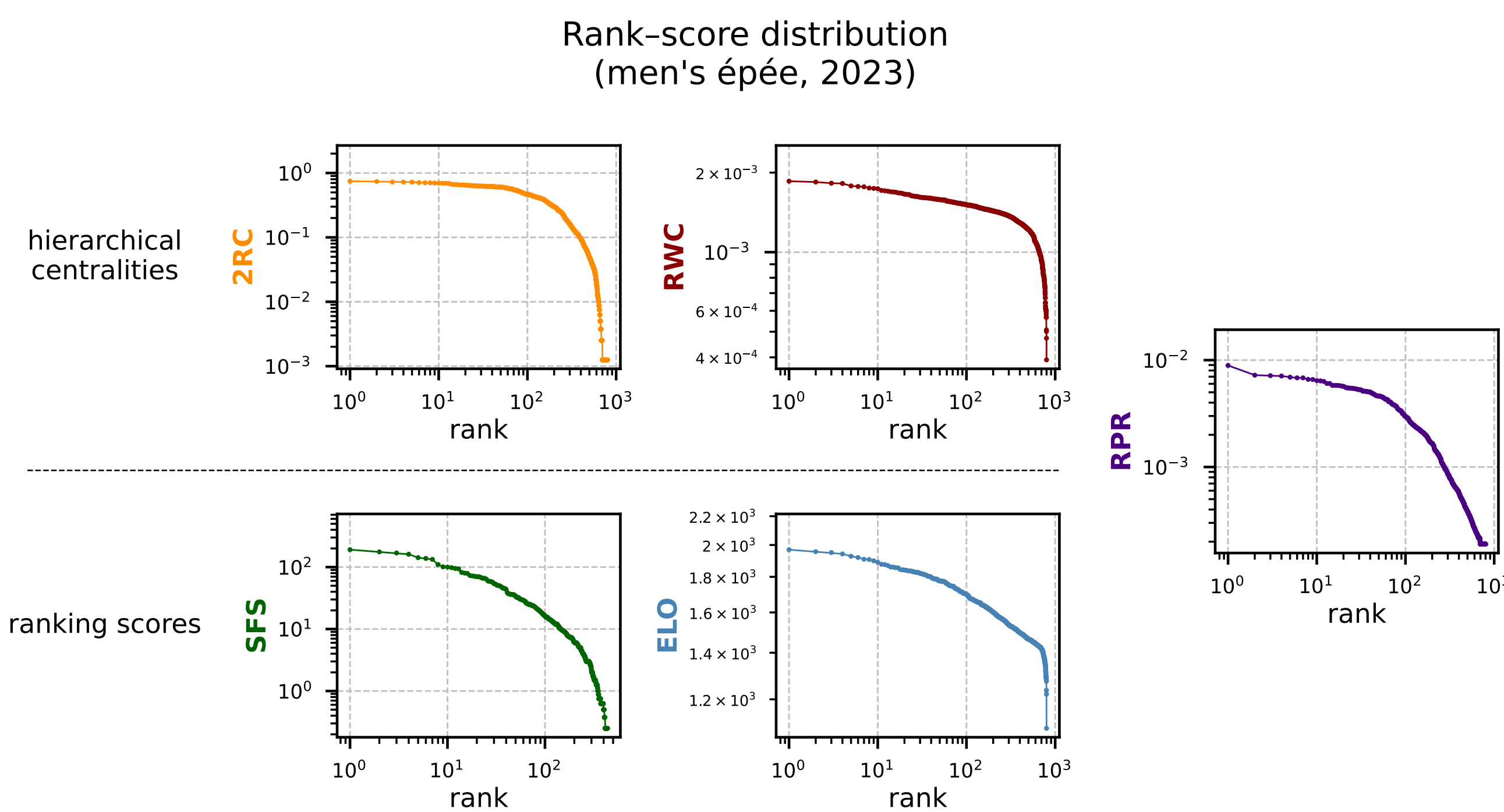


**Figure S49. The rank–score distribution of local measures for men's épée in 2023.** The rank–score distribution of nodes along the 2-reach centrality (orange), the random walk centrality (red), the official sport ranking (pink), the Elo score (cyan), and the reverse PageRank (purple) for the network of men's épée matches played in 2023. In the case of reversed PageRank two segments can be found following power-law scaling.

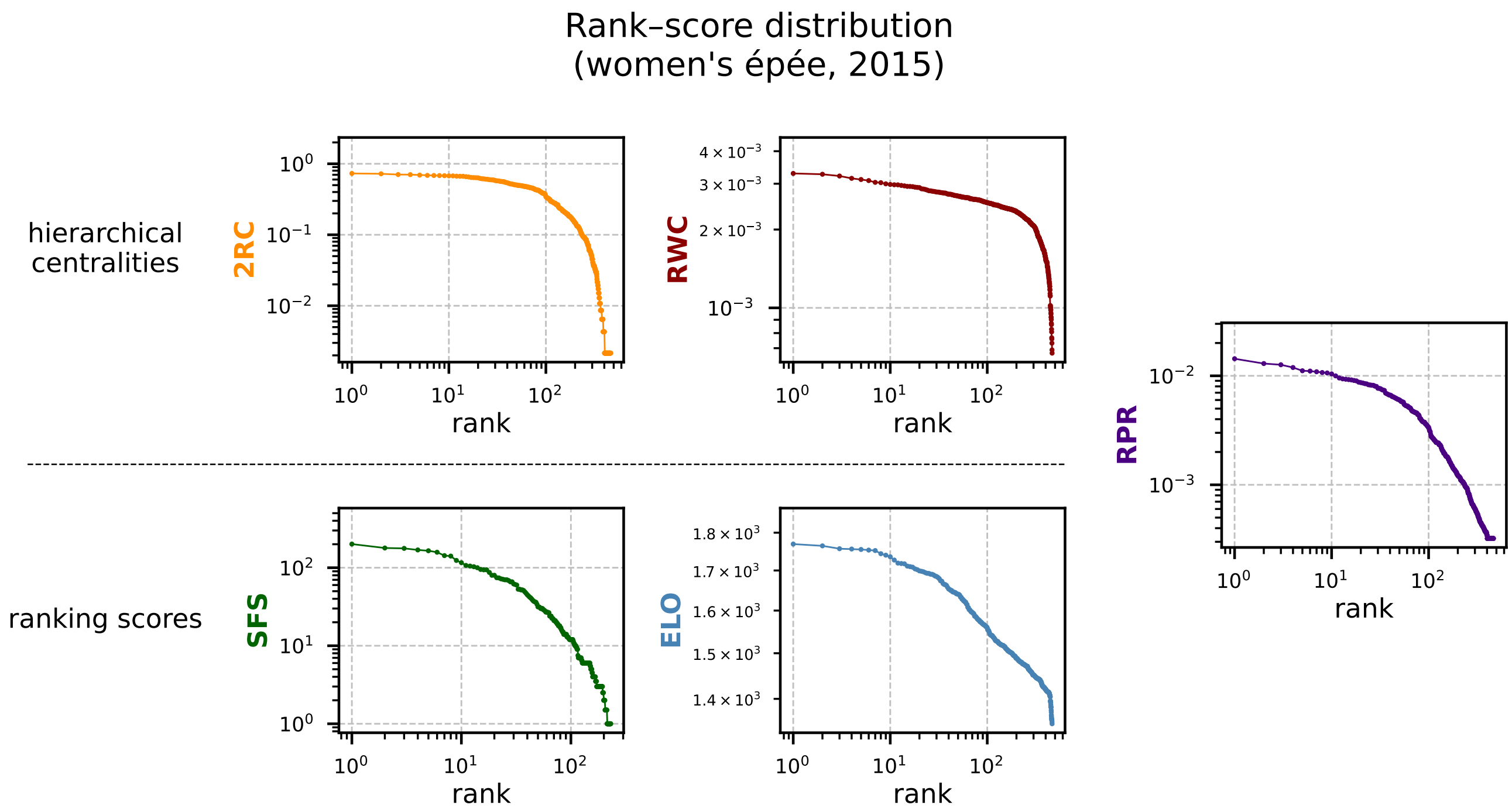


**Figure S50.** **The rank–score distribution of local measures for women's épée in 2015.** The rank–score distribution of nodes along the 2-reach centrality (orange), the random walk centrality (red), the official sport ranking (pink), the Elo score (cyan), and the reverse PageRank (purple) for the network of women's épée matches played in 2015. In the case of reversed PageRank two segments can be found following power-law scaling.

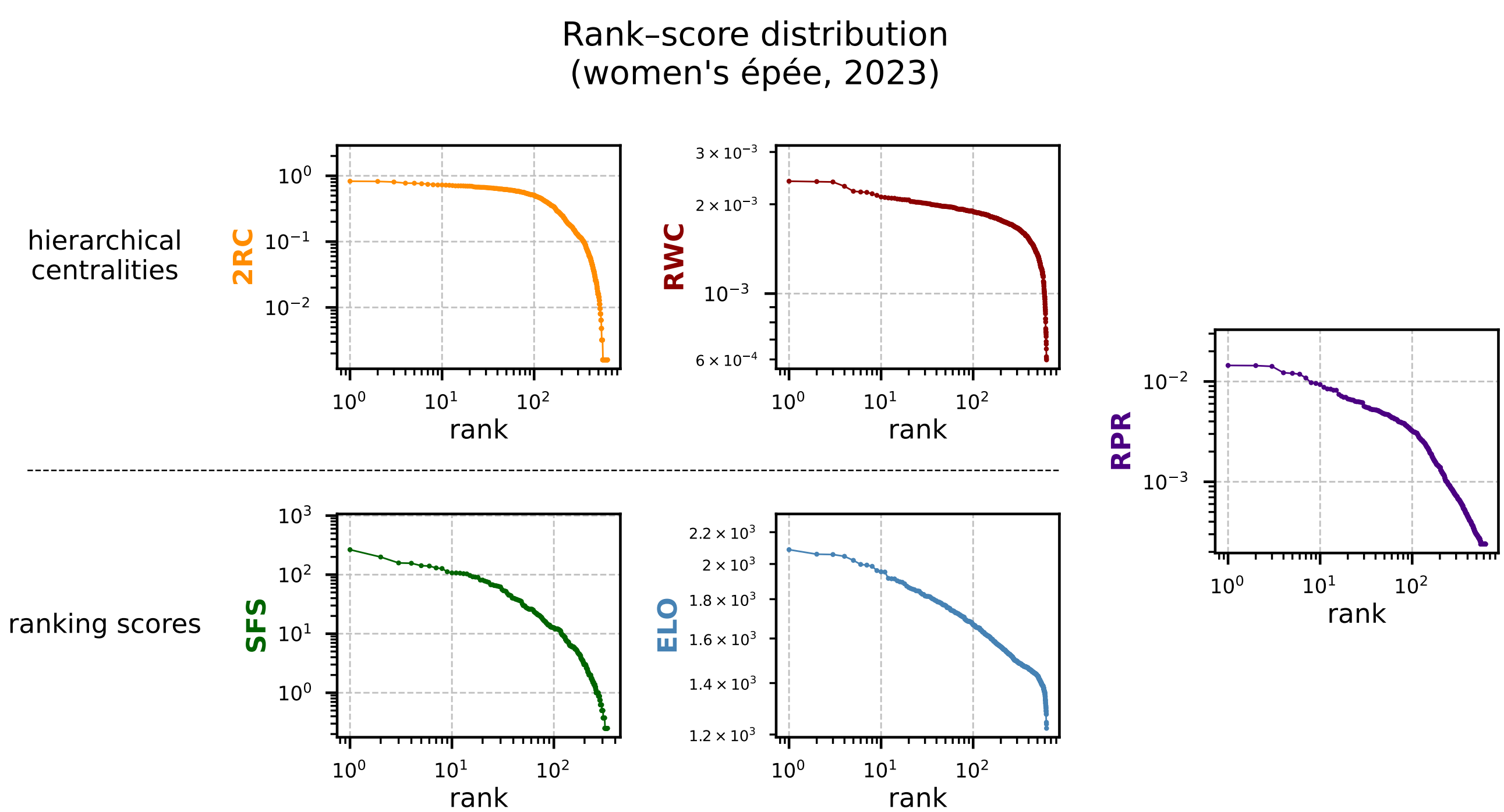


**Figure S51.** **The rank–score distribution of local measures for women's épée in 2023.** The rank–score distribution of nodes along the 2-reach centrality (orange), the random walk centrality (red), the official sport ranking (pink), the Elo score (cyan), and the reverse PageRank (purple) for the network of women's épée matches played in 2023. In the case of reversed PageRank two segments can be found following power-law scaling.

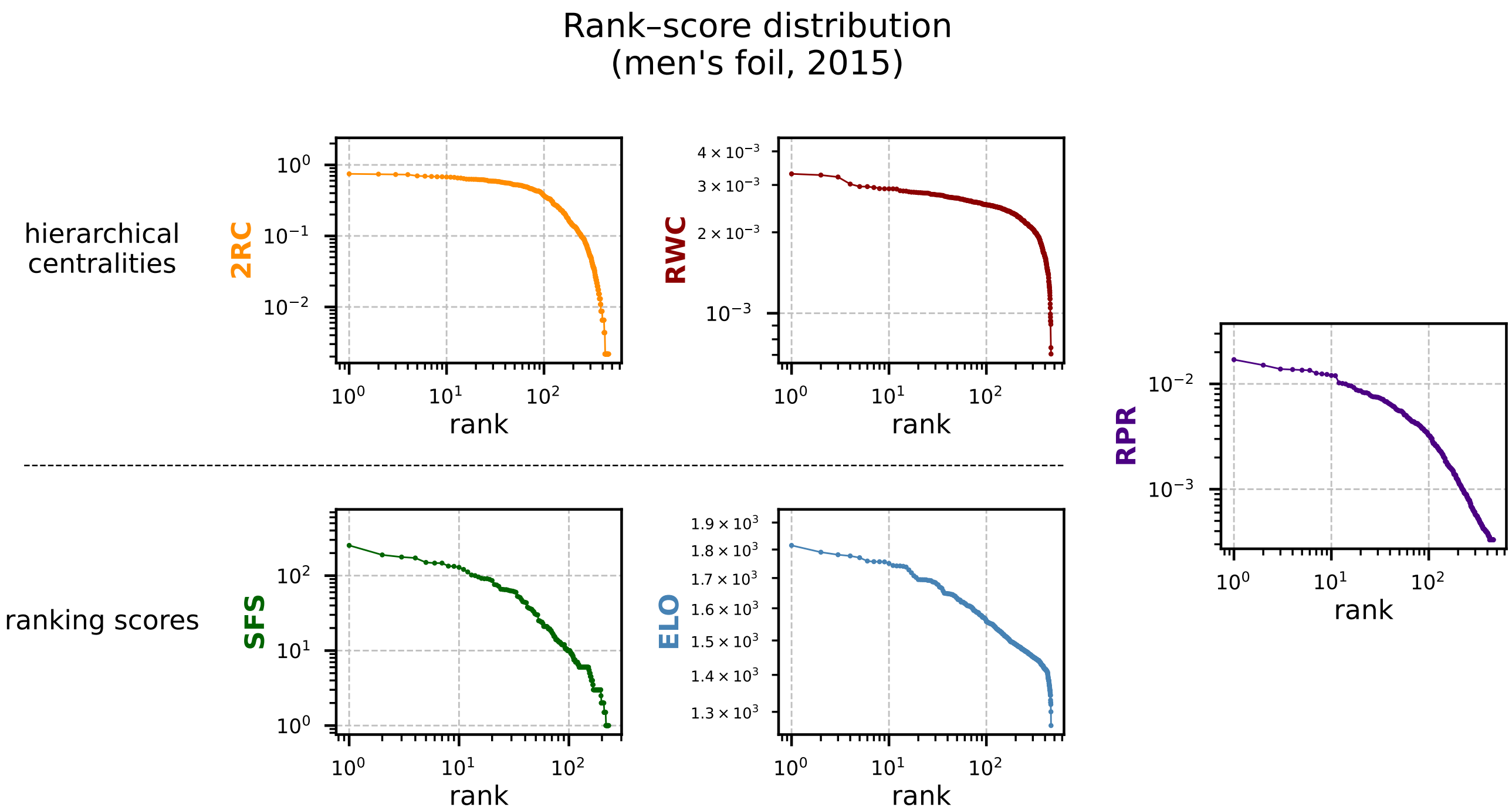


**Figure S52. The rank–score distribution of local measures for men's foil in 2015.** The rank–score distribution of nodes along the 2-reach centrality (orange), the random walk centrality (red), the official sport ranking (pink), the Elo score (cyan), and the reverse PageRank (purple) for the network of men's foil matches played in 2015. In the case of reversed PageRank two segments can be found following power-law scaling.

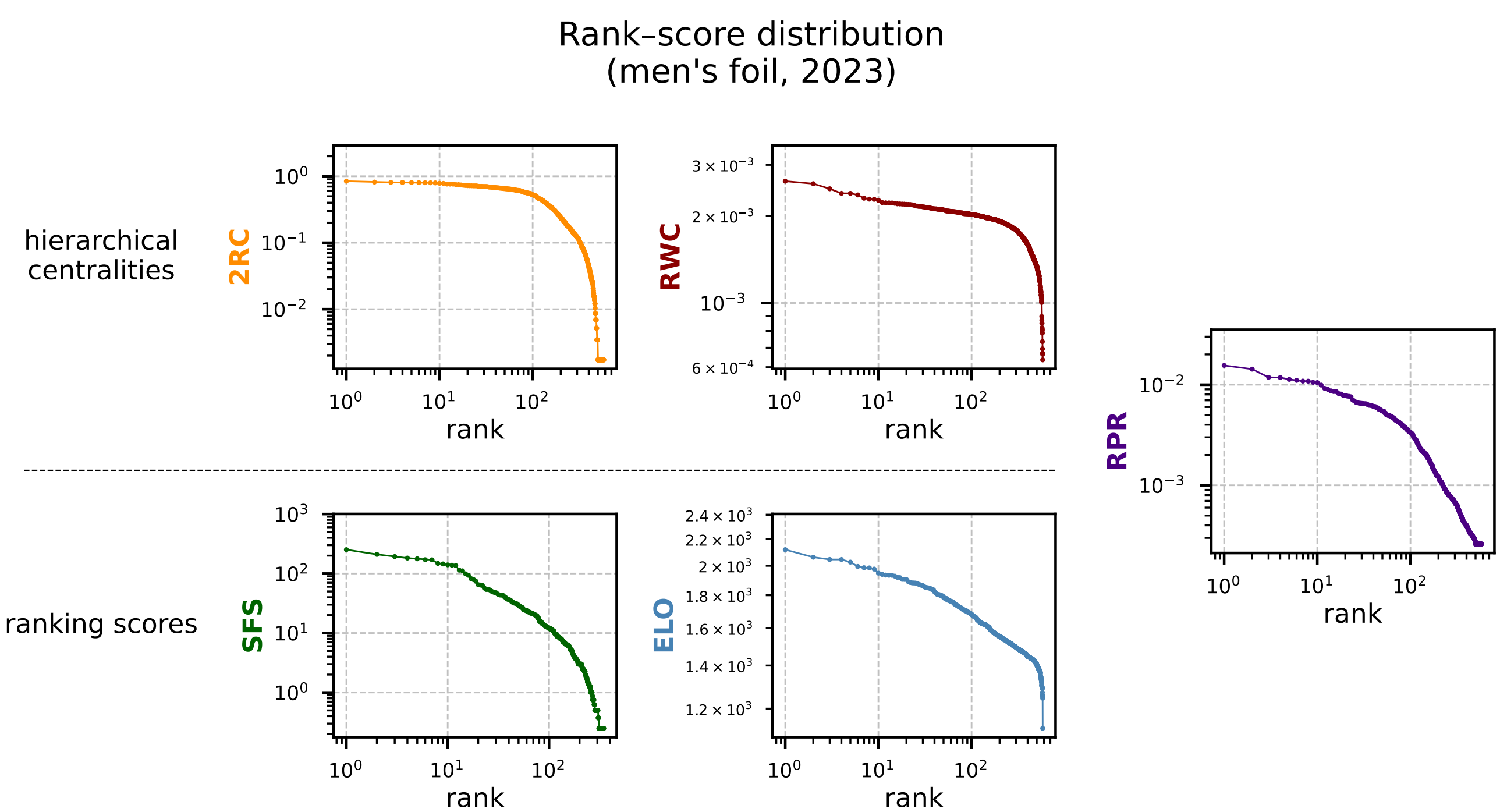


**Figure S53. The rank–score distribution of local measures for men's foil in 2023.** The rank–score distribution of nodes along the 2-reach centrality (orange), the random walk centrality (red), the official sport ranking (pink), the Elo score (cyan), and the reverse PageRank (purple) for the network of men's foil matches played in 2023. In the case of reversed PageRank two segments can be found following power-law scaling.

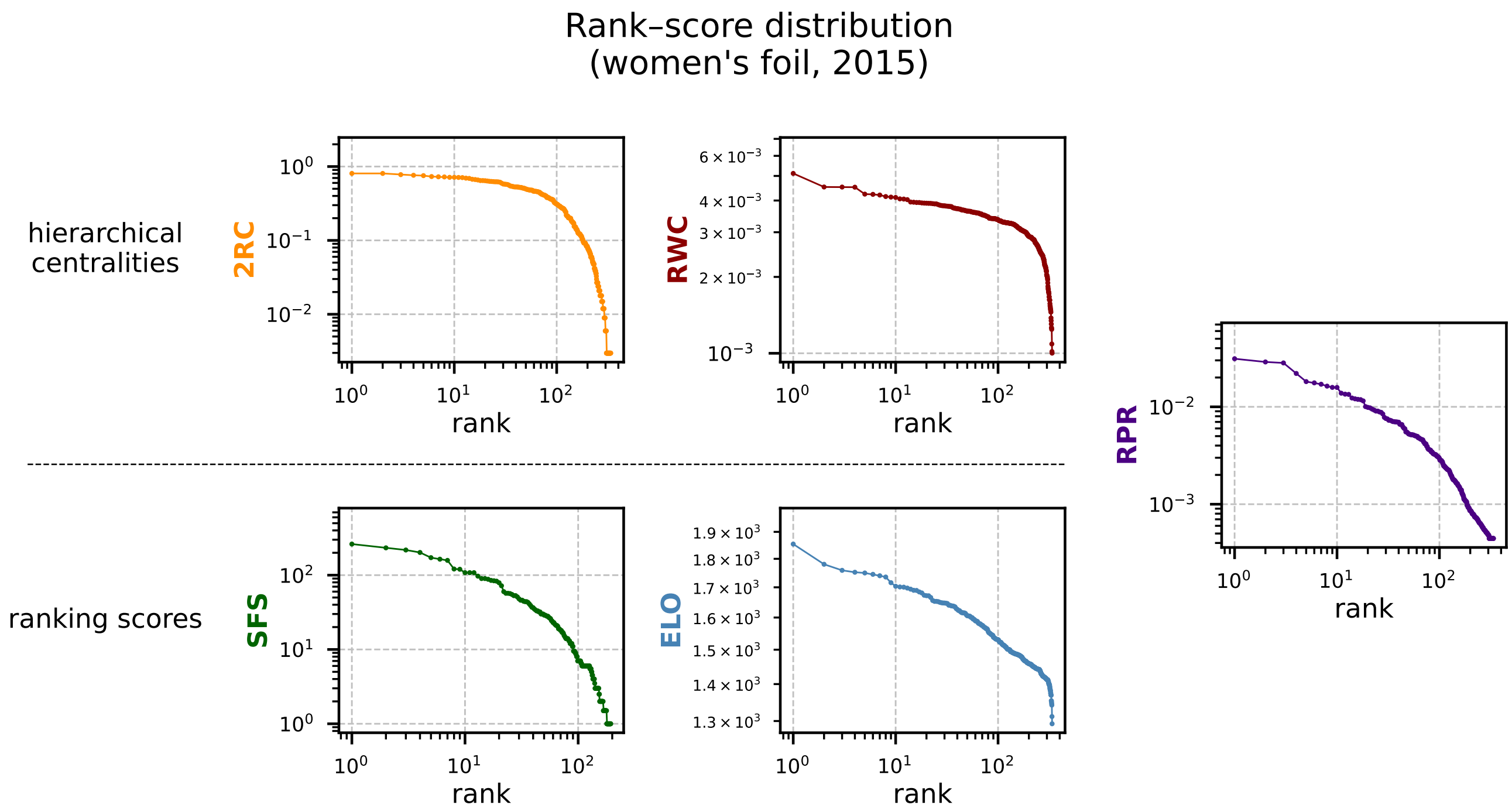


**Figure S54. The rank–score distribution of local measures for women's foil in 2015.** The rank–score distribution of nodes along the 2-reach centrality (orange), the random walk centrality (red), the official sport ranking (pink), the Elo score (cyan), and the reverse PageRank (purple) for the network of women's foil matches played in 2015. In the case of reversed PageRank two segments can be found following power-law scaling.

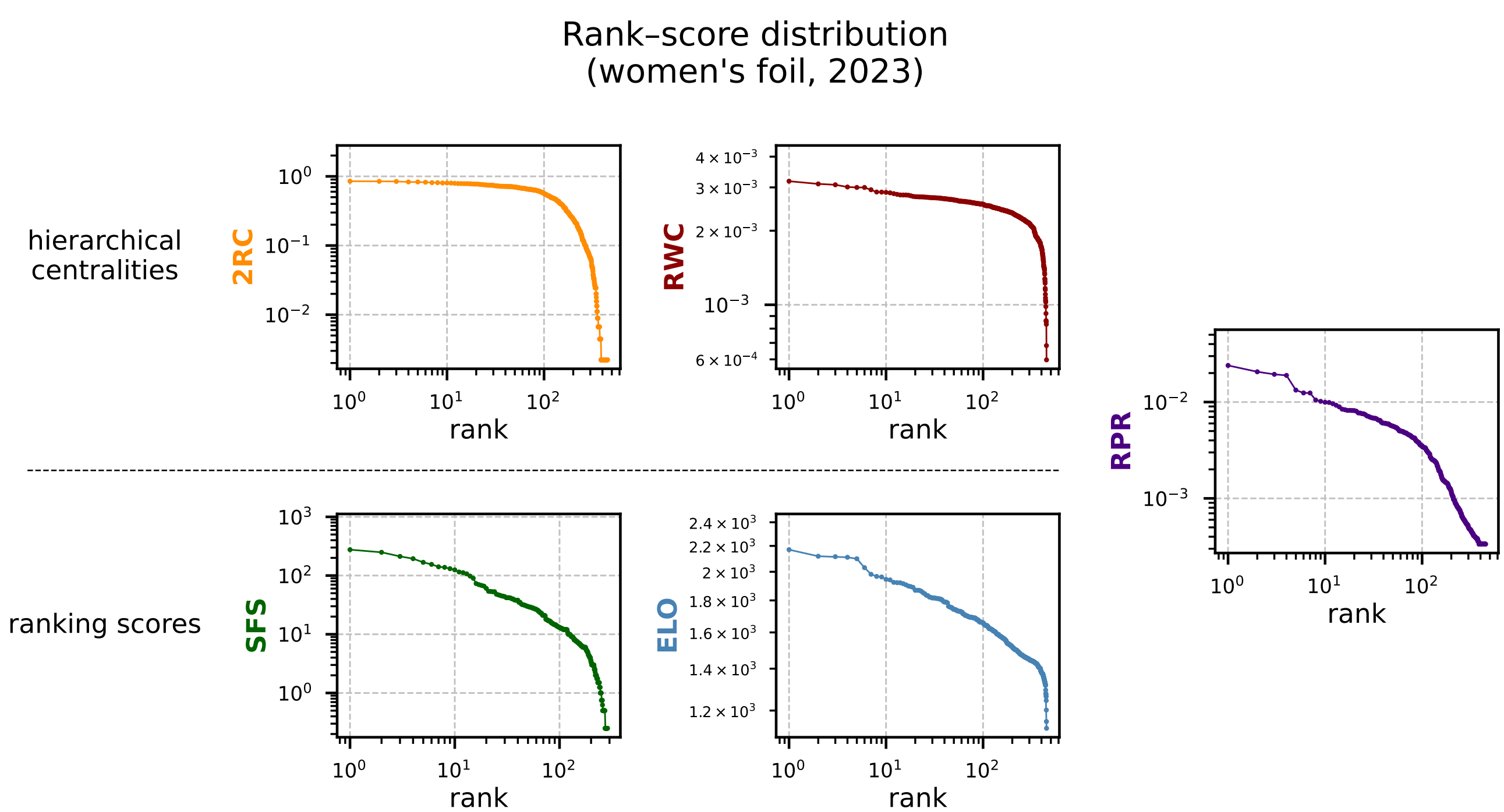


**Figure S55. The rank–score distribution of local measures for women's foil in 2023.** The rank–score distribution of nodes along the 2-reach centrality (orange), the random walk centrality (red), the official sport ranking (pink), the Elo score (cyan), and the reverse PageRank (purple) for the network of women's foil matches played in 2023. In the case of reversed PageRank two segments can be found following power-law scaling.

## Calculating Elo scores on a limited set of matches

Since Elo rating is not tracked officially in most sports, we calculated it from the datasets listed in Table 1. However, since these datasets contain only the elite events of each sports category, the scores we got will not be the real Elo scores of players, since Elo's method must take all previous matches of a player into account, and many players played a part of their matches on lower levels. Hence, our calculation underestimates the rating of weaker players, as many of their probable victories are ignored, and this would affect the ranking of their direct opponents too, since their score also depends on the scores of their opponents, and so on.

To estimate how much impact this lack of data has on our analysis, we tried out how different Elo ratings we would get using an even more limited set of matches. We have filtered our datasets containing elite sports events and kept only the most prestigious tournaments. In the case of tennis, we kept the Grand Slams, the Davis Cup, the season-ending finals, the challengers, and the Masters 1000 tournaments, while in the case of fencing, we kept the Olympics, the World Championships, the Grand Prix, and the continental championships. Table S1 shows how many matches remained in these new datasets. We have calculated the Elo ratings on the filtered data and compared the scores of the remaining players to their original scores gained from the original datasets in each year. We found that the average relative difference is smaller than 4% for all sports categories, and 95% of scores differ less than 10% from the original (with the exceptions of women's tennis and épée cases). The details of these difference statistics are shown in Table S1 for all categories. A more thorough examination also sheds light on the fact that the largest differences are in the cases of players who play only a few matches in the limited datasets. We can hence conclude that considering only a narrower set of matches, which is filtered by the level of competition, does not influence the Elo scores of the most important players substantially, so this does not invalidate the presented results in the main text.

Additionally, to exclude the possibility that these small differences are an artefact of both datasets containing random data and players having scores that fluctuate around the starting value, we compared the results of Table S1 to a naive benchmark. If the calculated Elo scores based on both datasets were in the proximity of the starting value, then making them equal to their average would have about the same effect as recalculating them. Hence, we counted the average and the 95% quantile of the relative difference in the case where the remaining players' Elo ratings were substituted by the yearly average of all player's Elo scores, and showed these values in Table S2. We found that all of these quantities are at least twice as large as their equivalent in Table S1 (again, with the exception of the women's tennis case, but the value is still noticeably larger there). This proves that the difference among Elo rating scores is not the artefact of the fluctuating nature of Elo's method, and limiting the datasets indeed affects the values to some small extent.

| Sport | Discipline | Sex | Number of original matches | Number of filtered matches | average relative difference | 95% quantile of relative differences |
|---|---|---|---|---|---|---|
| Tennis | | Men | 194,996 | 67,224 | 0.018 | 0.067 |
| | | Women | 158,092 | 37,937 | 0.027 | 0.103 |
| Fencing | Foil | Men | 54,629 | 21,556 | 0.024 | 0.080 |
| | | Women | 43,798 | 17,108 | 0.027 | 0.085 |
| | Épée | Men | 73,068 | 12,589 | 0.031 | 0.097 |
| | | Women | 55,290 | 9,648 | 0.034 | 0.102 |
| | Sabre | Men | 49,458 | 15,259 | 0.033 | 0.098 |
| | | Women | 43,571 | 13,816 | 0.033 | 0.095 |

**Table S1.** The average, and the 95% quantile of the relative difference between the Elo scores calculated based on the original and the limited datasets.

| Sport | Discipline | Sex | average relative difference in benchmark | 95% quantile of relative differences in benchmark |
|---|---|---|---|---|
| Tennis | | Men | 0.036 | 0.115 |
| | | Women | 0.045 | 0.157 |
| Fencing | Foil | Men | 0.069 | 0.202 |
| | | Women | 0.075 | 0.214 |
| | Épée | Men | 0.068 | 0.185 |
| | | Women | 0.074 | 0.219 |
| | Sabre | Men | 0.076 | 0.228 |
| | | Women | 0.075 | 0.214 |

**Table S2.** The average, and the 95% quantile of the relative difference between the Elo scores calculated based on the original datasets, and the naive benchmark where Elo scores are substituted by the yearly average of all player.

## Time dependence of the prediction parameters

While predicting sports matches, we fit model parameters $\hat{\alpha}$ and $\hat{\beta}$ from equation (2). In each year $t$, the parameter values are fitted for data in the fitting period, which is the $t_f$-long subsequent window before $t$. Hence, the values $\hat{\alpha}(t)$ and $\hat{\beta}(t)$ depends on the time. In Figures S56-S57, we plotted the values of these model parameters.

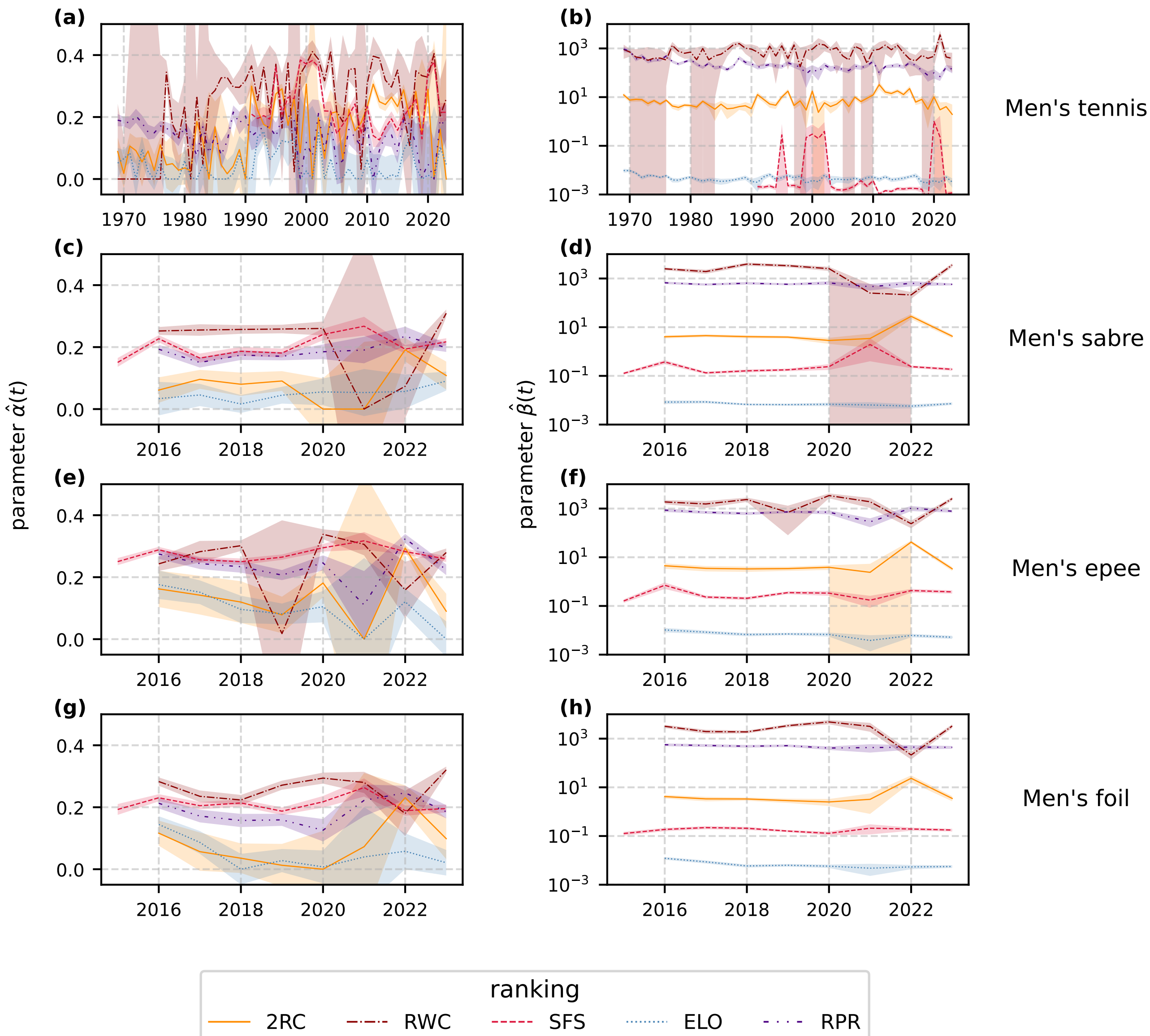


**Figure S56. Time dependence of the fitted prediction parameters in case of men's sports disciplines.** In panels (a), (c), (e), and (g), the parameter $\hat{\alpha}(t)$ i.e., the probability of an extreme upset win is shown. In panels (b), (d), (f), and (h), the parameter $\hat{\beta}(t)$ i.e., the depth of the competition is shown.

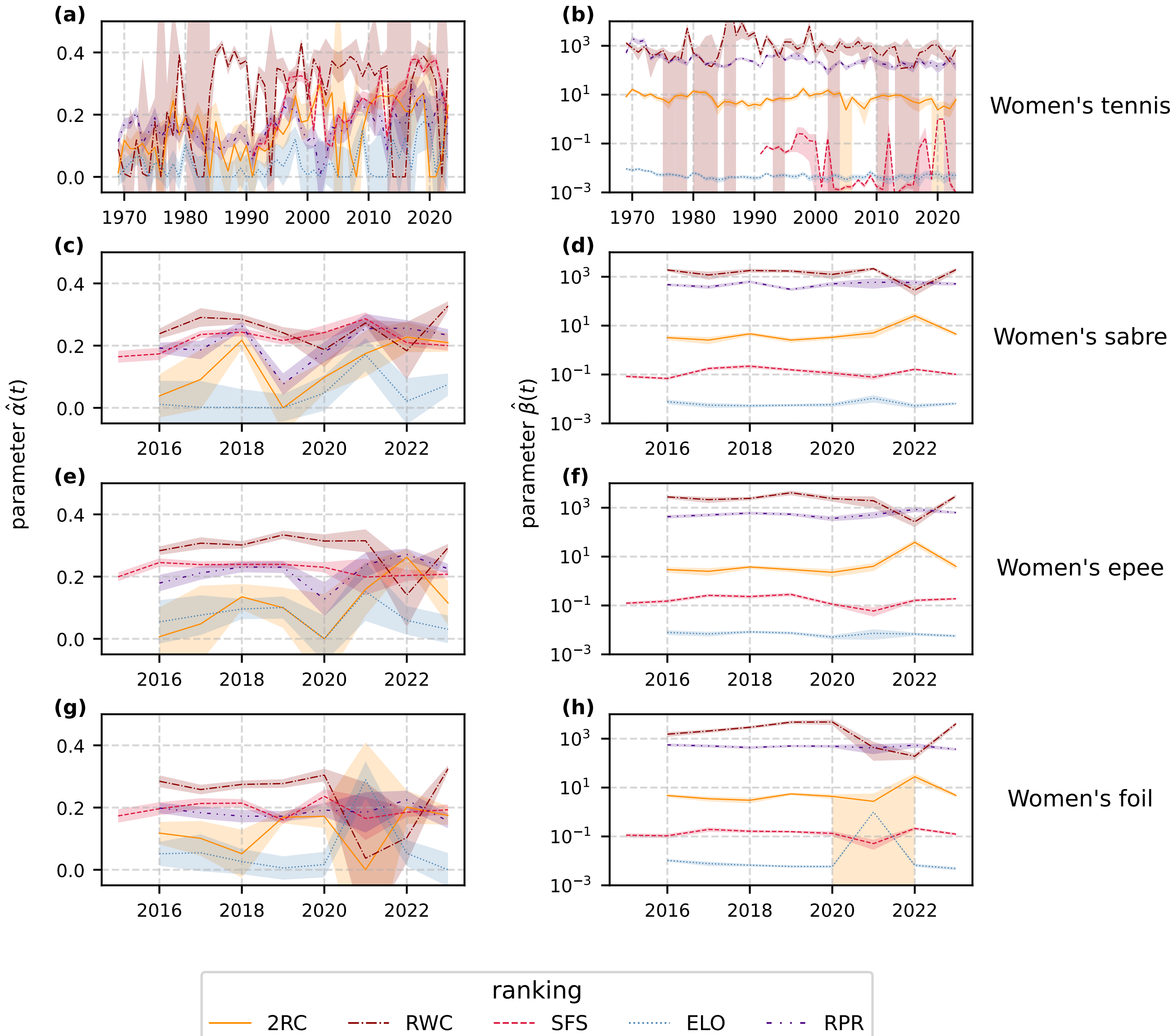


**Figure S57. Time dependence of the fitted prediction parameters in case of women's sports disciplines.** In panels (a), (c), (e), and (g), the parameter $\hat{\alpha}(t)$ i.e., the probability of an extreme upset win is shown. In panels (b), (d), (f), and (h), the parameter $\hat{\beta}(t)$ i.e., the depth of the competition is shown.

## Statistical reliability of prediction accuracies

In Table 4 of the main text, we presented several prediction accuracy scores for each sports disciplines. There, we reported only the accuracy values out to 3 decimal places, as this level of precision sufficed for illustrating their relative performance. To assess whether their differences are statistically significant, the measured values and their corresponding 95% bootstrap confidence intervals are provided in Tables S3-S4. Given that the margin of uncertainty does not exceed $2 \cdot 10^{-3}$, the 3-decimal precision maintained in the main text is statistically justified.

**Men's tennis**

| centrality | FM | SE | LE | AUC |
|---|---|---|---|---|
| 2RC | 0.3454 ± 0.0006 | 0.2194 ± 0.0002 | 0.4367 ± 0.0001 | 0.6572 ± 0.0006 |
| RWC | 0.4502 ± 0.0006 | 0.2447 ± 0.0001 | 0.4885 ± 0.0001 | 0.5503 ± 0.0006 |
| SFS | 0.3563 ± 0.0006 | 0.2242 ± 0.0002 | 0.4473 ± 0.0002 | 0.6444 ± 0.0006 |
| ELO | **0.3436 ± 0.0006** | **0.2134 ± 0.0001** | **0.4256 ± 0.0002** | 0.6569 ± 0.0006 |
| RPR | 0.3446 ± 0.0004 | 0.2171 ± 0.0002 | 0.4330 ± 0.0001 | **0.6575 ± 0.0004** |

**Men's sabre**

| centrality | FM | SE | LE | AUC |
|---|---|---|---|---|
| 2RC | 0.3205 ± 0.0015 | 0.2094 ± 0.0006 | 0.4077 ± 0.0006 | 0.6889 ± 0.0016 |
| RWC | 0.3744 ± 0.0013 | 0.2291 ± 0.0004 | 0.4573 ± 0.0004 | 0.6318 ± 0.0012 |
| SFS | **0.2716 ± 0.0005** | 0.1941 ± 0.0005 | 0.3855 ± 0.0005 | **0.7341 ± 0.0006** |
| ELO | 0.2972 ± 0.0009 | **0.1940 ± 0.0006** | **0.3841 ± 0.0006** | 0.7060 ± 0.0009 |
| RPR | 0.3165 ± 0.0012 | 0.2066 ± 0.0004 | 0.4142 ± 0.0003 | 0.6926 ± 0.0013 |

**Men's épée**

| centrality | FM | SE | LE | AUC |
|---|---|---|---|---|
| 2RC | 0.3601 ± 0.0013 | 0.2239 ± 0.0002 | 0.4453 ± 0.0002 | 0.6492 ± 0.0014 |
| RWC | 0.4123 ± 0.0004 | 0.2387 ± 0.0002 | 0.4753 ± 0.0002 | 0.5933 ± 0.0004 |
| SFS | **0.3143 ± 0.0007** | **0.2134 ± 0.0005** | **0.4281 ± 0.0005** | **0.6921 ± 0.0008** |
| ELO | 0.3428 ± 0.0010 | 0.2153 ± 0.0004 | 0.4318 ± 0.0004 | 0.6598 ± 0.0010 |
| RPR | 0.3588 ± 0.0010 | 0.2249 ± 0.0004 | 0.4493 ± 0.0004 | 0.6503 ± 0.0011 |

**Men's foil**

| centrality | FM | SE | LE | AUC |
|---|---|---|---|---|
| 2RC | 0.3273 ± 0.0019 | 0.2102 ± 0.0003 | 0.4157 ± 0.0005 | 0.6830 ± 0.0019 |
| RWC | 0.3773 ± 0.0005 | 0.2298 ± 0.0003 | 0.4613 ± 0.0004 | 0.6296 ± 0.0006 |
| SFS | **0.2783 ± 0.0005** | **0.1968 ± 0.0004** | **0.3945 ± 0.0004** | **0.7272 ± 0.0006** |
| ELO | 0.3060 ± 0.0008 | 0.1974 ± 0.0002 | 0.3956 ± 0.0002 | 0.6970 ± 0.0007 |
| RPR | 0.3249 ± 0.0010 | 0.2095 ± 0.0005 | 0.4233 ± 0.0004 | 0.6851 ± 0.0010 |

**Table S3.** Prediction accuracy and its 95% confidence interval in men's sports disciplines using different centralities as the "prediction score". The best accuracy value is highlighted in bold for each category and accuracy measure.

**Women's tennis**

| centrality | FM | SE | LE | AUC |
|---|---|---|---|---|
| 2RC | 0.3466 ± 0.0003 | 0.2191 ± 0.0001 | 0.4362 ± 0.0001 | 0.6588 ± 0.0004 |
| RWC | 0.4371 ± 0.0005 | 0.2430 ± 0.0001 | 0.4846 ± 0.0001 | 0.5646 ± 0.0005 |
| SFS | 0.3496 ± 0.0008 | 0.2230 ± 0.0002 | 0.4420 ± 0.0003 | 0.6546 ± 0.0009 |
| ELO | **0.3389 ± 0.0003** | **0.2092 ± 0.0003** | **0.4173 ± 0.0003** | **0.6631 ± 0.0003** |
| RPR | 0.3438 ± 0.0004 | 0.2154 ± 0.0002 | 0.4290 ± 0.0001 | 0.6610 ± 0.0004 |

**Women's sabre**

| centrality | FM | SE | LE | AUC |
|---|---|---|---|---|
| 2RC | 0.3469 ± 0.0018 | 0.2208 ± 0.0003 | 0.4347 ± 0.0003 | 0.6607 ± 0.0020 |
| RWC | 0.4003 ± 0.0012 | 0.2352 ± 0.0004 | 0.4681 ± 0.0004 | 0.6042 ± 0.0013 |
| SFS | **0.2964 ± 0.0009** | 0.2054 ± 0.0006 | 0.4112 ± 0.0007 | **0.7077 ± 0.0010** |
| ELO | 0.3204 ± 0.0014 | **0.2050 ± 0.0003** | **0.4074 ± 0.0005** | 0.6820 ± 0.0013 |
| RPR | 0.3404 ± 0.0010 | 0.2167 ± 0.0003 | 0.4346 ± 0.0003 | 0.6668 ± 0.0012 |

**Women's épée**

| centrality | FM | SE | LE | AUC |
|---|---|---|---|---|
| 2RC | 0.3511 ± 0.0025 | 0.2215 ± 0.0004 | 0.4359 ± 0.0004 | 0.6585 ± 0.0025 |
| RWC | 0.3998 ± 0.0007 | 0.2365 ± 0.0002 | 0.4723 ± 0.0002 | 0.6065 ± 0.0007 |
| SFS | **0.2935 ± 0.0011** | **0.2044 ± 0.0006** | **0.4098 ± 0.0006** | **0.7114 ± 0.0011** |
| ELO | 0.3245 ± 0.0005 | 0.2064 ± 0.0003 | 0.4114 ± 0.0005 | 0.6781 ± 0.0005 |
| RPR | 0.3502 ± 0.0007 | 0.2199 ± 0.0004 | 0.4405 ± 0.0005 | 0.6591 ± 0.0006 |

**Women's foil**

| centrality | FM | SE | LE | AUC |
|---|---|---|---|---|
| 2RC | 0.3285 ± 0.0007 | 0.2135 ± 0.0003 | 0.4175 ± 0.0003 | 0.6816 ± 0.0008 |
| RWC | 0.3713 ± 0.0012 | 0.2308 ± 0.0003 | 0.4546 ± 0.0004 | 0.6358 ± 0.0011 |
| SFS | **0.2785 ± 0.0007** | 0.1978 ± 0.0004 | 0.3942 ± 0.0004 | **0.7263 ± 0.0007** |
| ELO | 0.3038 ± 0.0008 | **0.1971 ± 0.0004** | **0.3902 ± 0.0003** | 0.6991 ± 0.0007 |
| RPR | 0.3264 ± 0.0010 | 0.2099 ± 0.0003 | 0.4209 ± 0.0003 | 0.6832 ± 0.0010 |

**Table S4.** Prediction accuracy and its 95% confidence interval in women's sports disciplines using different centralities as the "prediction score". The best accuracy value is highlighted in bold for each category and accuracy measure.

## The effect of network-building period

During our work, we used the value of $t_{nb} = 1$ year for the network-building period, in line with the fact that in both tennis and fencing, the official ranking is calculated based on performance over the last year. Since this is an completely arbitrary choice, we include in Figures S58-S59 plots about how prediction accuracies (as the most simply testable results in our work) depends on the change of this value to illustrate that the choice of this parameter has not much effect on the results. For tennis, the value of $t_{nb}$ changes between 6 and 18 months, for fencing disciplines, the value of $t_{nb}$ changes between 12 and 36 months, because these are the regimes where there is measurable effect. As Figures S58-S59 show, even within these regimes, the neither of the accuracy scores changes their value more than 5%, but for most of them, there is no significant change at all. We can therefore conclude that a different $t_{nb}$ value would not have an intrinsic effect on our results in the main text.

Note that to change the value of $t_{nb}$ by months, we needed to reduce the time resolution of from 1 year to 1 month. It means that the elementary step with which the sliding window slides during the temporal prediction is much smaller than before, and allows us to get more accurate predictions (at the price of computation). Hence, the accuracy values at $t_{nb} = 12$ month in Figures S58-S59 does not match with the values in Table 4.

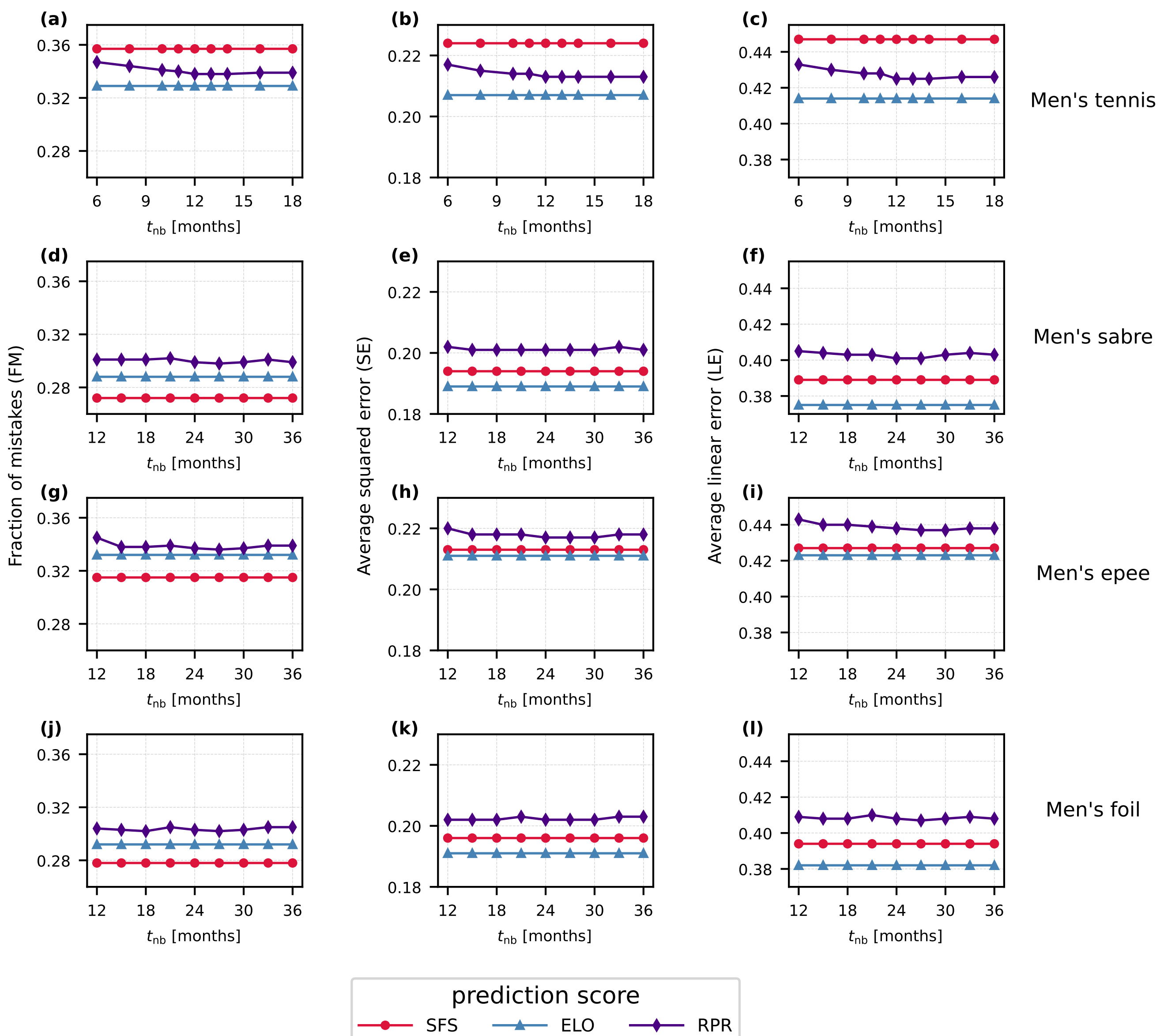


**Figure S58.** **The prediction accuracy values in the function of the network-building period for men's sport disciplines.**

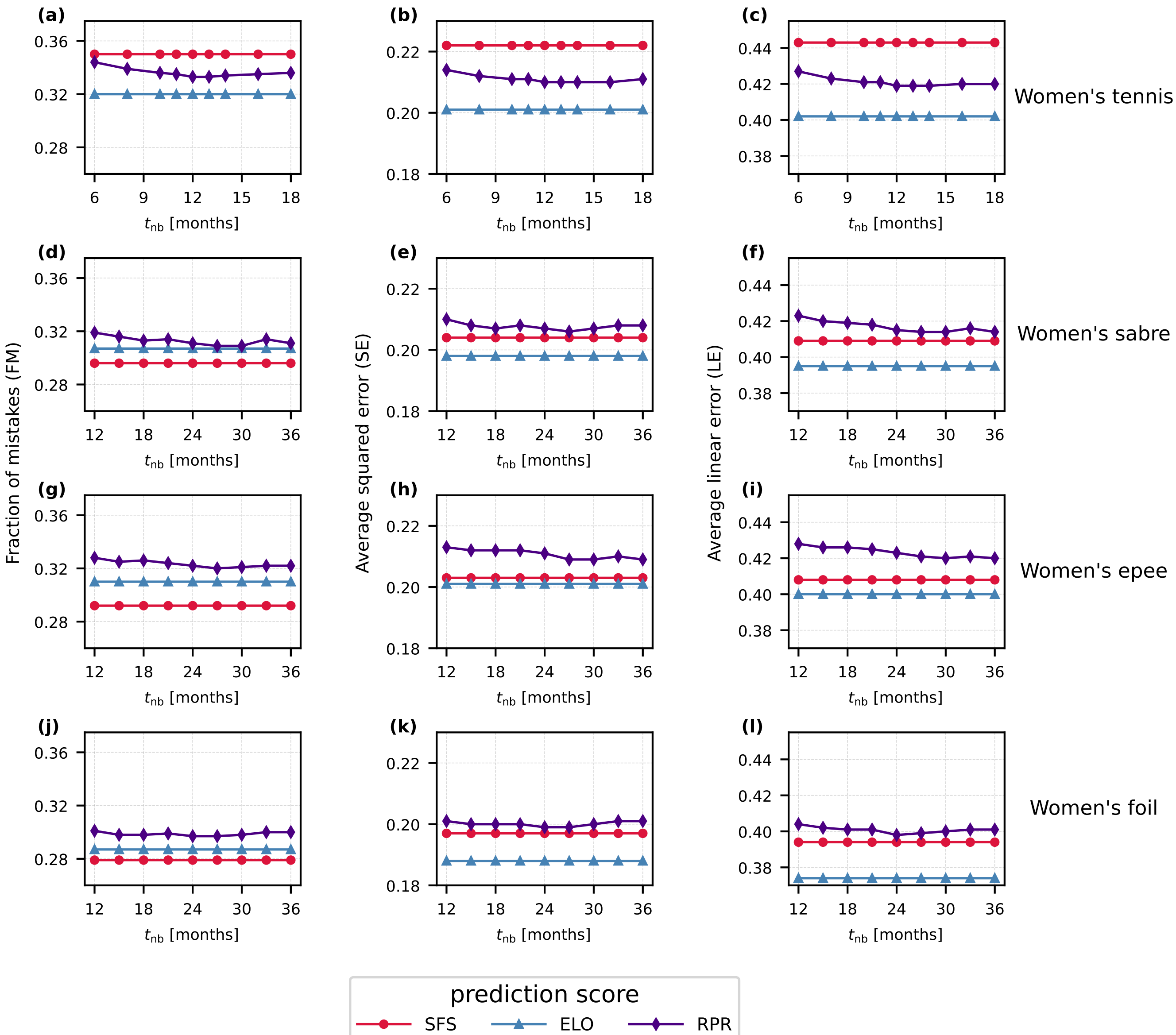


**Figure S59.** **The prediction accuracy values in the function of the network-building period for men's sport disciplines.**
